# Vibrational Spectra Modeling of Cellulose Nitrate During Initial Photodegradation Stage by Density Functional Theory: The Case of Ketone Formation

**DOI:** 10.3390/molecules31162890

**Published:** 2026-08-19

**Authors:** Dmitrii Pankin, Maksim Moskovskiy, Anastasia Povolotckaia

**Affiliations:** 1 Center for Optical and Laser Materials Research, Saint-Petersburg State University, Universitetskaya Nab. 7/9, Saint-Petersburg 199034, Russia; dmitrii.pankin@spbu.ru; 2 Federal Scientific Agroengineering Center VIM, Moscow 109428, Russia; maxmoskovsky74@yandex.ru

**Keywords:** cellulose nitrate, density functional theory, Raman spectroscopy, IR absorption spectroscopy, cluster, photodegradation

## Abstract

The study of degradation processes is of fundamental and applied interest. To understand the degradation of cellulose nitrate and to develop sensitive diagnostic methods, combined experimental and theoretical investigations are essential. Sensitive, non-destructive, and contactless methods for diagnosing the state of cellulose nitrate include IR absorption and Raman spectroscopy. While significant experimental work exists, the theoretical modeling of degradation processes, including the prediction of potential products, remains underdeveloped. Therefore, in this work, the structures and vibrational properties of molecular clusters representing segments of the cellulose nitrate chain were modeled using density functional theory (DFT). This approach yielded simulated IR and Raman spectra, allowing for the identification of peaks corresponding to the nitrate group. The elimination of this group to form a ketone was shown to alter peak contours across a broad spectral range. The most significant changes in the Raman spectra were observed at 605 and 851 cm^−1^. Correspondingly, the key changes in the IR absorption spectra occurred at 836, 1034, 1169, 1283, and 1767–1781 cm^−1^. The frequency trends for these diagnostic peaks across different model structures were analyzed and compared with experimental spectra from the literature. The demonstrated correlation between specific peak-frequency changes and the modeled degradation products constitutes the principal novelty of this work.

## 1. Introduction

The study of cellulose nitrate degradation processes is an important research objective for several reasons. Firstly, this material was historically used to produce objects that now hold significant historical value. These include photographs, negatives, and cinematographic films capturing images of people, architecture, and landscapes [1,2,3,4]. Such materials serve as witnesses to bygone eras and enable detailed preservation for historical reconstruction and the study of past living conditions [1,2,3,4,5,6,7]. Works of art, such as films shot on cellulose nitrate-based stock, are of comparable importance [8].

Furthermore, everyday items manufactured from cellulose nitrate are also of historical interest, including billiard balls [9,10], celluloid postcards and calendars [11], goggles from World Wars I and II [12], dental prostheses [9], and ivory-like aero fans [13].

In this context, a critical task for preserving cultural heritage is the timely identification of degradation and the implementation of conservation measures. This requires studying how an object’s state manifests at various degradation stages [14], especially at the initial stages, which is crucial for diagnosing historical artefacts and determining the underlying chemical reactions.

Such diagnostics are also valuable for contemporary objects containing this polymer. Today, cellulose nitrate is used in military applications with a high degree of nitration [15], and in civilian applications with a lower degree of substitution. Examples of the latter include collodion for wart treatment and mixed cellulose acetate/nitrate membrane filters in the pharmaceutical industry [16].

Promising non-invasive and non-contact techniques for assessing polymer state, especially for cultural heritage objects, are optical methods. These include vibrational spectroscopy [5,7,12,13,15,17,18,19], UV-visible reflectance/absorption spectroscopy [14,20,21], luminescence spectroscopy [11], and multispectral imaging techniques, including hyperspectral and RGB imaging [5,6].

Spectroscopic techniques based on electronic phenomena can be challenging to interpret and may obscure dependencies in the polymer’s electronic structure during degradation. The task becomes more complex for layered historical objects, such as film stocks [11] and photographs [18]. This necessitates mathematical processing and chemometric methods to uncover hidden patterns. An example is the derivative method for analyzing the UV-visible absorption edge to resolve overlapping electronic transition bands in pristine and degraded polymer [14,21]. Chemometric analysis with machine learning has also been applied to identify areas of varying degradation in photographic negatives [5,6].

In contrast, vibrational spectroscopy yields more informative spectra where peak frequencies are closely linked to molecular structure. Chemometric analysis [7,18] can correlate spectral regions in loading plots with the principal components of data variability.

It should also be noted that NMR spectroscopy (^1^H, ^13^C, ^15^N) of cellulose nitrate and related model systems [22,23], as well as X-ray diffraction studies of highly substituted cellulose nitrate [24], has played a substantial role in elucidating its structure.

Nevertheless, vibrational spectroscopy is considerably simpler to implement experimentally. Its non-destructive nature is a significant advantage, particularly for cultural heritage objects with strict preservation requirements. Historically, infrared absorption spectroscopy has been used more intensively. The first interpretations of IR absorption spectra date to 1969–1970 [25,26] and provide a detailed peak interpretation supported by dichroic ratios and ^15^N isotopic substitution data.

Applied to real objects, degradation processes in cellulose nitrate bases have been studied using IR spectroscopy in works such as [15,17,18,19]. A complementary technique is Raman spectroscopy, which also allows for non-invasive, non-contact investigation. The strong intrinsic Raman activity of cellulose nitrate enables the study of its preservation state even under thick top layers of other materials, such as gelatin [5,7,18], as well as in various non-layered, volumetric objects [12,13].

Currently, a considerable volume of empirical vibrational spectral data exists. Studies using IR and Raman spectroscopy have identified the most prominent cellulose nitrate peaks, demonstrated spectral differences from cellulose acetate [17], and established degradation markers [15,17,18,19]. These markers are often based on peak ratios, such as increased v(C=O) absorption relative to decreased N–O and O=N=O stretching vibrations [19], or the degree of nitration based on the ν_a_(NO_2_)/ν(COC) ratio [17].

This has led to proposed degradation mechanisms for cellulose nitrate under thermal and UV photodegradation, based on vibrational spectroscopy and other methods (primarily NMR). According to [15], light-induced degradation has been less studied than thermal degradation.

Another advantage of vibrational methods over electronic optical methods is the possibility of calculating vibrational properties using modern quantum-chemical methods, for example, within cluster model approaches. It should be noted that relatively few up-to-date theoretical studies of cellulose nitrate using such methods exist. An example is [27], which studied the applicability of a cluster approach for modeling structural, energetic, and conformational properties, as well as degradation mechanisms. Additionally, ref. [28] theoretically demonstrated the possibility of complex formation between cellulose nitrate and heavy metal ions (Pb^2+^ and Hg^2+^) in model clusters, discussing the influence on frontier orbitals, selected vibrational frequencies, and energetic and structural parameters.

A characteristic feature of the quantum-chemical modeling approach is its ability to simulate systems with varying degrees and positions of nitration, as well as nitrate group removal. Reproducing such systematic variations experimentally is technologically challenging, making theoretical modeling a direct and viable method for studying these processes. However, the conformational flexibility of the polymer, potential nitration inhomogeneity, and various possible degradation mechanisms-at different stages leading to ketones at initial stages, as well as to gluconolactone and anhydrides at further stages [15]-result in a large number of possible model variants. Therefore, describing observed structure-spectrum correlations requires focusing on a specific case.

Accordingly, this article takes a first step toward modeling initial-stage degradation processes and establishing structure-spectrum correlations using density functional theory (DFT). The objective is to investigate clusters modeling the structural and vibrational properties of cellulose nitrate with varying degrees of nitration. Models with maximum substitution in each ring and with one maximally substituted ring are considered to separate contributions from substituted and unsubstituted regions. Furthermore, this work aims to study spectral changes upon the removal of nitrate groups to model ketone formation during initial stages of photodegradation, which constitutes its novelty. The theoretical results are discussed in conjunction with existing experimental FTIR and Raman spectroscopy data. The applicability and limitations of the investigated models are also discussed in the corresponding sections.

## 2. Results

Experimental studies have established that exposing nitrocellulose to electromagnetic radiation with a wavelength exceeding 300 nm initiates its photodegradation mechanism, as demonstrated in [19,29]. Furthermore, time-resolved investigation of model samples under irradiation (λ > 280 nm) via FTIR absorbance and Raman spectroscopy revealed significant alterations in band contours and the appearance of new peaks [30].

According to [11], the initial stage of photodegradation involves the cleavage of nitrate groups, leading to the formation of anhydride and lactone structures. This process is accompanied by the emergence of a band in the IR absorption spectra within the C=O stretching region. The frequencies of these vibrations are highly sensitive to the local chemical environment and can manifest across a broad spectral range [31]. For instance, a peak at approximately 1740 cm^−1^ was observed in FTIR spectra after 50 h of irradiation in a study employing both IR and Raman spectroscopies [30]. The band maximum reported in [30] is broad, while in [29], the carbonyl (C=O) stretching band for model cellulose nitrate and celluloid samples exhibits a complex contour consisting of several poorly resolved components. This spectral complexity likely indicates the formation of multiple degradation products. In this regard, theoretical consideration of model structures can facilitate the conscious decomposition of complex spectral contours into individual peaks and facilitate subsequent analysis of changes in the areas of these peaks, which are closely related to the bonds formed and thus allow for insight into the underlying mechanism. Thus, theoretical modeling and the trends it reveals contribute to a deeper understanding of ongoing degradation processes, allowing experimenters to more accurately interpret their experimental data.

**Figure 1:**
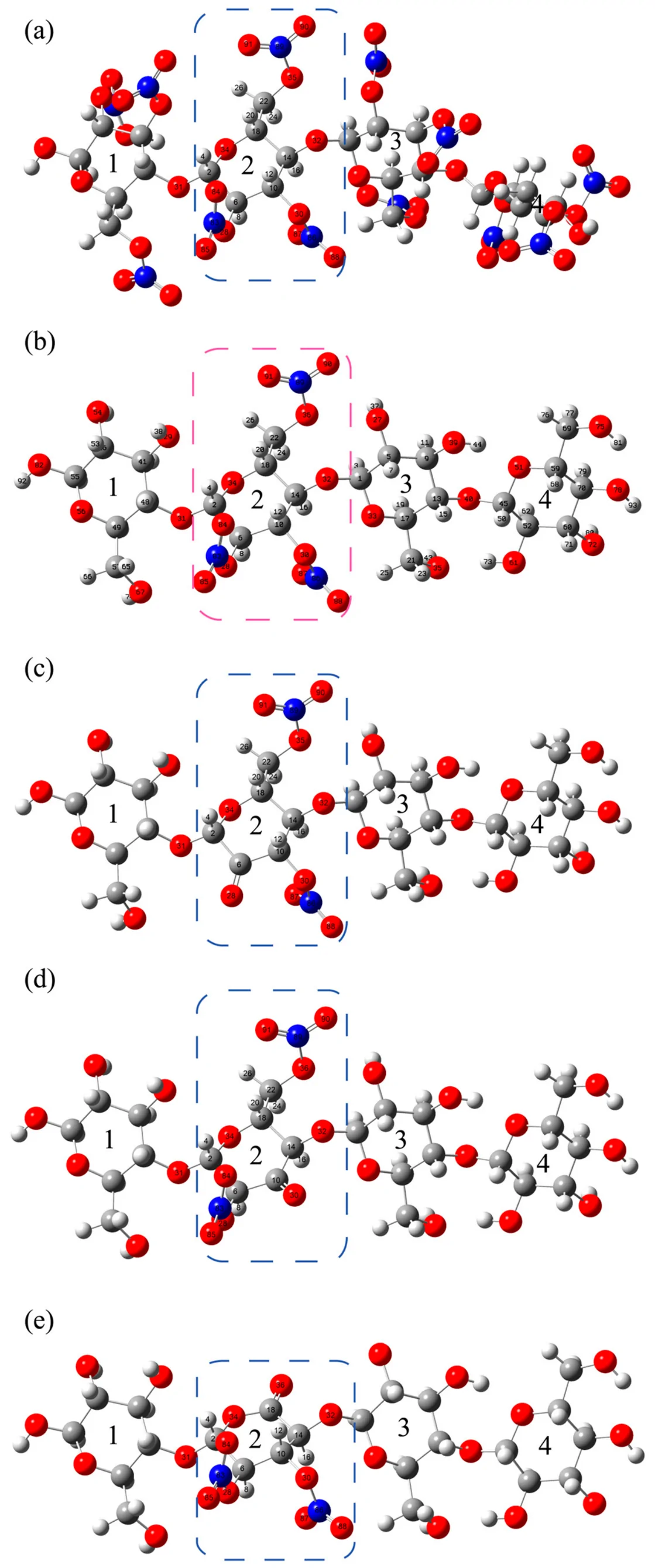
Structural models: NC model (**a**), Model 1 (**b**), Model 2 (**c**), Model 3 (**d**), and Model 4 (**e**). The pyranose ring numbering is present in the center of each ring for each structure. The atom numbering used here and in the text and Table 1 is demonstrated in (**b**). The pyranose ring discussed in the text is highlighted with the dashed line.

Based on the detailed summary of photodegradation mechanisms and potential products for cellulose nitrate provided in [15], several model structures were selected for investigation of their structural and vibrational properties using quantum-chemical methods. Their optimized geometries are presented in Figure 1. For the calculations, the exchange-correlation functional B3LYP with the split-valence triple-zeta basis sets 6-311G(2d,p) was employed. The environment simulating interchain interactions was implicitly accounted for using a polarizable continuum model to prevent surrounding molecules from contributing to the simulated spectrum of the degradation product. This primarily avoids mixing and shifting in cases of closely spaced vibrational frequencies. The computational details are described in Section 3.1, “Theoretical Approach.”

### 2.1. Structural Peculiarities

The model structures are based on pyranose rings with varying degrees of nitration. In the structure depicted in Figure 1a,b, all three hydroxyl groups of ring 2 are substituted by nitrate groups. The cellulose nitrate model with the maximum degree of substitution (hereafter called the NC model) is shown in Figure 1a. In this model, all four pyranose rings are fully substituted, corresponding to the theoretical limit of the degree of substitution parameter, γ = 3.

Industrially produced nitrocellulose typically has a degree of substitution in the range of 2.0 < γ < 2.7. This indicates that, on a molecular level, incomplete substitution within individual monomer units or an alternation of fully and partially substituted monomers may occur.

Therefore, this article also considers the case of a single, fully nitrate-substituted pyranose ring placed between unsubstituted rings (Figure 1b, hereafter Model 1). This choice is justified not only by its relevance to common substitution levels but also by its utility for assessing the influence of adjacent unsubstituted rings. In this configuration, each nitrate group position is unique, meaning its detachment would produce the strongest spectral contrast. The model also isolates vibrational modes not associated with nitrate groups and facilitates a comparison of how the structural parameters of the substituted ring are affected by its chemical environment.

The bond lengths in the NC model and Model 1 are quite similar. The most significant differences are observed for the following bonds: C6-O28 (longer in Model 1), and C14-O32 and O36-N89 (shorter in Model 1). The largest differences in bond angles occur near the methyl nitrate moiety (angles C22-C18-O34, O36-C22-C18, O34-C18-C14) and the nitrate group with N83 (angles C10-C6-C2, O28-C6-C2). In the NC model, neighboring nitrate groups interact with each other in adjacent rings. In contrast, in Model 1, these groups are located near the OH groups of adjacent rings. In both models, the calculations predict that one oxygen atom in each of the N83O3 and N86O3 nitrate groups is oriented towards the two axial oxygen atoms of the pyranose ring.

Although the uncertainties for atomic positions or bond lengths are not provided in reference [24], a comparison between the experimental values and our computational results reveals several notable features. For single carbon-carbon bonds within the pyranose ring and at the interface with the methyl group C22H2, the calculated lengths are slightly overestimated by 0.011–0.016 Å in both the NC model and Model 1. Conversely, the calculated C-O bond lengths within the pyranose ring are lower than the experimental values. A different trend is observed for the glycosidic bridges, where the calculated lengths of bonds C14-O32 and C2-O31 exhibit a different relative order compared to the experimental data. At the interface between the pyranose ring and the nitrate groups (N83O3 and N86O3), the calculated carbon-oxygen bond lengths are overestimated. In contrast, for the methyl-nitrate moiety, the calculated values are close to the experimental data. Regarding the nitrate groups, the theory predicts a significantly longer bond length for the single N-O bond (1.413–1.420 Å) compared to the experimental value of 1.371–1.372 Å reported in [24]. Conversely, the bonds in the NO_2_ group are predicted to be shorter (1.198–1.204 Å) than the experimental values and are not equivalent: the N=O bond oriented towards the axial hydrogens is calculated to be shorter (Table 1). For instance, the calculated distances for the interactions N83-O84---H4 and N83-O84---H12 are 2.417 Å and 2.347 Å, respectively, while for N86-O87---H8 and N86-O87---H16 they are approximately 2.374 Å.

According to experimental data in [24], the bond angles within the pyranose ring—with the exception of C2-O34-C18—are similar to each other and close to 110°. In contrast, the C2-O34-C18 angle is slightly larger at 112.3°. The calculations predict greater variability in these angles. Specifically, the largest angles are predicted for C2-O34-C18 and C14-C10-C6, while the smallest is for C6-C2-O34. This suggests the theoretical model yields a more non-uniformly bent ring.

For the nitrate groups, the calculated angles increase in the series C-O-N < O-N=O < O=N=O, which qualitatively agrees with experimental observations.

When comparing the predicted bond lengths in Model 1 with those in Models 2–4, significant changes in the C-O bond lengths near the newly formed ketone group are evident. Relative to Model 1, the C2-O34 bond in Model 2 lengthens (the Mulliken charge on atom C2 decreases), while bonds C10-O30 and C2-O31 shorten (the Mulliken charge on C10 decreases and on O30 increases). In Model 3, bonds C6-O28 and C14-O32 shorten (the Mulliken charge on C6/C14 decreases and on O28/O32 increases, respectively). In Model 4, bonds O34-C18 and C14-O32 shorten (the Mulliken charge on C14 and O32 increases), while bond C2-O34 lengthens (the Mulliken charge on C2 and O34 increases).

Thus, for bonds that shorten (with the exception of O34-C18 in Model 4), a general trend is observed: a decrease in positive charge on the carbon atom and an increase in the Mulliken charge on the oxygen atom. In Model 4, a redistribution of the lengths of bonds C2-O34 and O34-C18 is observed compared to Model 1, an effect that is less pronounced in Model 2. In Model 3, due to the greater distance from the ketone group, the lengths of these bonds remain practically unchanged. In all Models 2–4, the calculated C=O bond length is approximately 1.2 Å.

In Models 2 and 3, replacing a nitrate group with a ketone group leads to a lengthening of the N-O bond in the remaining adjacent nitrate group. In Model 4, replacing the methyl-nitrate group does not cause significant changes in the structural parameters of the remaining groups.

For Models 2 and 3, the pyranose ring angles show relatively minor differences (<2.5°) compared to Model 1. The largest change is observed for the C14-C10-C6 angle. The most significant change for O-C-C angles involving an exocyclic atom is for the angle involving the ketone group. For Models 2 and 3, these are O28-C6-C2 (+9.5°) and O30-C10-C6 (+8.4°), respectively. The angle values for the remaining nitrate group near the ketone in Models 2 and 3 are similar. No substantial differences are observed for the angles of the more distant methyl-nitrate group.

More significant changes in the angles of the pyranose ring are predicted for Model 4 (Table 2). Increases of +12° and +10° are noted for C2-O34-C18 and O34-C18-C14, respectively, in addition to the aforementioned substantial shortening of the O34-C18 bond. The angle involving the newly formed ketone group, O34-C18-C22, in Model 4 is predicted to be 119.5°, which is closer to the analogous angles O28-C6-C2 and O30-C10-C6 in Models 2 and 3 than to the original value in Model 1.

### 2.2. IR Absorbance Spectroscopy Study

**Figure 2:**
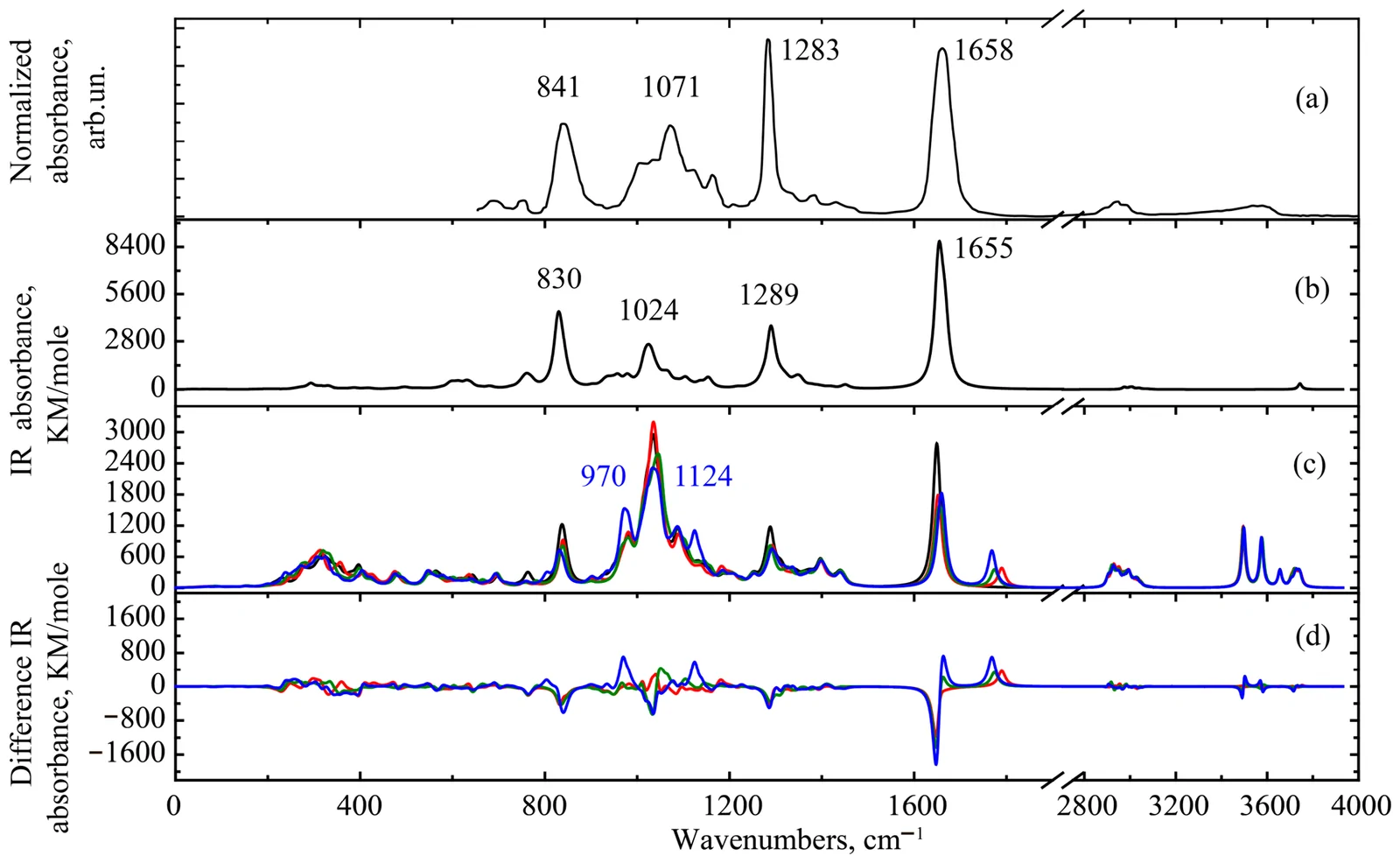
Experimental spectrum of historical cellulose nitrate sample (**a**) and calculated scaled infrared (IR) absorption spectra for: (**b**) the NC model; (**c**) model structures 1 (black), 2 (red), 3 (green), and 4 (blue); (**d**) difference scaled IR absorption spectra, obtained by sequentially subtracting the scaled IR absorption spectrum of model 1 from the scaled spectra of model 2 (red), model 3 (green), and model 4 (blue).

Figure 2 presents the experimental and calculated infrared (IR) absorption spectra for the NC model and Model structures 1–4, along with their corresponding difference spectra. The spectra for the NC model and Model 1 represent two limiting cases: the first corresponds to a degree of substitution of 3 in all rings, while the second corresponds to a low overall degree of substitution that is localized within a single pyranose ring. Consequently, in the latter case, the nitrate groups are surrounded by the hydroxyl groups of adjacent rings.

Comparing the calculated IR absorption spectra for Model 1 and the NC model with experimental data allows for the identification of spectral regions containing peaks predominantly related to substituted and unsubstituted structural motifs. A peak’s insensitivity to the replacement of a nitrate group with a ketone group further confirms its origin in an unsubstituted region.

**Figure 3:**
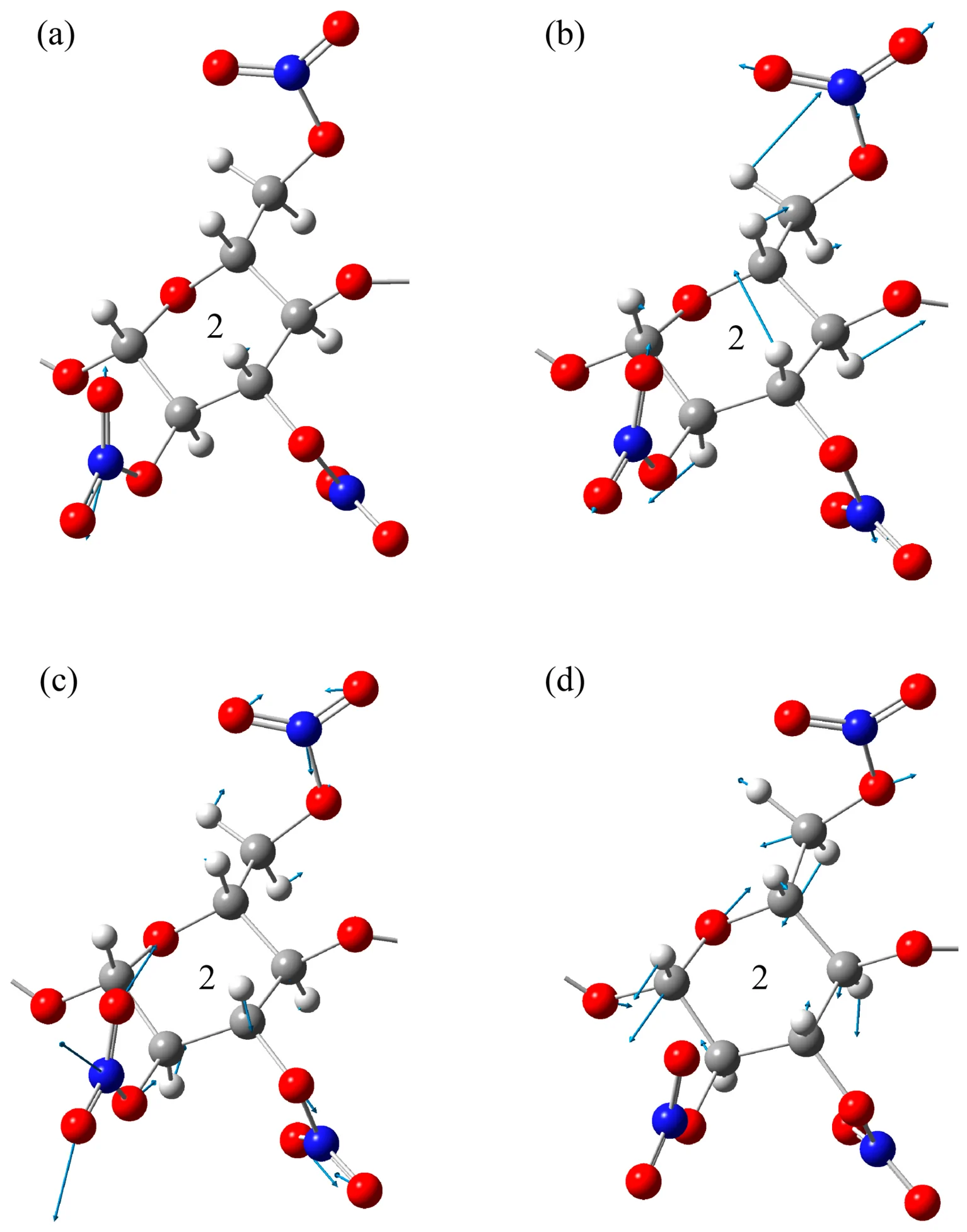
Example of atomic displacements in ring 2 of NC model in selected modes 293 (**a**), 236 (**b**), 161 (**c**) and 188 (**d**). For ring numbering see Figure 1.

The absence of peaks with a maximum in the region above 1700 cm^−1^ was characteristic of the non-degraded samples [15,19,30]. In the IR absorbance spectrum of the historical cellulose nitrate sample, the presence of bands with maxima at 1658, 1283, 1071 and 830 cm^−1^ was noted. In the IR absorption spectrum of the NC model (Figure 2b), four highly active peaks can be distinguished at 1655, 1289, 1024, and 830 cm^−1^. Their interpretation is as follows. The peak at 1655 cm^−1^ is attributed to the antisymmetric stretching vibration of the NO_2_ group (v_as_(NO_2_)). An example of atomic displacements in ring 2 in mode No. 293 (scaled frequency 1657 cm^−1^) is shown in Figure 3a. This peak has dominant IR activity in the modelled absorbance spectrum. In the experiment it is highly active and well separated from others [30]. The width of this peak in the experimental spectrum is due to interactions through the highly polarized N=O bonds and the slightly different environments of these bonds. This makes it broader and comparable to, or slightly lower in intensity than, the peak around 1280 cm^−1^ [15,30]. In the theoretical spectrum the contour of the 1655 cm^−1^ band is asymmetric, arising from a large number of contributing vibrational modes with atomic displacements localized in various rings with different environments. The band at 1289 cm^−1^ is formed by two types of vibrations: symmetric stretching of the NO_2_ group (v_s_(NO_2_)) and several hydrogen-related modes, including δ(XCH) (where X = C or O), w(HCH), and τ(HCH). An example of atomic displacements in ring 2 in mode No. 236 (scaled frequency 1290 cm^−1^) is shown in Figure 3b. This interpretation expands and refines that given in previous experimental studies. It shows that an intersection of the regions of v_s_(NO_2_) and some hydrogen-related modes occurs. This point may be important for cellulose nitrates with a low degree of substitution, and may also explain the broad absorption baseline in this region in the IR absorbance spectrum [15,30] along with the narrow v_s_(NO_2_) peak. In the NC model, the peak at 830 cm^−1^ corresponds to vibrations predominantly localized in the NO_3_ groups, representing coupled N-O stretching and O=N=O bending vibrations within the same group. An example of atomic displacements in ring 2 in mode No. 161 (scaled frequency 833 cm^−1^) is shown in Figure 3c. The number of vibrational modes forming the contour of the 830 cm^−1^ band was 12 for the NC model and 3 for Model 1, which coincides with the number of NO_3_ groups in each model. However, in individual modes, the atomic displacements were delocalized across several groups. The corresponding experimental peak in the IR absorbance spectra is also clearly observable in references [15,19,30].

Another peak associated with the NO_3_ group is formed by vibrational modes around 760 cm^−1^. Their characteristic feature involves out-of-plane motions of the nitrogen atom with respect to the plane of the oxygen atoms, coupled with deformation of the O=N=O bonds. This is typical of the atomic displacements of the ν_2_ mode in the NO_3_^−^ ion [32]; see, for example, mode No. 149 in the NC model in Figure S1.

The peak at 1024 cm^−1^ is associated with vibrational modes involving stretching-type atomic displacements delocalized across various C-O bonds, including those within the pyranose ring, glycosidic bridges, and C-O bonds directly linked to nitrate groups or methylene groups. Minor hydrogen displacements were noted in the region. An example of atomic displacements in ring 2 for mode No. 188 (scaled frequency 1023 cm^−1^) is shown in Figure 3d. The attribution is largely in agreement with the interpretation of the bands in the 1000–1060 cm^−1^ region in [30]. In Model 1, such modes also include contributions from C-OH stretching and, to a lesser extent, C-C bond stretching. The greater relative number of C-OH bonds in Model 1 leads to a relatively higher IR intensity for this mode compared to the NC model. Consequently, this peak shows only a minor decrease in IR intensity upon replacement of a nitrate group with a ketone, as seen in Figure 2b,c. However this decrease coincides with the degradation trend of IR bands reported in [15]. Comparison of the calculated spectra for the NC model and Model 1 with the experimental spectrum obtained from the historical sample allows the interpretation of the modes to be confirmed. Specifically, the relative contribution of the nitrate group peaks at 830, 1289, and 1655 cm^−1^ in the NC model, as the limiting case of maximum substitution (γ = 3), is rather large (Figure 2b). At the same time, the contribution from stretching vibrations within the pyranose rings and at their boundaries is substantially higher relative to the contribution from the nitrate groups in Model 1 (Figure 2c). In this respect, the experimental spectrum in Figure 2a is intermediate in terms of this ratio. A comparison of the experimental spectrum (Figure 2a) with the theoretical spectra for the NC model and Model 1 shows rather close agreement for the frequencies of the nitrate groups. The frequencies of the stretching vibrations in the pyranose rings are underestimated with the present scaling and with the implicit treatment of the environment via the continuum model.

In order to demonstrate more clearly the trends in IR absorbance upon transition from one model structure to another, the difference IR absorbance spectra were additionally plotted (Figure 2c). In such spectra, the negative ordinate values correspond to a decrease in IR absorbance for models 2–4 as compared with Model 1. Lower IR intensity in Models 2–4 is mainly observed for other peaks associated with nitrate groups, namely those at approximately 760, 830 (839 cm^−1^ in Model 1), 1289, and 1655 cm^−1^ (1648 cm^−1^ in Model 1). This trend is in agreement with the changes reported in experimental IR absorbance spectra in [15] for the corresponding peaks at 1650, 1280, 1070, and 840 cm^−1^.

Furthermore, progressing from Model 2 to Model 4, the calculated peak frequency decreases from 839 to 831 cm^−1^, increases from 1652 to 1660 cm^−1^, and the ν(C=O) stretching peak decreases from 1790 to 1769 cm^−1^. The ν(C=O) peak is specific to Models 2–4 and thus appears as a positive feature in the difference spectrum (Figure S2). This is in agreement with the positive trend of the 1740 cm^−1^ peak in [15] and its growth in [30]. Additionally, calculations for Model 4 predict specific spectral regions around 970 cm^−1^ (mode No. 113, Figure S3) and 1124 cm^−1^ (mode No. 147, Figure S4) where absorption is greater than in Models 1–3. Mode No. 113 exhibits substantial atomic displacements in the C2-O34 and O34-C18 bonds, while mode No. 147 shows a greater degree of delocalization across various C-O bonds in this frequency range, accompanied by C-C stretching and bending XCH vibrations.

For vibrational modes where atomic displacements are unrelated or only weakly related to nitrate groups, the difference spectra yield relatively small values. This is evident in regions associated with hydrogen stretching and deformation vibrations, hydrogen wagging and twisting modes, and torsional vibrations of hydrogen groups, as well as deformation vibrations of the pyranose ring and glycosidic bridges (the 250–450 cm^−1^ range).

The relatively minor decrease in the intensity of the 1289 cm^−1^ peak when a nitrate group in Model 1 is replaced by a ketone in Models 2–4 (Figure 2b) further reflects the significant hydrogen-related contribution in this spectral region.

### 2.3. Raman Spectroscopy Study

**Figure 4:**
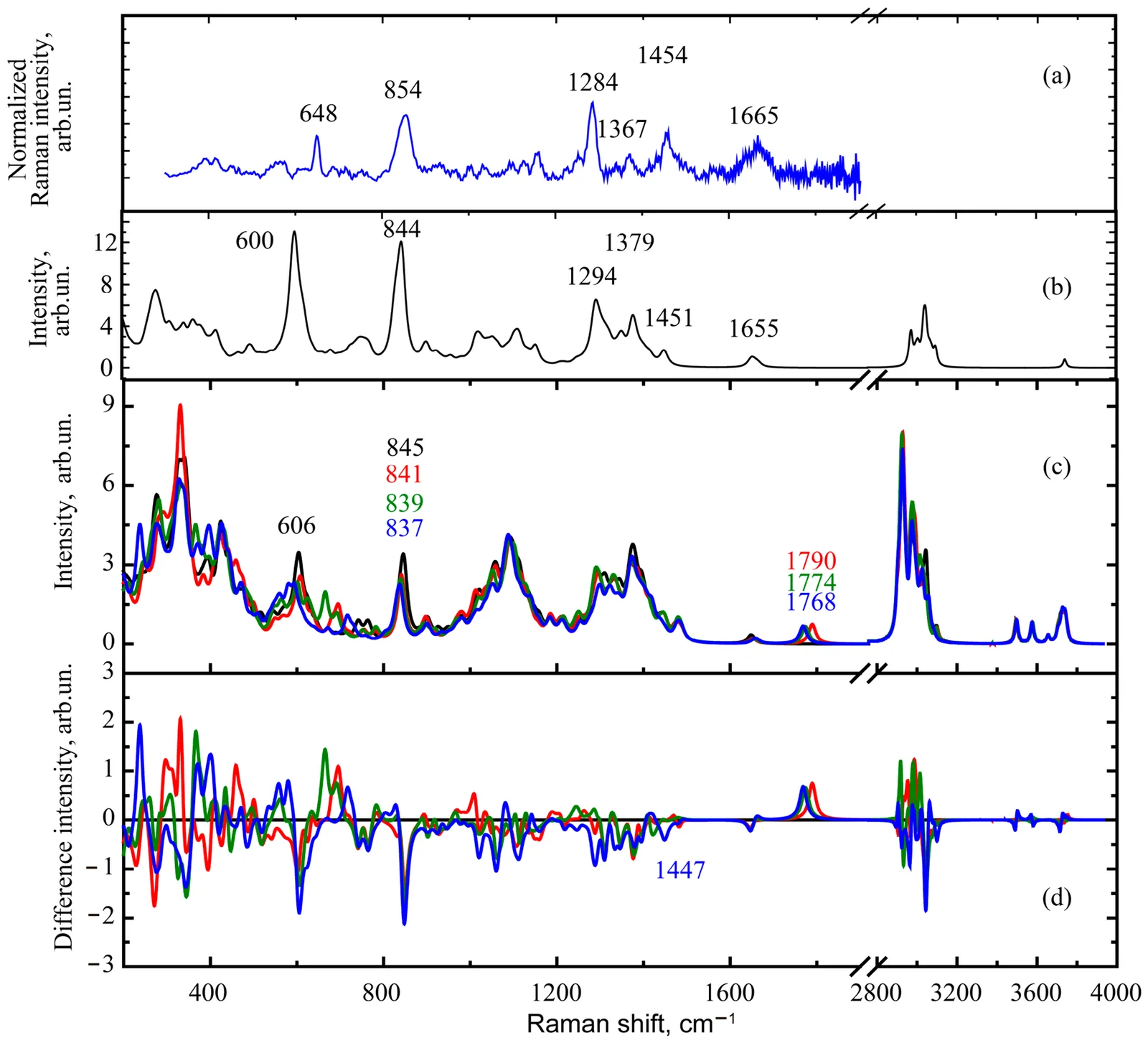
(**a**) Experimental spectrum of historical cellulose nitrate sample; (**b**) calculated scaled Raman spectra for NC model and (**c**) calculated scaled Raman spectra for model structures 1 (black), 2 (red), 3 (green), and 4 (blue); (**d**) difference scaled Raman spectra, obtained by sequentially subtracting the scaled Raman spectrum of model 1 from the scaled spectra of model 2 (red), model 3 (green), and model 4 (blue).

To evaluate the prospects for early-stage degradation detection via Raman spectroscopy, the differences between the Raman spectra of Model 1 and those of Models 2–4 were examined. Analogous to the approach for IR spectroscopy, emphasis was placed on vibrational modes localized in or near the second ring-specifically, the 2,3,6-trinitro-substituted pyranose ring in Model 1 and the corresponding ketone-substituted ring in Models 2–4. The case of non-resonant Raman scattering excited by monochromatic 785 nm radiation was considered. This excitation wavelength was chosen to minimize potential luminescence, which is more likely with visible or UV radiation. Details regarding the conversion of Raman activity to intensity are provided in Section 3.1, Materials and Methods. The calculated scaled Raman spectra are presented in Figure 4.

**Figure 5:**
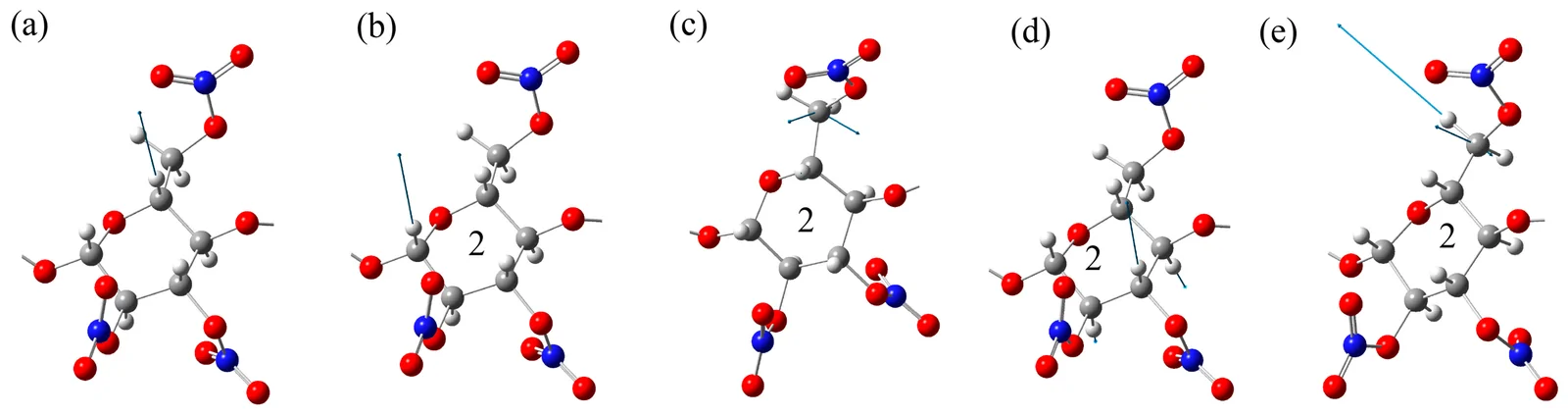
Example of atomic displacements in ring 2 of NC model in selected modes 299 (**a**), 305 (**b**), 309 (**c**), 312 (**d**) and 323 (**e**). For ring numbering see Figure 1.

In the 2800–3000 cm^−1^ range, as noted in references [13,30], the Raman spectrum of pristine cellulose nitrate exhibits two resolved peaks (2976 and 2908 cm^−1^ [30]), which merge into a single peak as degradation proceeds. For the NC model (Figure 4b), the presence of two maxima was also observed in the region of C–H stretching vibrations. In the calculations, these are represented by vibrational modes nos. 298–325 (Table S6). For the NC model, a clear grouping of modes with C–H stretching vibrations is observed, depending on the localization of the vibrations. The lowest frequencies correspond to modes No. 298–301, which are associated with axial vibrations of hydrogens in the CH_2_NO_3_ moiety. An example of atomic displacements in ring 2 for mode No. 299 is shown in Figure 5a. Higher frequencies are exhibited by modes No. 302–307, which are associated with axial stretching vibrations of hydrogens at the glycosidic bridge (see the example in ring 2 in Figure 5b). For modes No. 308–321, symmetric stretching vibrations in the CH_2_ group of the CH_2_NO_3_ moiety are observed, as well as delocalized axial C–H vibrations. A characteristic feature of the latter is that one of the hydrogen atoms is located in close proximity to an NO_3_ group. An example of atomic displacements in ring 2 for modes No. 309 and 312 is shown in Figure 5c and Figure 5d, respectively. Modes No. 322–325 originate from antisymmetric vibrations in the CH_2_ group of the CH_2_NO_3_ moiety (see the example in ring 2 in Figure 5e).

For the 1–4 Models, the more significant differences in the spectral band contours within the 2800–3600 cm^−1^ region occur in the C–H stretching range rather than in the O–H stretching bands, as evident in the difference spectrum in Figure 4b.

The differences in the predicted Raman spectra of Models 2–4 compared to Model 1 arise primarily from the presence of a ketone group and, in Model 4, the additional absence of a methylene group. This leads to altered axial hydrogen stretching vibrations in the ring and the loss of a contribution from methylene group stretching modes in Model 4. For Model 1, the calculations predict a set of vibrational modes including mode No. 261 (3076 cm^−1^), mode No. 260 (3058 cm^−1^), mode No. 258 (3046 cm^−1^), mode No. 257 (3041 cm^−1^), mode No. 256 (3036 cm^−1^), and mode No. 246 (2968 cm^−1^). The intense modes No. 257 (3041 cm^−1^) and No. 262 (3099 cm^−1^) correspond to symmetric and asymmetric hydrogen stretching vibrations, respectively, in the methylene group attached to the nitrate moiety. The remaining intense modes, including the strongest one (No. 258 at 3046 cm^−1^), are localized within the 3076–3036 cm^−1^ range.

In Model 2, the ν_s_(CH_2_) and ν_as_(CH_2_) modes of the methylene group connected to the nitro group are observed at approximately the same frequency region as in Model 1: 3040 cm^−1^ (No. 250) and 3099 cm^−1^ (No. 247). Stretching vibrations distant from the ketone group are also similar, appearing at 3062 cm^−1^ (No. 249) and 2969 cm^−1^ (No. 236). In contrast, the axial stretching vibrations with the largest atomic displacements near the ketone group exhibit a significant frequency shift to 2992 cm^−1^ (No. 241) and 2952 cm^−1^ (No. 234).

For Model 3, the frequencies of ν_s_(CH_2_) and νₐ_s_(CH_2_) connected to the nitro group are within 2 cm^−1^ of those in Model 2. Meanwhile, the axial stretching vibrations near the ketone group are observed at 3014 cm^−1^ (No. 244), 2985 cm^−1^ (weak, No. 240), and 2972 cm^−1^ (No. 236).

The Raman spectrum of Model 4 exhibits qualitative differences compared to Models 2 and 3. First, the absence of the CH_2_NO_2_ group eliminates contributions near 3040 and 3099 cm^−1^. Combined with shifts in other modes, this results in more negative values in the Raman difference spectra within the 3040–3045 cm^−1^ range (Figure 3b) than in the difference spectra for Models 2 and 3. Second, the proximity of several oxygen atoms in addition to the ketone oxygen leads to increased frequencies for several axial hydrogen vibrations. Specifically, vibrational modes localized in the second ring have frequencies of 3085 cm^−1^ (No. 241), 3071 cm^−1^ (weak, No. 240), 3059 cm^−1^ (No. 239), and 3024 cm^−1^ (No. 236).

Overall, for Models 2 and 3, the presence of low-frequency shifts manifests as a change in the total contour: a decrease in intensity in the 3040–3045 cm^−1^ range and a certain increase in the lower-frequency 2898–3029 cm^−1^ range. Due to the high anharmonicity of hydrogen stretching vibrations [33], which is not accounted for in these calculations, the corresponding experimental frequency ranges would be approximately 5–6% lower. This can be estimated from the overestimation of vibration frequencies, for example, for the methylene group, whose experimental frequencies are typically 2925 and 2870 cm^−1^ for ν_as_(CH_2_) and ν_s_(CH_2_), respectively [31].

Nevertheless, it is hypothesized that the formation of Models 2, 3, and 4 would manifest in the experimental band contour as a decrease at the high-frequency edge with a concomitant increase in the band center due to contributions from Models 2 and 3. Despite the complexity of the processes occurring, especially those involving hydrogen bonds, a general tendency towards a decrease in the IR absorption and Raman bands in the region of C-H stretching vibrations was noted in [15]. Moreover, both [15] and [30] noted a change in the profile in the range of 2800–3000 cm^−1^. Thus, instead of two distinct peaks, ref. [30] demonstrated the appearance of a single peak, but shifted to slightly lower wavenumbers. The contributions to the experimental band in the 2800–3000 cm^−1^ range, where the peaks were initially distinct, became smaller. According to the Raman difference spectrum in Figure 3b, a positive intensity can be noted for Models 2 and 3 in the center of the 2800–3000 cm^−1^ region. At the same time, negative intensity is observed at the boundary for Model 4. This coincides with the formation of the experimental band contour in the 2800–3000 cm^−1^ region for the degraded case, as reported in [15,30].

The spectral region below 1900 cm^−1^ is more often experimentally characterized for cellulose nitrate samples. In particular, Raman spectra in this range from cultural heritage objects and polymer samples are presented in [12,13,30].

In the 1600–1900 cm^−1^ region, vibrations from C=O bonds and asymmetric stretching of the NO_2_ group are observed. It has been noted [30] that as the polymer degrades, a peak appears in both IR and Raman spectra within the C=O stretching region. This peak may manifest as a shoulder initially or as a more distinct band, often with a complex contour consisting of several poorly resolved components [30]. In [30] for a 225 h irradiation, two peak positions in the Raman spectrum were mentioned: the main peak at 1754 cm^−1^ and a shoulder at 1800 cm^−1^. Moreover, for a lower radiation time (50 h), the main peak position was lower. Such a transformation of the v(C=O) peak supports the idea of possible different degradation products and the corresponding environment of the C=O bond. In our calculations for Models 2, 3, and 4, the frequency of this mode was predicted to be 1790, 1774, and 1768 cm^−1^, respectively. The corresponding atomic displacements in these modes are shown in Figure S5. The frequency decreases from Model 2 to Model 4, while concurrently, the frequency of the band maximum formed by two ν_as_(NO_2_) vibrational modes increases for each structure: 1649 and 1654 cm^−1^ for Model 2; 1650 and 1659 cm^−1^ for Model 3; 1656 and 1662 cm^−1^ for Model 4. For comparison, in Model 1, a band with a maximum at 1649 cm^−1^ was predicted, formed by three vibrational modes at 1647, 1649.5, and 1650 cm^−1^. In the present study, for the historical sample, a broad peak with a maximum at 1665 cm^−1^ was observed in this range. Its position is shifted to higher wavenumbers compared to that reported in [30]. The calculation for the NC model predicts a band maximum for ν_as_(NO_2_) at 1655 cm^−1^, which is higher in frequency than the maximum for the analogous band in Model 1 (1648 cm^−1^). At the same time, a series of relatively narrow nitrate peaks at 648, 854, and 1284 cm^−1^ is observed. This may indicate interactions through the oxygen atoms of the nitrate groups and/or the onset of degradation processes.

In the 1410–1500 cm^−1^ range, the calculations predict vibrational modes primarily involving various hydrogen displacements. Within the 1410–1500 cm^−1^ region, the greatest difference from Model 1 is observed for Model 4 around 1447 cm^−1^, associated with symmetric hydrogen bending in the methylene group connected to the NO_2_ group. In the experimental spectrum of the historical sample, a corresponding peak is observed at approximately 1454 cm^−1^.

Within the 1350–1410 cm^−1^ region, Model 1 exhibits bending vibrations δ(XCH), where X = C or O atoms, of axial hydrogens. The atomic displacements in these modes are highly delocalized across the ring hydrogens. Similar modes in Models 2, 3, and 4 show a lower degree of delocalization and reduced Raman activity, leading to lower calculated intensity in this range for these models. In [30], prolonged irradiation of a cellulose nitrate film resulted in a decrease in the relative intensity of a narrow peak at 1374 cm^−1^ and the formation of a broader band.

In the 1280–1350 cm^−1^ range, vibrational modes in Model 1 have predominantly mixed atomic displacements: the 1315–1350 cm^−1^ involves δ(CCH), δ(OCH), and w(HCH); the 1280–1315 cm^−1^ involves δ(CCH), δ(OCH), and τ(HCH). For specific modes in the 1283–1297 cm^−1^ range, symmetric stretching displacements within the NO_2_ group are additionally mixed in. The manifestation of this latter vibration type in the calculated Raman spectra of Model 1 is relatively minor, owing to the small number of such groups. In the case of the NC model, the contribution from the nitrated groups is larger than in Model 1, and the 1297 cm^−1^ band is observed, corresponding to the 1284 cm^−1^ band in the experimental spectrum (Figure 4). The most significant difference in the 1280–1350 cm^−1^ range is observed between Model 4 and Model 1 because of a lower degree of delocalization, a frequency shift to lower wavenumbers, and the absence of the methylene group attached to NO_2_.

Within the 1000–1150 cm^−1^ range, the Raman spectrum includes contributions from C–C and C–O stretching vibrations, including those in the second ring. These contributions are greater in the case of Model 1 than in the NC model (Figure 4). The presence of a ketone group in Models 2–4, replacing a C–O bond, leads to a decrease in the frequency of individual modes, which could result in spectral broadening and reduced peak resolution. The corresponding signal in the difference intensity spectrum is negative and delocalized across the 1000–1150 cm^−1^ region, as can be seen in Figure 4d.

**Figure 6:**
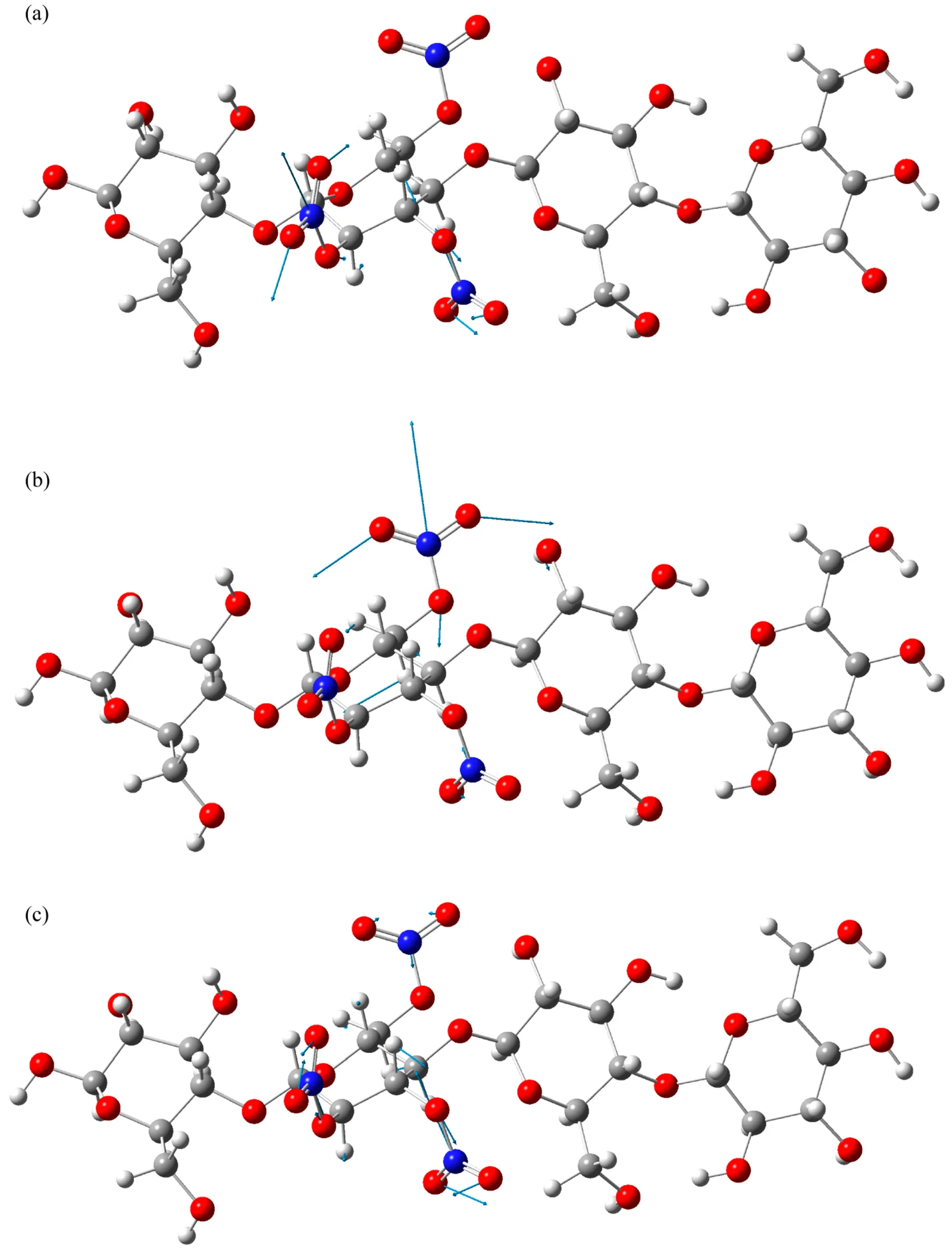
Atomic displacements in modes 111 (**a**), 112 (**b**) and 113 (**c**) for Model 1.

The most significant systematic differences between Model 1 and Models 2–4 are observed in the ranges 845–835 cm^−1^, 770–650 cm^−1^, and around 606 cm^−1^. For Models 2–4, a decrease in the frequency of vibrational modes localized in the NO_3_ group is observed. These are interpreted as involving both N–O stretching and NO_2_ bending vibrations. The corresponding band in Model 1 is formed by three vibrational modes (No. 111–113 Figure 6).

**Figure 7:**
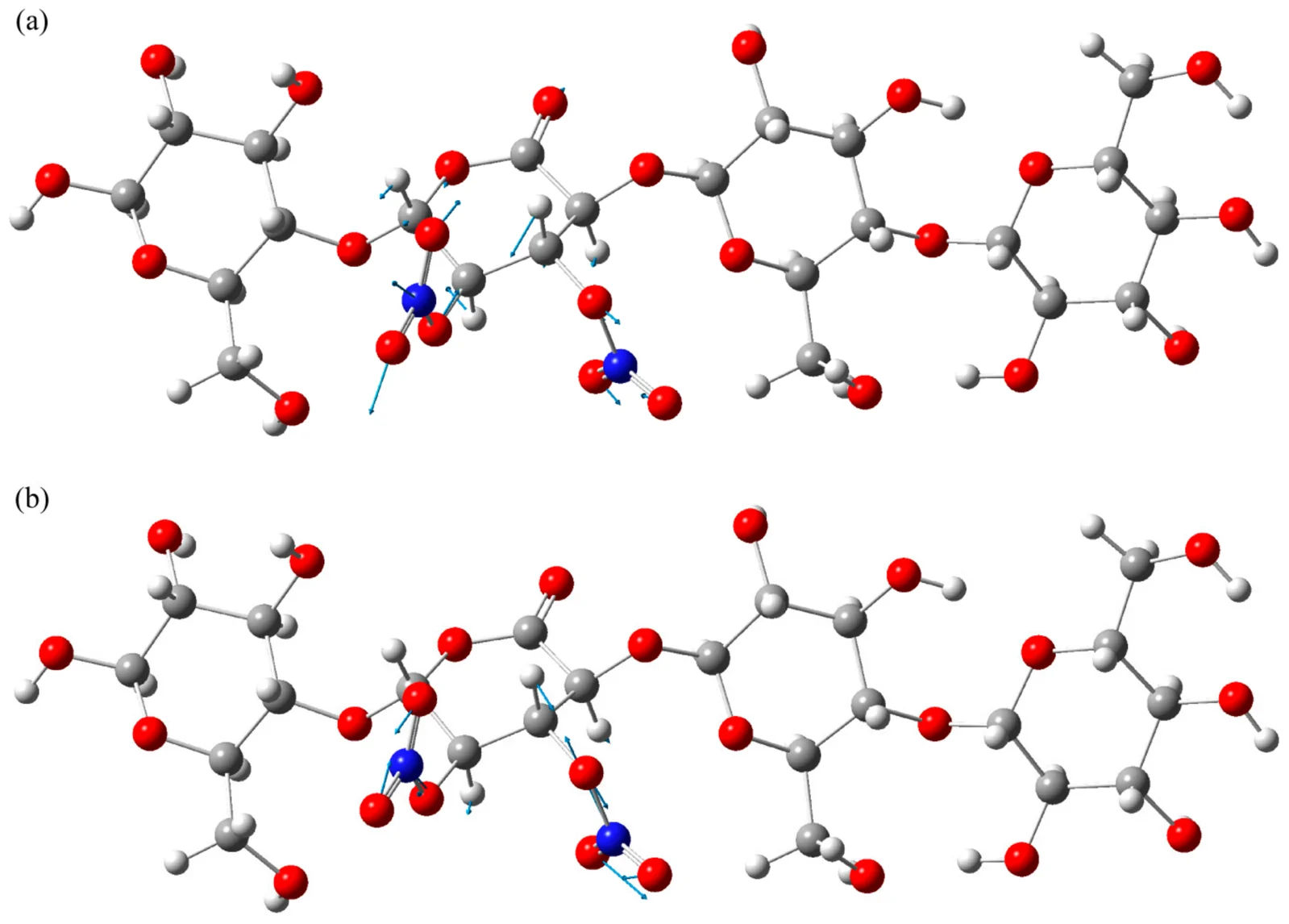
Atomic displacements in modes 103 (**a**) and 104 (**b**) for Model 4.

For the two adjacent nitrate groups, both antiphase (mode No. 111 at 834 cm^−1^) and in-phase vibrations (mode No. 113 at 847 cm^−1^) are observed, as well as vibrations localized only in the methyl nitrate part (mode No. 112 at 840 cm^−1^). Mode No. 113 has the highest intensity. In Models 2 and 3, only one vibrational mode with a lower frequency is present due to the absence of coupling with the conjugated nitrate group. This also leads to the absence of an intense in-phase vibrational mode for adjacent nitrate groups. For Model 4, the corresponding modes are No. 103 (830 cm^−1^) and 104 (839 cm^−1^) (Figure 7).

Their atomic displacements are more delocalized, including pyranose ring bonds, which lowers their frequencies. Overall, the band maximum formed by these modes shows a decreasing frequency in the series: Model 1 (845 cm^−1^), Model 2 (841 cm^−1^), Model 3 (839 cm^−1^), Model 4 (837 cm^−1^).

In the 770–650 cm^−1^ range and near 606 cm^−1^, differences are associated with vibrational modes delocalized in the pyranose ring and in N–O bonds at the boundary with the nitrate group. This leads, for instance, to the modification of vibrational mode No. 95 (605 cm^−1^) in Model 1 (which includes contributions from stretching in all three N–O bonds of the nitrate groups) upon moving to Models 2–4. The single intense band near 606 cm^−1^ in Model 1 is replaced by a set of weaker modes in Models 2–4, producing positively defined areas in the 650–770 cm^−1^ region of the difference spectra.

In the 200–525 cm^−1^ region, significant differences between the spectrum of Model 1 and those of Models 2–4 may occur. This is related to differences in the reduced masses of the CON and ONO groups compared to the CC=O ketone group formed upon nitrate removal.

Below 200 cm^−1^, contributions from torsional modes and non-hydrogen bending vibrations could significantly influence the interpretation of results obtained with the cluster model approach, warranting further study.

## 3. Materials and Methods

### 3.1. Theoretical Approach

To investigate structure-spectrum correlations, five model clusters consisting of four pyranose rings with varying degrees of nitration were constructed. For Models 2–4, the removal of a nitrate group with the formation of a ketone group can be considered a relatively small perturbation, which may correspond to degradation products at the initial stage. Hydroxyl groups were employed as terminal groups, replacing the corresponding glycosidic bonds. This combination of cluster size and terminal group selection ensures a negligible influence from the chain termini on the core vibrational properties. Additionally, we verified the influence of chain length on the predicted vibrational properties. Cluster models with lengths ranging from 2 to 5 pyranose rings were considered. Additionally, the influence of the interchain interaction on vibrational properties was tested. The corresponding results are presented in the Supplementary Materials (Figures S6–S12 and the accompanying text).

The NC (nitrocellulose) model represents a structure with maximum substitution, where all hydroxyl groups are replaced by nitrate groups, corresponding to a degree of substitution parameter of γ = 3. Industrially produced cellulose nitrate, however, typically has a degree of substitution in the range of γ = 2.0–2.7 [23], indicating greater chemical inhomogeneity. To isolate spectral contributions, Model Structure 1 (hereafter Model 1) was chosen. Its characteristic feature is a degree of substitution γ = 3 localized within a single pyranose ring, while the other three rings remain unsubstituted. This design allows for the identification of spectral ranges in Raman and IR absorption spectra containing peaks from nitrate groups adjacent to hydroxyl groups, as well as peaks from other bonds in unsubstituted regions. Crucially, each type of nitrate group attachment in Model 1 occurs only once, enabling targeted study of specific nitrate substitution effects. Unlike the NC model with many nitrate groups, Model 1 exhibits significantly less spectral overlap from other nitrate vibrational modes. Therefore, Models 2, 3, and 4 were derived from Model 1 by selectively replacing one nitrate group with a ketone group. For comparison, ketone structures (Models 2–4) were compared with the gluconolactone case (Model 5) and the anhydride case (Model 6). Models 5 and 6 were designed on the basis of the results reported in [28,31]. Their optimized geometries and vibrational properties are presented in Tables S11–S14. To address the lack of explicit inter-chain interactions, the clusters were placed within a continuum solvation model with parameters for water. Furthermore, a preliminary assessment of the influence of the solvent dielectric constant on the results was conducted by comparing calculations in water and methanol. The results are presented in the Supplementary Materials (Figures S20–S21 and the accompanying text).

Quantum-chemical calculations of the model structures were performed within the density functional theory (DFT) framework using Gaussian 09W Rev. C.01 [34]. The B3LYP exchange-correlation functional [35,36,37] was combined with the 6-311G(2d,p) triple-zeta basis set [38,39], a combination that has been previously shown to provide accurate predictions of structural and vibrational properties [40]. To account for the interaction of the cluster with a surrounding medium, the integral equation formalism of the polarizable continuum model (IEF-PCM) [34,41] was employed using standard parameters for water at 25 °C and 1 atm [34]. An additional rationale for using the water-based PCM model is the observed broadband contribution from O–H stretching vibrations in several experimental IR absorption spectra of cellulose nitrate [23]. Accounting for solvent effects within the PCM model leads to a downshift in the frequencies of vibrational modes involving highly polar bonds, which is particularly important for accurate modeling of NO_2_ groups.

The geometry of each model was optimized using standard convergence criteria for maximum forces, root-mean-square forces, and atomic displacements. The optimized Cartesian coordinates are provided in the Supporting Information (Tables S1–S5). The absence of imaginary frequencies in the subsequent vibrational frequency calculations confirmed that each optimized geometry corresponds to a minimum on the potential energy surface (a stable equilibrium structure). To improve agreement with experimental frequencies, a scaling factor of 0.983, as reported in [33], was applied to all calculated harmonic frequencies.

Spectral contours for both Raman and IR absorption were generated by assigning a Lorentzian line shape with a half-width at half-maximum (HWHM) of 10 cm^−1^ to each vibrational mode. The Raman intensity *I*(*ν_i_*) for the i-th vibrational mode with frequency *ν_i_* was calculated from the computed Raman activity *R_a_* for monochromatic 785 nm excitation (source frequency *ν*_0_), at T = 298.15 K under non-resonant conditions, following the approach in [42] using Equation (1):

(1) $$I(v_{i})\;=\;\frac{C{\left(v_{0}-v_{i}\right)}^{4}R_{a}}{v_{i}\left(1-e^{\left(-\frac{hcv_{i}}{k_{B}T}\right)}\right)}$$

where *h*—Planck constant, *k_B_*—Boltzmann constant, *c* is the speed of light, and *C* is the constant identical for all models.

### 3.2. Sample Preparation

Microprobes of the cellulose nitrates were taken from the well preserved test regions of the historical photography previously studied in [5,6].

### 3.3. Raman Spectroscopy

Raman spectra were measured for the cellulose nitrate samples using a portable Bravo (Bruker Corporation, Billerica, MA, USA) Raman spectrometer with DuoLaser™ technology [43] in backscattering geometry using the sequentially shifted excitation (SSE) [44] technique. The resolution was about 10 cm^−1^ in the 300–1800 cm^−1^ range. The 100 mW laser power was focused on the 1 mm diameter spot on the sample. The acquisition time was 3 s with 10 repetitions. Baseline correction of the residual luminescence contribution was performed on the obtained spectra, followed by normalization to the maximum, using OriginPro2021b (OriginLab Co.; Northampton, MA, USA) software.

### 3.4. FTIR Spectroscopy

The FTIR absorbance spectra of the samples were obtained using a Nicolet6700 (Thermo Scientific, Waltham, MA, USA) spectrometer with an attenuated total reflection (ATR) accessory and an XT-KBr beam splitter. The ATR crystal was diamond. The detector was mercury cadmium telluride (MCT-A) with liquid nitrogen cooling. The spectral resolution was 8 cm^−1^. The spectra were recorded in the 650–4000 cm^−1^ region. The Blackman–Harris apodization function was used. The phase correction was made according to the Mertz technique. Normalization to the maximum was performed using OriginPro2021b (OriginLab Co.; Northampton, MA, USA) software.

## 4. Conclusions

In this study, the structural and vibrational properties of model structures were investigated to simulate the photodegradation process of cellulose nitrate. The models consist of four pyranose rings, which are either fully substituted or entirely unsubstituted. Two primary reference structures were examined: one with the maximum degree of nitration (γ = 3) in all four rings, and another with γ = 3 in only one ring, while the other three remained unsubstituted (γ = 0). This approach enabled the identification of spectral peaks associated with the NO_3_ moiety at different substitution positions. In the IR absorption spectrum, these characteristic peaks appear at 1655, 1289, 1024, 830, and 760 cm^−1^. In the Raman spectrum, the most intense peaks are observed at 605 and 851 cm^−1^. Concurrently, the structure containing three unsubstituted rings clearly demonstrated the spectral contributions from hydroxyl-substituted pyranose rings.

To elucidate the spectral signatures of photodegradation products, structures featuring the replacement of a nitrate group with a ketone group in different positions were examined. As expected, the IR absorption peaks at 1655, 1289, 1024, 830, and 760 cm^−1^ and the Raman peaks at 605 and 851 cm^−1^ all exhibited decreased intensity in the spectra of the photodegraded model structures. This reduction is consistent with experimental observations from prior studies. Furthermore, sensitivity to the specific position of nitrate group abstraction was noted in the vibrational spectra. This sensitivity is reflected in the frequency shifts of bands within the ranges 1760–1790 cm^−1^ (C=O stretch), 1647–1660 cm^−1^ (NO_2_ asymmetric stretch), and 838–830 cm^−1^ in both IR and Raman spectra. Analysis of the C–H stretching vibrations based on the present calculations allowed us to explain the observed changes in the spectral contour in the 2800–3000 cm^−1^ range. These changes are associated with the depletion of certain axial hydrogens and the corresponding vibrational modes, as well as with the appearance of C=O bonds during degradation at the initial stages. The discovered theoretical relationships between frequencies and intensities can be further used to gain a deeper understanding of the obtained spectral data and analyze the processes taking place. Specifically, the obtained results enable peak-based deconvolution and peak attribution in complex contours.

## Figures and Tables

**Table 1:** Selected bond lengths of studied models in comparison with experimental values from [24].

Structural Property	Theoretical Model					Experimental Value [24]
	NC-Model	Model 1	Model 2	Model 3	Model 4	
Bond, Å						
C14C10	1.538	1.538	1.541	1.538	1.524	1.523
C10C6	1.532	1.530	1.532	1.532	1.526	1.521
C6C2	1.536	1.538	1.538	1.541	1.534	1.522
C2O34	1.424	1.424	1.429	1.422	1.457	1.430
O34C18	1.427	1.424	1.424	1.422	1.347	1.436
C18C14	1.541	1.542	1.541	1.545	1.537	1.525
C10O30	1.449	1.452	1.434	--	1.446	1.429
C6O28	1.444	1.450	--	1.433	1.442	1.423
C18C22	1.528	1.528	1.528	1.528	--	1.513
C22O36	1.439	1.441	1.441	1.441	--	1.440
C14O32	1.432	1.426	1.426	1.413	1.412	1.426
C2O31	1.386	1.383	1.374	1.382	1.377	1.389
C=O *	--	--	1.200	1.202	1.200	--
O28N83	1.417	1.416	--	1.422	1.426	1.372
N83O84	1.209	1.208	--	1.206	1.207	1.220
N83O85	1.196	1.198	--	1.198	1.196	1.198
O30N86	1.413	1.411	1.416	--	1.419	1.371
N86O88	1.198	1.199	1.200	--	1.197	1.220
N86O87	1.208	1.208	1.206	--	1.208	1.220
O36N89	1.420	1.412	1.411	1.411	--	1.371
N89O90	1.200	1.204	1.204	1.204	--	1.220
N89O91	1.205	1.204	1.204	1.204	--	1.220

* in case of model 2,3,4 it is C6, C10 and C18 atoms and corresponding oxygens.

**Table 2:** Selected flat angles of studied models in comparison with experimental values from [24].

Structural Property	Theoretical Model					Experimental Value [24]
	NC-Model	Model 1	Model 2	Model 3	Model 4	
Angle, ^o^						
C14C10C6	113.2	113.5	115.8	115.6	109.6	110.3
C10C6C2	110.3	111.0	112.6	112.8	111.3	110.5
C6C2O34	105.8	105.7	106.3	105.5	110.4	109.4
C2O34C18	113.9	113.6	113.4	113.8	125.6	112.3
O34C18C14	108.5	109.0	108.9	108.9	119.2	110.1
C18C14C10	108.5	108.6	108.7	110.5	112.1	110.2
O32C14C10	106.3	106.5	106.8	108.8	108.4	110.5
O30C10C6	113.3	113.3	112.6	121.7	114.5	109.7
O28C6C2	116.1	115.0	124.5	115.5	112.9	109.3
O31C2O34	108.3	108.0	108.7	108.1	106.9	107.3
O34C18C22	103.6	102.9	103.0	102.9	119.5	106.9
O36C22C18	113.3	114.2	114.1	114.2	--	111.8
N86O30C10	118.4	118.5	116.2	--	118.6	117.5
O87N86O30	118.8	118.9	118.6	--	118.6	118.0
O88N86O87	129.2	129.0	129.4	--	129.4	124.0
C6O28N83	118.4	118.5	--	116.2	118.4	117.6
O28N83O84	118.5	118.7	--	118.4	118.5	118.0
O85N83O84	129.3	129.2	--	129.6	129.7	124.0
C22O36N89	114.6	115.1	115.0	115.0	--	112.5
O36N89O91	118.2	118.5	118.5	118.5	--	118.0
O90N89O91	129.5	129.2	129.2	129.2	--	124.0

## Data Availability

Data is contained within the article or Supplementary Materials.

## Supplementary Materials

The following supporting information can be downloaded at: https://www.mdpi.com/article/10.3390/molecules31162890/s1, Table S1: Optimized geometry for the NC model; Table S2: Optimized geometry for the Model 1; Table S3: Optimized geometry for the Model 2; Table S4: Optimized geometry for the Model 3; Table S5: Optimized geometry for the Model 4; Table S6: Calculated vibrational modes frequencies and activities for the NC model; Table S7: Calculated vibrational modes frequencies and activities for the Model 1; Table S8: Calculated vibrational modes frequencies and activities for the Model 2; Table S9: Calculated vibrational modes frequencies and activities for the Model 3; Table S10: Calculated vibrational modes frequencies and activities for the Model 4; Table S11: Optimized geometry for the model 5; Table S12: Calculated vibrational modes frequencies and activities for the Model 5; Table S13: Optimized geometry for the model 6; Table S14. Calculated vibrational modes frequencies and activities for Model 6. Figure S1: Atomic displacements in mode No. 149 in NC model (for notations in second from the left side pyranose ring see Figure 1b); Figure S2: Calculated scaled infrared (IR) absorption spectra for: (a) the NC model; (b) model structures 1 (black), 2 (red), 3 (green), and 4 (blue); (c) difference scaled IR absorption spectra, obtained by sequentially subtracting the scaled IR absorption spectrum of model 1 from the scaled spectra of model 2 (red), model 3 (green), and model 4 (blue) in the selected regions. The position of peak maximum for each model is highlighted with the same color; Figure S3: Atomic displacements in mode No. 113 in Model 4 (for notations in second from the left side pyranose ring see Figure 1b); Figure S4: Atomic displacements in mode No. 147 in Model 4 (for notations in second from the left side pyranose ring see Figure 1b); Figure S5: Atomic displacements in modes with predominant v(C=O) atomic displacements in Model 2 (a), Model 3 (c) and Model 4 (d); Figure S6: Calculated IR absorbance spectra with variable number of pyranose rings in the model structure: 2 (black), 3 (red), 4 (blue) and 5 (pink) normalized by 830 cm^−1^ peak area; Figure S7: Structure of the models with 2 (a), 3 (b), 4 (c) and 5 (d). Red, blue, light grey and dark grey colors correspond to the oxygen, nitrogen, hydrogen and carbon; Figure S8. Calculated Raman activity with variable number of pyranose rings in the model structure: 2 (black), 3 (red), 4 (blue) and 5 (pink) normalized by 844 cm^−1^ peak area; Figure S9: The optimized geometries of the 2 units structure (a) and 2 + 2 = 4 units structure (b); Figure S10: a—Optimized structure of 2 pyranose ring units; b -; c—calculated IR absorbance spectra with 2 pyranose ring units (black solids) and 2 + 2 = 4 pyranose ring units (pink dashed); Figure S11. a—Optimized structure of 2 pyranose ring units; b—calculated scaled IR absorbance spectra with 2 pyranose ring units (black solids) and 2 + 2 = 4 pyranose ring units (pink dashed); Figure S12. Hydrogen bending vibrational mode with scaled frequency 1343 cm^−1^ (a) and hydrogen-carbon stretching vibrational mode with scaled frequency 3055 cm^−1^ (b); Figure S13: Optimized geometries of the model 5(a) and model 6(b); Figure S14: Calculated scaled infrared (IR) absorption spectra for the model 5(a) and model 6(b). The theoretical approach is B3LYP/6-311G(2d,p) with water parameters PCM solvation model. The scaling factor is 0.983; Figure S15: Antisymmetric atomic displacements in modes 174 (a), 175 (b) and 176 (c) for model 5 as well as antisymmetric atomic displacements in modes 156 (d) and 157 (e) for model 6; Figure S16: Amplitudes of atomic displacements in modes 86 (a),87 (b) and 88 (c) for model 5 and in modes 78 (d) and 79 (e) for model 6; Figure S17: Atomic displacements in mode 128(a) of model 5 and mode 116 (b) of model 6; Figure S18: Calculated scaled Raman spectra for model structures 5 (a) and 6 (b). The theoretical approach is B3LYP/6-311G(2d,p) with water parameters PCM solvation model. The scaling factor is 0.983; Figure S19. Atomic displacements in vibrational modes 65 (a), 69 (b), 80 (c) for model 5 as well atomic displacements in vibrational mode 61 (d) for model 6; Figure S20: a—structure image of the optimized geometries of the NC model in water, b—structure image of the optimized geometries of the NC model in methanol solvent taken from the same view as in case (a), c—the scaled calculated IR absorbance spectra of NC model in water (red dashed line) and methanol (blue solid line) solutions, d—the enlarged part of the (c) spectra; Figure S21: Example of optimized structural model 2 (a) and corresponding IR absorbance scaled spectra (b), structural model 3 (c) and corresponding IR absorbance scaled spectra (d), structural model 4 (a) and corresponding IR absorbance scaled spectra (f) in water (black), methanol (red) and o-Cresol (blue) solvents. References [19,30,34] are cited in the Supplementary Materials.
