# Supplementary material for: Vibrational Spectra Modeling of Cellulose Nitrate During Initial Photodegradation Stage by Density Functional Theory: The Case of Ketone Formation
Source: Molecules. 2026 Aug 19;31(16):2890. doi: 10.3390/molecules31162890 (PMC13515503; doi:10.3390/molecules31162890)
Supplement: Supplementary file 1 [file molecules-31-02890-s001.zip › molecules-4237145-supplementary.pdf]

Supporting information

for

Vibrational Spectra Modeling of Cellulose Nitrate During Initial Photodegradation Stage by Density Functional Theory: The Case of Ketone Formation

Dmitrii Pankin[1], Maxim Moskovskiy[2], Anastasia Povolotckaia[1,2*]

[1] Center for Optical and Laser Materials Research, Saint-Petersburg State University, Universitetskaya nab. 7/9, Saint-Petersburg 199034, Russia;
dmitrii.pankin@spbu.ru
[2] Federal Scientific Agroengineering Center VIM, Moscow 109428, Russia; maxmoskovsky74@yandex.ru
* Correspondence: anastasia.povolotckaia@spbu.ru

Table S1. Optimized geometry for the NC Model.

C	-0.60301400	-1.14043500	0.47818700
C	4.42381200	0.66655200	0.46117000
H	-0.44420000	-1.99139400	-0.19006700
H	4.65926700	0.41753800	1.49816000
C	-1.65723700	-1.41251900	1.56342700
C	3.28960300	1.69567400	0.33829400
H	-1.63059100	-0.54144500	2.21545100
H	3.18744100	1.90069600	-0.72520700
C	-3.06145100	-1.57025700	0.95838600
C	1.98664000	1.12126200	0.90424800
H	-3.11603700	-2.49876600	0.39137600
H	2.08514800	0.99910700	1.98268500
C	-3.41673900	-0.43948100	-0.01960700
C	1.63551400	-0.26199500	0.33036200
H	-3.53441600	0.51002800	0.50045100
H	1.32834800	-0.17953500	-0.71001200
C	-2.25607800	-0.32105500	-1.02194700
C	2.88446100	-1.16014600	0.42305900
H	-2.13907600	-1.26848600	-1.55923200
H	3.12137000	-1.35195000	1.47589100
C	-2.40810400	0.80310500	-2.04820600
C	2.77719200	-2.48689900	-0.32651300
H	-2.96777100	1.65265700	-1.66151400
H	2.40571400	-2.33847000	-1.33972200
H	-1.42217800	1.13879600	-2.35988200
H	3.75917000	-2.95139700	-0.36696400
O	-1.33084100	-2.45168800	2.50995000
O	3.58045500	3.02291400	0.82719900
O	6.53197800	-1.71391900	0.54627700
O	0.88646600	2.06432000	0.87972300
O	5.52911300	1.16507200	-0.21071000
O	0.56806200	-0.77281900	1.13678500
O	-1.07270900	-0.03031600	-0.27843400

O	3.96975300	-0.49585300	-0.22375800
O	-3.17432500	0.39280500	-3.19608300
O	1.82330100	-3.39358700	0.25484600
O	-4.06465400	-1.85496400	1.96195100
O	-4.61941600	-0.79800900	-0.70243900
C	7.15330500	-0.67512700	-0.26516500
C	6.83072600	0.75619800	0.22383600
C	-5.69616900	0.07033900	-0.56154900
C	8.63668300	-0.97132800	-0.51065800
H	6.60184900	-0.81429100	-1.19279700
H	6.88974800	0.79929100	1.31184500
C	7.79850900	1.78880000	-0.38984200
H	-5.40453600	1.10876000	-0.73880800
O	-6.21646600	-0.04445400	0.75769300
C	-6.79894600	-0.41382900	-1.51579700
H	9.15737900	-1.11514700	0.43349400
O	8.84826400	-2.27047900	-1.11708200
C	9.35799800	0.19736200	-1.18640700
O	9.13678200	1.31789400	-0.33110400
C	7.85129600	3.12302000	0.35663400
H	7.49950400	1.96014400	-1.42971700
C	-7.28937100	0.86820500	1.00678300
C	-8.09567500	0.37421700	-1.30083900
O	-6.42980400	-0.51401300	-2.90953100
H	-6.95130400	-1.46502000	-1.27973000
H	8.96976100	0.42861300	-2.18092100
H	8.03895700	2.96490100	1.41793000
H	8.64909900	3.73083000	-0.06363700
O	6.61363500	3.85589900	0.34949000
H	-6.99245600	1.88875700	0.73883900
C	-7.52610800	0.77626100	2.51296500
C	-8.51788900	0.46490600	0.16759600
H	-7.96364500	1.39878800	-1.64872200
O	-9.16106000	-0.03608700	-2.19715900
O	-8.64128300	1.58050000	2.94794400
H	-6.62877500	1.05492900	3.05883800
H	-7.82094500	-0.23437000	2.79327200
O	-9.53884900	1.44627000	0.21529600
H	-8.87874400	-0.50364200	0.52003500
O	10.70594700	-0.10090600	-1.20661400
N	3.88964200	3.18267800	2.20099000
O	3.93186100	2.19779700	2.90089400
O	4.07728300	4.32792600	2.49163700
N	0.38982700	2.48451200	-0.37501700
O	0.94387400	2.10105200	-1.37774800
O	-0.56166500	3.20472800	-0.26420400
N	2.33359500	-4.18778000	1.31553900
O	1.51400100	-4.95105300	1.74756100
O	3.47911700	-4.02290100	1.64956100
H	11.14987200	0.50533700	-1.81286500
H	-9.94426800	1.42015400	1.09059000
N	8.42406300	-2.45986800	-2.45596900
O	7.88287200	-1.54212500	-3.02604300

O	8.67309100	-3.56090900	-2.85591600
N	6.99480400	-1.89158800	1.87859400
O	7.83598200	-1.13635300	2.30550100
O	6.45244500	-2.80910700	2.42339300
N	6.37077700	4.58891900	-0.83512800
O	5.34395900	5.20827900	-0.79184600
O	7.18659000	4.52243200	-1.72287300
N	-1.31840100	-3.80129000	2.04941500
O	-1.41500600	-4.00253700	0.86406100
O	-1.19908800	-4.57763000	2.95060800
N	-4.35270600	-0.86143800	2.92536200
O	-3.76352100	0.19110800	2.86988500
O	-5.18912200	-1.22136700	3.70505000
N	-2.42558500	-0.31297000	-4.17914400
O	-3.09317200	-0.62467300	-5.12433600
O	-1.25584500	-0.51162300	-3.96397400
N	-9.68944500	-1.34545900	-2.06667200
O	-9.21317500	-2.08542200	-1.23948600
O	-10.58170300	-1.54675100	-2.84036200
N	-6.14218300	0.68657400	-3.61533500
O	-6.04865900	1.71377800	-2.98609500
O	-6.01758500	0.49906200	-4.78891400
N	-8.33069100	2.95137600	3.19183000
O	-7.18865200	3.30545000	3.05170300
O	-9.29614600	3.58000300	3.52260000

Table S2. Optimized geometry for Model 1.

C	1.23002600	0.58955100	0.11288700
C	-4.02897300	-0.27544300	0.17511200
H	1.13760800	1.07600300	-0.86845400
H	-4.24044300	0.31242600	1.06816600
C	2.23518300	1.33278000	0.99595400
C	-3.12210900	-1.48730200	0.44834300
H	2.26276300	0.81789500	1.95967400
H	-3.04748600	-2.02551800	-0.49422000
C	3.62330700	1.26502100	0.35515800
C	-1.73979600	-1.03974600	0.92840100
H	3.61056000	1.86926500	-0.56490600
H	-1.82520600	-0.61033300	1.92578200
C	3.98854900	-0.17099700	-0.04324700
C	-1.10152900	0.04025200	0.03917000
H	4.17199200	-0.75132400	0.86721300
H	-0.77067800	-0.38895200	-0.90521000
C	2.86743100	-0.84086800	-0.84431000
C	-2.15283300	1.13423800	-0.23816500
H	2.74439000	-0.31450400	-1.80201000
H	-2.37793200	1.66568500	0.69405900
C	3.07874600	-2.33171900	-1.12583200
C	-1.76923700	2.13866500	-1.32430800
H	3.42222600	-2.84167600	-0.22549600
H	-1.48074000	1.62504000	-2.24040400
H	2.11519200	-2.75944100	-1.41369300

H	-2.61801600	2.78674500	-1.52688300
O	1.83222700	2.65826200	1.28142900
O	-3.71024000	-2.51853300	1.28120000
O	-5.66426600	2.18976200	0.16714200
O	-0.85241300	-2.14724500	1.23367600
O	-5.18539700	-0.73160300	-0.43052000
O	0.00232900	0.56735100	0.77254200
O	1.66025700	-0.75398900	-0.08363800
O	-3.32570800	0.52609600	-0.76900600
O	4.05805000	-2.59752600	-2.12855300
O	-0.60876500	2.93321400	-1.00952800
H	1.77773300	3.16528800	0.46047500
H	-6.03412100	2.35943100	1.04313800
O	4.55661600	1.79574200	1.27917300
O	5.16496600	-0.17323600	-0.86087500
C	-6.55172800	1.32780100	-0.53874700
C	-6.43522300	-0.12945500	-0.06740600
H	3.76980900	-2.20511900	-2.96164800
H	5.43927600	1.73234500	0.87520000
C	6.39447600	-0.37665400	-0.24853900
C	-7.99687100	1.84428700	-0.46735600
H	-6.21488300	1.36428800	-1.57979200
H	-6.55308200	-0.16356300	1.02220000
C	-7.51758500	-1.01200600	-0.70314100
H	6.27984400	-0.94299500	0.68521600
O	6.95721000	0.90981300	0.04486500
C	7.27721600	-1.15930600	-1.22627100
H	-8.29065200	1.95788400	0.58133100
O	-8.12298100	3.13561600	-1.03757000
C	-8.96823000	0.83347100	-1.07670000
O	-8.79127700	-0.43019200	-0.44035500
C	-7.56616300	-2.44948900	-0.16355000
H	-7.34179000	-1.05850700	-1.78787500
C	8.23800300	0.83087800	0.68536700
C	8.69892000	-1.23866900	-0.68226100
O	6.77605700	-2.46749100	-1.39352200
H	7.27758900	-0.60415500	-2.17616300
H	-7.80223900	3.10408500	-1.94773100
H	-8.77824100	0.71457800	-2.15515600
H	-7.26478300	-2.47336000	0.88499200
H	-8.60795500	-2.77969200	-0.22494100
O	-6.69847300	-3.34336300	-0.84479600
H	8.16372300	0.24477700	1.61070000
C	8.65952800	2.25590800	1.03870900
C	9.20569400	0.14401300	-0.27909800
H	8.69783100	-1.88001500	0.20403900
O	9.62346100	-1.85712500	-1.57421200
H	5.85264800	-2.41037300	-1.70905300
H	-7.05249600	-3.48474700	-1.73028600
O	9.81948900	2.27622600	1.85630700
H	7.85965300	2.73259600	1.60801100
H	8.80402100	2.82820400	0.11319400
O	10.47992100	0.03618400	0.35199000

H	9.28571400	0.77019600	-1.17904700
H	9.57120300	-1.40806900	-2.42825900
H	10.47321000	1.70498900	1.42579400
O	-10.26538300	1.26357400	-0.83407900
N	-4.06961700	-2.19089800	2.61088800
O	-3.85394000	-1.07157600	3.01181200
O	-4.56532400	-3.11879100	3.18481900
N	-0.41324400	-2.96885100	0.17357700
O	-0.83510100	-2.76114600	-0.93938700
O	0.35563900	-3.81090500	0.54511100
N	-0.86423200	4.08224800	-0.23056000
O	0.13464600	4.72015000	-0.01816500
O	-1.99588300	4.30112000	0.11690700
H	-10.86728000	0.71945400	-1.35696600
H	11.03414000	-0.50070500	-0.23150100

Table S3. Optimized geometry for Model 2.

C	-0.98544400	0.56259600	-0.17238300
C	4.25282000	-0.46779300	-0.52564000
H	-0.86854900	0.93188600	0.85665200
H	4.42557200	0.24525400	-1.34153200
C	-1.99681900	1.41162700	-0.94514200
H	-2.04267800	1.01507700	-1.96257400
C	-3.37734200	1.27781100	-0.29772000
C	1.88173600	-1.05977800	-1.26571700
H	-3.34963200	1.77160400	0.68557500
H	1.94267900	-0.51487600	-2.21480300
C	-3.74822800	-0.19277200	-0.06271800
C	1.32617600	-0.06088500	-0.23289900
H	-3.94683800	-0.66413000	-1.03108300
H	0.99822400	-0.59237600	0.65849800
C	-2.62099300	-0.95524200	0.64121700
C	2.44279800	0.93115100	0.14633000
H	-2.47981600	-0.54035400	1.64993300
H	2.67596400	1.56770200	-0.71517300
C	-2.83466500	-2.46798300	0.75269600
C	2.14535500	1.80499300	1.36461100
H	-3.18026900	-2.87083000	-0.19972800
H	1.89390000	1.18867000	2.22676100
H	-1.87051800	-2.92644500	0.98714900
H	3.01929600	2.40903000	1.59442300
O	-1.58542000	2.75874200	-1.07830200
O	5.85637000	1.97736300	-0.18577600
O	0.97211200	-2.10820300	-1.62495200
O	5.42360100	-1.01280600	-0.05743700
O	0.23065700	0.60713300	-0.85450400
O	-1.42534000	-0.79032900	-0.12410600
O	3.59234700	0.19676000	0.55337300
O	-3.81119600	-2.84711500	1.72055600
O	0.98760900	2.65010600	1.21344000
H	-1.48852800	3.15694400	-0.20304800
H	6.19392700	2.30458300	-1.02953200

O	-4.31752400	1.91686200	-1.14286200
O	-4.91466300	-0.28208400	0.76432800
C	6.78036000	1.02256800	0.32881400
C	6.65179100	-0.33850100	-0.36996600
H	-3.51661900	-2.56119800	2.59370200
H	-5.19636700	1.81046600	-0.73957800
C	-6.15303500	-0.40747800	0.14870800
C	8.21730500	1.56411800	0.27462600
H	6.49332700	0.88368900	1.37616700
H	6.71563200	-0.19293500	-1.45501300
C	7.76809500	-1.29583700	0.06338800
H	-6.05432000	-0.86558000	-0.84437400
O	-6.70942500	0.90742900	0.00947000
C	-7.02967200	-1.28966200	1.04353600
H	8.45143900	1.86286300	-0.75248400
O	8.36616500	2.73837300	1.05402400
C	9.23103400	0.48190700	0.64608600
O	9.02326400	-0.66421800	-0.17543900
C	7.77372300	-2.63809300	-0.68696400
H	7.65246500	-1.50861700	1.13553400
C	-7.99904300	0.90972000	-0.61829100
C	-8.45935900	-1.29634000	0.51548400
O	-6.53907000	-2.61243100	1.05354600
H	-7.01206800	-0.84624000	2.05029700
H	8.11059200	2.53902200	1.96363900
H	9.11192500	0.18621300	1.70049500
H	7.25240200	-2.54648900	-1.64043200
H	8.81845500	-2.89063700	-0.89200500
O	7.11732900	-3.68120600	0.02009000
H	-7.94202500	0.42978600	-1.60391100
C	-8.41175000	2.36846200	-0.80511200
C	-8.95960000	0.12645500	0.27723100
H	-8.47642100	-1.83406900	-0.43704100
O	-9.37624400	-2.00422900	1.34689800
H	-5.60921800	-2.59895500	1.35456400
H	7.67770300	-3.93547600	0.76224000
O	-9.58424200	2.48907700	-1.59571900
H	-7.61595800	2.89823000	-1.33166800
H	-8.53550600	2.83642800	0.18012700
O	-10.24343700	0.09877400	-0.34303700
H	-9.02172800	0.64852000	1.24243800
H	-9.30427600	-1.65778400	2.24626700
H	-10.23648300	1.87815200	-1.22108400
O	10.50647900	0.96976600	0.39632500
N	0.70025200	-3.07081000	-0.62241600
O	1.20781400	-2.93325900	0.46250900
O	-0.04459700	-3.92713400	-1.01137500
N	1.22186100	3.87019100	0.54401800
O	0.22683900	4.54311700	0.46042100
O	2.33578300	4.10629900	0.15317500
H	11.14554400	0.35572000	0.77868500
H	-10.79406500	-0.49539800	0.18579000
C	3.29857200	-1.57684100	-0.99915700

O	3.62293200	-2.71658100	-1.18854300

Table S4. Optimized geometry for Model 3.

C	1.19142700	0.46794300	0.04599600
C	-4.05161400	-0.50328300	0.13317500
H	1.11515900	0.95422200	-0.93819300
H	-4.25519200	0.06239200	1.04109300
C	2.19307100	1.20364200	0.93835900
C	-3.11586600	-1.70340600	0.37523900
H	2.20404100	0.69230800	1.90423000
H	-3.06704400	-2.23213800	-0.58110800
C	3.58667200	1.11805200	0.31171300
H	3.58819200	1.71421200	-0.61370800
C	3.94022800	-0.32535200	-0.06999300
C	-1.12583400	-0.08618700	-0.03793300
H	4.10742300	-0.89908300	0.84778200
H	-0.79821600	-0.48361500	-1.00557000
C	2.82107700	-0.98977000	-0.87832200
C	-2.22605400	0.96932500	-0.28584200
H	2.72210700	-0.47556400	-1.84556000
H	-2.45680600	1.47607500	0.65733400
C	3.01282800	-2.48785000	-1.13259700
C	-1.89508000	2.00077600	-1.36393700
H	3.33647200	-2.98785700	-0.21921000
H	-1.64626300	1.50525000	-2.30161200
H	2.04388700	-2.90218700	-1.42417500
H	-2.75288400	2.65112900	-1.51560100
O	1.80112100	2.53463600	1.21434000
O	-3.62921800	-2.72224100	1.24189300
O	-5.71029900	1.93158600	0.20504200
O	-5.21109700	-0.97001400	-0.45704800
O	-0.04685900	0.46618000	0.68807500
O	1.60386900	-0.87905000	-0.14015000
O	-3.37785500	0.32057200	-0.80984700
O	4.00024100	-2.78759200	-2.11756300
O	-0.72195400	2.79000300	-1.08622800
H	1.74999300	3.03561300	0.38952000
H	-6.06461600	2.08297700	1.09080100
O	4.51667100	1.64791600	1.23954800
O	5.12495300	-0.34957900	-0.87535200
C	-6.60275400	1.07248500	-0.49827600
C	-6.46056900	-0.39210100	-0.05513100
H	3.73002600	-2.40285700	-2.96024400
H	5.40213100	1.57296200	0.84384400
C	6.34620700	-0.55740000	-0.24826200
C	-8.05103100	1.57298400	-0.39171600
H	-6.28616600	1.13033200	-1.54481100
H	-6.55010700	-0.44750100	1.03637100
C	-7.54770200	-1.27574700	-0.68186400
H	6.21671800	-1.10935200	0.69216100
O	6.91965000	0.72715100	0.03255500
C	7.22928200	-1.36326800	-1.20672500

H	-8.32846100	1.66304700	0.66365100
O	-8.19991000	2.87364100	-0.93430300
C	-9.02085400	0.56298900	-1.00425200
O	-8.82147700	-0.70818400	-0.38992700
C	-7.58283500	-2.71822800	-0.15635300
H	-7.39056700	-1.30833500	-1.77012800
C	8.19427500	0.64419600	0.68470900
C	8.64524000	-1.44932500	-0.64904200
O	6.71583000	-2.66855800	-1.35926000
H	7.24335300	-0.82215000	-2.16456000
H	-7.88859800	2.86481700	-1.84816600
H	-8.84504500	0.46280200	-2.08698100
H	-7.31501200	-2.74261300	0.90138000
H	-8.61584400	-3.06761400	-0.25230800
O	-6.67776000	-3.59070300	-0.81519900
H	8.10651600	0.07198300	1.61750600
C	8.62748000	2.06971000	1.02158400
C	9.16269800	-0.06600800	-0.26194300
H	8.62947200	-2.07725600	0.24663200
O	9.57133300	-2.09073200	-1.52299100
H	5.79540800	-2.60679200	-1.68237300
H	-6.99559900	-3.72511500	-1.71533500
O	9.78087800	2.08982000	1.84841400
H	7.82790900	2.56245100	1.57748600
H	8.78534500	2.62745300	0.08938800
O	10.43076000	-0.17729300	0.38076500
H	9.25599000	0.54628000	-1.17019500
H	9.53418500	-1.65215800	-2.38324300
H	10.43227700	1.50610900	1.43133600
O	-10.31873400	0.97549000	-0.73677600
N	-3.74561900	-2.37824400	2.61690500
O	-3.55483800	-1.23262300	2.94007100
O	-4.05504000	-3.31978700	3.29064100
N	-0.94094100	3.91534200	-0.26329300
O	0.06391900	4.55460200	-0.08714500
O	-2.05379800	4.11666000	0.14989900
H	-10.92234400	0.43211200	-1.25846900
H	10.98351400	-0.72947700	-0.18970300
C	-1.69229600	-1.27958800	0.74904100
O	-1.06206100	-1.85816200	1.59300700

Table S5. Optimized geometry for Model 4.

C	1.16402300	0.95336800	-0.00182000
C	-4.11285200	0.26646400	0.24780300
H	1.00242500	1.32507500	-1.02381100
H	-4.46181200	0.74763700	1.15688900
C	2.19861400	1.80712500	0.73204200
C	-3.19940500	-0.93461100	0.52157500
H	2.23397500	1.45993800	1.76762600
H	-3.05745100	-1.44699700	-0.42851500
C	3.57828100	1.62273300	0.09664100
C	-1.85693700	-0.48982800	1.09428000

H	3.56843700	2.09697700	-0.89730600
H	-2.00316000	0.07575800	2.01294800
C	3.91502900	0.14097700	-0.11330100
C	-1.14498800	0.40808100	0.09030800
H	4.09121800	-0.32441300	0.86212500
H	-0.83756000	-0.18090700	-0.77598200
C	2.77490200	-0.59800800	-0.82007500
H	2.64458100	-0.18275100	-1.82990000
C	2.95530000	-2.11562200	-0.92656900
H	3.30609300	-2.52098200	0.02288600
H	1.97784200	-2.55388100	-1.14467500
O	1.81061200	3.16809500	0.79346800
O	-3.85470800	-1.97700300	1.27244500
O	-6.01531700	2.59005300	0.24152600
O	-1.03676400	-1.56561800	1.60624900
O	-5.15180300	-0.16304600	-0.54762900
O	-0.02740200	0.99110700	0.72736300
O	1.58311900	-0.40461600	-0.05562800
O	-3.39644900	1.28740400	-0.50537300
O	3.91086900	-2.51816000	-1.90542500
H	1.86895400	3.54626500	-0.09312100
H	-6.44499100	2.65255900	1.10435500
O	4.52685800	2.25735900	0.93468000
O	5.08677400	0.01452200	-0.92712200
C	-6.76957200	1.69862400	-0.57247800
C	-6.49297400	0.23280400	-0.21615300
H	3.60897300	-2.23364700	-2.77653900
H	5.40807200	2.10843200	0.55009300
C	6.31250700	-0.15663300	-0.29710600
C	-8.26947600	2.02737600	-0.52223700
H	-6.40397000	1.86500200	-1.59134200
H	-6.66290800	0.08367500	0.85579900
C	-7.42007400	-0.71282000	-0.98935300
H	6.18602500	-0.62247000	0.68926000
O	6.90806900	1.13810600	-0.13572500
C	7.17313100	-1.05550700	-1.19075300
H	-8.60535900	2.02116700	0.52003300
O	-8.53844800	3.33429400	-0.99925400
C	-9.09243600	0.95965500	-1.24456700
O	-8.76579600	-0.32729200	-0.72374700
C	-7.29821900	-2.19255300	-0.59918300
H	-7.20706800	-0.62127300	-2.06461900
C	8.18601400	1.09126700	0.51371900
C	8.59441500	-1.11348600	-0.64268000
O	6.64208400	-2.36215500	-1.22386500
H	7.18334100	-0.59968200	-2.19196600
H	-8.21238300	3.40507800	-1.90552900
H	-8.87403900	0.96791400	-2.32375000
H	-7.08203200	-2.28857700	0.46613000
H	-8.27135900	-2.65563200	-0.79108000
O	-6.25560300	-2.87544800	-1.27890900
H	8.09663600	0.60006900	1.49134400
C	8.64256400	2.53282600	0.72911600

C	9.13553200	0.28945200	-0.37713900
H	8.58073700	-1.66455400	0.30221100
O	9.50107900	-1.83834300	-1.47058900
H	5.71756800	-2.31707800	-1.53751200
H	-6.51220900	-2.96693300	-2.20368800
O	9.80363800	2.60263400	1.54240100
H	7.85514800	3.08070700	1.24964400
H	8.79978600	3.01077600	-0.24648000
O	10.40769400	0.21270900	0.26257100
H	9.22936200	0.82167500	-1.33428700
H	9.46011300	-1.47164700	-2.36385100
H	10.44283000	1.97727200	1.16911400
O	-10.43707700	1.19773800	-0.99718900
N	-4.20628000	-1.71788400	2.63035900
O	-3.97618600	-0.62460400	3.08605000
O	-4.71039900	-2.67086600	3.14753700
N	-0.55294500	-2.54179300	0.69657700
O	-0.92534600	-2.49820100	-0.45123300
O	0.18902500	-3.31743700	1.22583000
H	-10.95733100	0.63747700	-1.58638100
H	10.94754500	-0.39224900	-0.26510400
C	-2.06822400	1.50664900	-0.46133800
O	-1.62007400	2.51976000	-0.92317700

Table S6. Calculated vibrational modes frequencies and activities for the NC Model.

Mode number	Calculated mode frequency, cm^{-1}	Scaled mode frequency, cm^{-1}	IR activity, KM/Mole	Raman activity, A^4/AMU
1	6.52	6.41	0.1377	0.0931
2	9.42	9.26	0.0424	0.1928
3	11.04	10.85	0.1359	0.2546
4	13.51	13.28	0.3389	0.0727
5	14.94	14.69	0.2133	0.3006
6	16.06	15.78	0.3242	0.5478
7	20.99	20.63	1.1639	0.1313
8	23.49	23.09	0.1231	0.4901
9	23.90	23.49	0.8307	0.3745
10	28.45	27.96	0.0894	1.4752
11	29.07	28.57	0.6895	1.4631
12	30.33	29.81	0.9033	0.3125
13	33.96	33.39	1.5818	0.7783
14	34.92	34.33	1.2656	1.2377
15	38.70	38.04	1.4139	0.5045
16	40.56	39.88	4.6268	1.0911
17	42.02	41.31	2.2369	2.0959
18	44.07	43.32	0.2053	1.4630
19	46.59	45.80	0.5556	1.6884
20	51.16	50.29	1.0051	2.4954
21	54.54	53.62	0.6486	1.8622
22	55.77	54.82	0.3089	3.5983
23	59.14	58.13	0.6212	1.7944
24	60.67	59.64	0.5538	1.4224

25	64.47	63.38	2.0748	3.3186
26	64.65	63.56	1.8781	2.8642
27	66.10	64.98	1.7801	3.2370
28	68.75	67.58	1.9279	4.1191
29	71.15	69.94	1.4300	0.9695
30	72.36	71.13	0.3218	5.5910
31	77.40	76.09	1.6099	2.2080
32	79.17	77.82	0.8411	0.7646
33	83.07	81.65	0.7557	1.6464
34	86.28	84.82	0.3221	0.4361
35	87.91	86.42	0.1905	2.8250
36	90.28	88.75	1.8309	4.1912
37	92.02	90.45	1.2362	2.2923
38	94.64	93.03	1.5467	2.5767
39	101.04	99.33	1.7850	1.1737
40	103.65	101.89	1.2802	2.6517
41	104.42	102.65	0.2134	1.7691
42	107.07	105.25	0.6668	3.0066
43	108.48	106.64	0.6418	5.3247
44	111.92	110.02	1.2645	2.0710
45	114.44	112.49	0.2625	2.7031
46	117.95	115.94	0.3525	2.1204
47	123.23	121.14	0.8360	1.7193
48	124.52	122.40	0.6369	2.0108
49	127.65	125.48	0.3344	1.3031
50	142.65	140.22	1.3895	1.7183
51	155.13	152.49	0.4568	0.3304
52	171.39	168.48	0.6096	1.3789
53	172.86	169.92	3.6182	0.8759
54	175.17	172.19	2.0859	2.1270
55	184.15	181.02	1.6219	0.9022
56	184.58	181.44	1.1430	1.7888
57	190.69	187.45	5.4744	0.3913
58	191.82	188.56	1.5721	1.0927
59	192.85	189.58	0.3310	3.6697
60	195.82	192.49	1.3051	0.2669
61	201.68	198.26	3.0295	1.2947
62	207.02	203.50	9.9926	1.1136
63	212.94	209.32	3.2043	1.1614
64	224.94	221.11	6.1099	1.1075
65	238.16	234.11	2.2435	0.8442
66	258.65	254.25	7.6409	1.5837
67	263.66	259.18	11.7970	1.9840
68	268.67	264.10	7.7719	1.2314
69	272.70	268.07	0.2540	1.2179
70	273.60	268.94	12.2758	1.1872
71	274.47	269.80	8.9996	1.9574
72	275.18	270.50	6.8181	1.7419
73	279.60	274.85	15.9100	1.9645
74	282.07	277.28	7.0818	1.3704
75	283.78	278.95	5.7011	6.4931

76	288.51	283.61	0.6349	2.8027
77	294.04	289.05	5.0856	4.0887
78	298.32	293.25	222.5264	3.3341
79	313.51	308.18	15.5226	5.7059
80	319.69	314.26	69.9568	2.3290
81	324.05	318.55	3.8873	2.1920
82	333.61	327.93	23.0695	1.7186
83	337.79	332.05	98.5097	2.7787
84	348.30	342.38	2.3291	8.9730
85	363.83	357.64	7.4328	3.4935
86	366.64	360.41	1.2324	1.5262
87	369.25	362.98	0.7383	4.0259
88	372.01	365.69	3.6933	1.8585
89	373.09	366.75	4.1445	2.2407
90	374.92	368.55	7.9693	1.5431
91	384.90	378.36	4.2783	5.7589
92	388.91	382.30	12.5194	2.6524
93	394.15	387.45	22.7750	0.8241
94	395.10	388.38	18.4410	4.5686
95	406.52	399.61	6.0646	3.7819
96	415.21	408.15	13.6165	3.3170
97	423.72	416.52	24.2666	9.7297
98	427.82	420.54	15.2338	2.9815
99	434.76	427.37	7.1215	1.8560
100	443.42	435.88	3.3378	2.0044
101	464.84	456.93	7.9355	1.4872
102	475.42	467.33	17.9839	3.6763
103	480.39	472.23	4.6878	0.8719
104	498.37	489.90	19.5579	0.7088
105	498.90	490.42	19.0733	5.4957
106	504.66	496.08	23.7704	2.6561
107	507.31	498.69	3.8124	2.3285
108	509.49	500.83	35.2577	2.7498
109	526.09	517.14	18.1881	3.2213
110	536.60	527.48	24.8181	2.6068
111	551.10	541.73	17.7315	4.4161
112	569.01	559.33	14.1074	3.7274
113	583.40	573.48	30.7606	5.7067
114	591.17	581.12	11.0651	1.9873
115	595.99	585.86	0.8336	1.0495
116	601.24	591.02	19.2834	21.8205
117	603.87	593.60	93.8433	21.9885
118	607.30	596.98	48.3021	10.8094
119	608.91	598.56	53.0833	22.6513
120	610.54	600.16	22.0191	39.0260
121	614.64	604.19	38.2090	6.5471
122	619.30	608.77	45.4283	3.0387
123	620.95	610.39	36.8433	9.0319
124	624.43	613.82	60.7987	4.0062
125	626.16	615.51	62.8421	8.4621
126	631.02	620.29	5.8718	12.1236

127	632.45	621.70	12.9878	5.9502
128	641.16	630.26	208.8389	3.0425
129	650.73	639.66	152.5383	4.7418
130	667.00	655.66	4.0858	1.5565
131	672.89	661.45	30.3771	4.9964
132	690.63	678.89	41.8630	4.1154
133	693.73	681.93	45.4479	7.0768
134	711.19	699.10	4.4379	5.1195
135	723.55	711.25	0.0344	5.3910
136	737.74	725.19	28.4186	11.2472
137	748.12	735.41	7.7735	7.5913
138	754.70	741.87	5.4501	4.8919
139	759.22	746.32	60.5919	11.4075
140	763.41	750.43	37.7148	1.1368
141	766.82	753.78	62.9673	6.1712
142	768.31	755.25	100.9453	4.1263
143	772.40	759.27	53.3939	1.2451
144	773.13	759.98	44.0819	0.9055
145	773.54	760.39	45.8057	0.2412
146	773.69	760.53	16.2758	0.1920
147	774.32	761.16	11.2149	0.5564
148	774.64	761.47	44.8325	2.3283
149	775.53	762.35	20.5868	2.8209
150	775.77	762.58	39.8967	0.7212
151	777.21	764.00	28.7916	0.5968
152	778.03	764.80	25.7684	1.7406
153	781.78	768.49	77.4266	6.9733
154	784.18	770.85	74.7833	3.9301
155	786.71	773.33	73.9831	4.8136
156	839.83	825.55	483.1327	4.9970
157	841.34	827.04	642.4485	4.7773
158	841.73	827.42	418.3213	5.8200
159	842.23	827.91	193.2871	14.8264
160	845.17	830.80	454.0199	14.4803
161	847.14	832.74	328.0544	9.7932
162	847.83	833.42	623.4035	14.5988
163	852.56	838.07	300.6830	5.0100
164	854.77	840.24	203.4403	21.1451
165	855.43	840.89	102.8871	26.2636
166	858.47	843.87	134.5661	20.1801
167	861.71	847.06	98.3714	107.3632
168	910.60	895.12	3.4571	8.3763
169	915.07	899.51	3.2646	6.0719
170	917.84	902.24	40.9047	11.5672
171	920.15	904.50	26.4582	10.1898
172	938.27	922.32	32.2285	5.3940
173	941.59	925.58	27.7043	9.7194
174	945.92	929.84	124.2465	1.1748
175	952.43	936.24	193.1258	4.9007
176	961.80	945.45	158.4880	2.3396
177	972.72	956.18	195.4173	5.0025

178	974.91	958.33	82.0365	3.7036
179	976.95	960.34	110.3598	3.9286
180	993.28	976.40	133.2127	2.2678
181	995.93	979.00	176.7781	0.5241
182	997.56	980.60	52.1880	2.7869
183	1001.78	984.75	58.6085	3.4920
184	1015.52	998.26	34.7484	4.6924
185	1029.54	1012.04	90.5423	1.9427
186	1032.14	1014.60	430.0707	26.0604
187	1035.96	1018.35	348.3448	11.8440
188	1040.34	1022.65	422.5809	12.9829
189	1044.01	1026.26	356.3107	16.6528
190	1048.76	1030.93	564.9786	7.4212
191	1053.99	1036.07	192.2760	6.7294
192	1060.17	1042.15	187.8082	13.2085
193	1062.70	1044.64	76.9087	7.5267
194	1070.81	1052.61	41.4875	18.6712
195	1074.50	1056.23	107.8606	9.6947
196	1077.37	1059.05	118.2884	8.7260
197	1084.47	1066.03	92.0649	9.9250
198	1085.87	1067.41	219.5458	7.4578
199	1087.46	1068.98	85.0827	4.5953
200	1100.01	1081.31	5.8519	3.8993
201	1103.13	1084.38	66.5614	6.9052
202	1106.57	1087.76	38.2280	5.2422
203	1109.08	1090.22	23.7850	6.4654
204	1114.53	1095.58	40.3909	5.8064
205	1120.95	1101.90	96.5865	15.3487
206	1123.35	1104.26	55.6115	14.6825
207	1125.34	1106.21	185.7850	7.4671
208	1131.03	1111.80	8.1434	19.5089
209	1132.28	1113.04	2.7709	12.2096
210	1136.34	1117.02	33.8257	9.7142
211	1137.91	1118.56	5.3272	12.8707
212	1138.10	1118.75	19.8971	8.9211
213	1147.83	1128.32	7.4327	3.1051
214	1154.82	1135.19	146.0354	12.3180
215	1158.15	1138.46	25.5278	3.4550
216	1170.83	1150.93	105.9344	11.1745
217	1174.32	1154.35	182.7966	24.1566
218	1175.43	1155.45	40.1253	4.1428
219	1177.06	1157.05	101.0265	7.0121
220	1229.91	1209.00	4.1004	4.3456
221	1236.22	1215.20	17.3876	1.5916
222	1240.88	1219.78	22.6087	3.0149
223	1241.56	1220.46	4.1828	0.6230
224	1268.69	1247.12	14.5857	5.5879
225	1268.95	1247.37	7.1037	3.7968
226	1276.82	1255.12	12.5621	3.9340
227	1281.19	1259.41	8.6621	2.1194
228	1289.35	1267.43	62.8379	3.6400

229	1294.60	1272.60	166.0590	5.5282
230	1306.81	1284.59	71.8152	6.6805
231	1308.72	1286.47	136.1310	5.6849
232	1309.09	1286.84	262.4439	10.2579
233	1310.30	1288.02	347.2301	5.7377
234	1311.23	1288.94	210.7112	8.8082
235	1311.73	1289.43	317.4448	13.9407
236	1312.03	1289.73	388.2294	20.6824
237	1312.85	1290.53	197.2454	9.6021
238	1314.81	1292.46	218.5767	44.5571
239	1316.14	1293.77	402.9444	13.0688
240	1317.85	1295.45	52.5037	18.6889
241	1320.87	1298.41	17.7917	4.0768
242	1321.31	1298.85	138.7324	36.6739
243	1327.85	1305.27	19.8234	21.0324
244	1329.50	1306.90	56.2915	6.9842
245	1330.59	1307.97	45.5125	11.3794
246	1332.69	1310.03	11.8021	2.7740
247	1335.94	1313.23	128.8779	22.2563
248	1340.13	1317.35	67.0159	13.6945
249	1340.75	1317.96	3.1569	7.9899
250	1342.20	1319.39	50.8610	7.9578
251	1346.41	1323.52	91.3208	9.8736
252	1348.05	1325.14	21.0058	2.4013
253	1348.62	1325.69	34.7817	24.8593
254	1361.24	1338.10	71.2836	14.7511
255	1367.24	1344.00	100.5576	6.6371
256	1369.97	1346.68	82.1202	9.2027
257	1372.78	1349.44	60.3144	21.9189
258	1374.55	1351.18	98.3495	7.3949
259	1377.45	1354.04	63.7886	29.1918
260	1379.24	1355.79	95.4185	11.3442
261	1394.36	1370.66	10.0845	8.6176
262	1398.33	1374.56	9.7077	10.4676
263	1399.30	1375.51	6.3847	26.0886
264	1400.89	1377.07	25.5645	19.1217
265	1401.92	1378.08	11.1600	32.8280
266	1403.95	1380.08	13.1672	24.6052
267	1406.18	1382.27	7.7740	12.1887
268	1406.28	1382.38	8.5769	17.6312
269	1410.39	1386.41	0.1480	17.7790
270	1413.63	1389.60	30.4526	1.8573
271	1417.57	1393.47	23.1785	5.9435
272	1419.27	1395.14	12.3254	10.5895
273	1425.43	1401.20	10.4061	8.5725
274	1425.53	1401.29	9.8967	10.3175
275	1427.59	1403.33	8.4724	2.7774
276	1431.93	1407.58	0.6491	13.3233
277	1439.99	1415.51	38.6030	10.7104
278	1442.20	1417.69	1.8347	13.9180
279	1446.70	1422.11	17.0963	1.7909

280	1447.93	1423.31	15.7822	4.5323
281	1471.62	1446.61	27.5951	15.1898
282	1474.98	1449.90	38.6517	13.2273
283	1476.22	1451.12	21.1837	4.6282
284	1476.76	1451.65	43.9373	14.3209
285	1478.79	1453.65	33.1055	12.9106
286	1677.53	1649.02	659.2013	8.0883
287	1678.85	1650.31	629.1071	6.3930
288	1680.59	1652.02	431.4501	4.6358
289	1681.86	1653.26	711.3787	6.5175
290	1682.16	1653.57	778.6176	9.1453
291	1682.55	1653.95	1154.1272	4.3762
292	1685.15	1656.51	347.0892	2.2267
293	1685.88	1657.22	681.6176	7.0017
294	1687.79	1659.10	357.9479	2.4687
295	1692.42	1663.65	663.7718	5.1501
296	1696.16	1667.32	1169.7336	8.0206
297	1697.14	1668.29	597.7017	6.4502
298	3020.43	2969.09	22.1456	197.0769
299	3025.40	2973.97	23.2125	160.9984
300	3026.66	2975.21	21.3817	190.2015
301	3029.08	2977.59	30.2208	184.5569
302	3047.77	2995.96	28.0855	101.5860
303	3051.48	2999.61	23.7716	103.9276
304	3059.37	3007.36	31.4475	112.4241
305	3060.44	3008.41	24.7503	103.5305
306	3065.02	3012.91	21.8704	129.3305
307	3083.16	3030.75	10.9508	69.9422
308	3089.51	3036.99	16.2901	257.6218
309	3090.48	3037.94	10.8092	208.9543
310	3092.30	3039.73	7.9434	218.9788
311	3097.08	3044.43	3.2567	84.7402
312	3099.20	3046.52	1.3314	242.5882
313	3102.14	3049.41	9.5452	373.1957
314	3103.28	3050.53	0.5915	142.9933
315	3112.77	3059.85	3.2800	14.5936
316	3118.28	3065.27	5.0228	10.0683
317	3120.79	3067.73	0.3641	195.7856
318	3127.34	3074.17	3.9514	46.5858
319	3129.09	3075.90	3.5441	62.0853
320	3132.04	3078.80	4.1549	81.7230
321	3133.39	3080.12	4.8821	18.3024
322	3146.74	3093.25	3.1898	115.2515
323	3149.83	3096.28	0.9645	102.5304
324	3152.64	3099.04	0.9445	85.9882
325	3153.45	3099.84	1.2978	98.5582
326	3807.92	3743.18	118.9565	209.5341
327	3808.40	3743.66	127.7452	161.1776

Table S7. Calculated vibrational modes frequencies and activities for the Model 1.

Mode number	Calculated mode frequency, cm^{-1}	Scaled mode frequency, cm^{-1}	IR activity, KM/Mole	Raman activity, A^4/AMU
1	8.48	8.33	0.0621	0.1344
2	12.38	12.17	0.0631	0.1628
3	15.68	15.41	0.2327	0.1758
4	21.59	21.22	0.3742	0.1085
5	23.22	22.82	0.3555	0.3253
6	29.14	28.64	1.1991	0.2003
7	30.23	29.72	1.0544	0.1034
8	36.67	36.05	1.5685	0.3411
9	39.24	38.57	0.6919	0.1651
10	47.14	46.34	1.9362	0.8129
11	51.24	50.37	1.1650	1.8376
12	54.76	53.83	2.6065	1.3826
13	59.79	58.77	0.4222	1.4150
14	62.14	61.08	1.4710	1.1713
15	71.12	69.91	4.1868	0.5434
16	75.17	73.89	4.1186	3.2792
17	76.87	75.57	0.4160	1.9922
18	83.95	82.53	4.7954	0.6537
19	85.24	83.79	4.8628	0.8651
20	91.02	89.47	1.9069	2.0020
21	93.68	92.09	2.7948	0.6467
22	95.30	93.68	3.5221	0.4886
23	101.67	99.95	2.7068	0.7947
24	108.76	106.91	0.1222	2.1828
25	117.37	115.38	1.0275	2.0560
26	122.06	119.98	1.6981	1.1798
27	127.86	125.69	4.0134	0.2074
28	134.29	132.01	0.0936	1.7590
29	151.30	148.73	1.1564	0.8066
30	153.76	151.14	3.4073	0.3634
31	156.64	153.97	6.9027	0.5997
32	159.85	157.13	8.5764	0.3757
33	172.97	170.03	1.8334	0.4901
34	186.60	183.43	0.6486	1.1320
35	190.40	187.16	0.3085	1.2818
36	194.36	191.06	9.3051	1.4297
37	204.39	200.92	5.1709	0.5258
38	210.56	206.98	1.8363	1.0188
39	217.09	213.40	20.2749	0.6824
40	221.17	217.41	12.6111	0.4253
41	231.76	227.82	63.6614	1.2622
42	238.03	233.99	48.1088	1.2004
43	249.09	244.86	5.8566	2.0117
44	260.22	255.80	11.0282	1.0115
45	271.09	266.48	11.7440	2.1368
46	276.09	271.39	22.7775	2.7727
47	278.76	274.02	95.5970	2.5132
48	282.20	277.40	52.5709	3.6130
49	285.22	280.37	3.6091	0.6667

50	289.43	284.51	18.2085	1.6603
51	289.76	284.84	54.9141	3.3611
52	296.25	291.21	30.3782	1.0668
53	301.22	296.10	59.5961	0.9396
54	309.85	304.58	57.5700	2.4891
55	314.90	309.55	104.4226	0.9406
56	317.28	311.88	35.2516	0.9318
57	324.99	319.47	185.4431	1.8828
58	329.46	323.86	34.4810	3.3317
59	329.93	324.32	29.5361	7.2038
60	336.94	331.21	49.0350	5.6671
61	337.28	331.55	96.9281	1.4907
62	346.66	340.76	22.6284	9.7080
63	348.18	342.26	76.5218	3.7019
64	352.95	346.95	48.0173	5.8421
65	361.38	355.24	135.7673	3.3098
66	375.82	369.44	43.7142	2.3989
67	385.98	379.42	75.0497	3.1149
68	393.60	386.91	12.9527	2.0959
69	395.25	388.53	9.0235	2.0115
70	401.68	394.85	35.4037	2.8918
71	404.31	397.43	214.5470	4.9693
72	424.56	417.34	5.5859	2.3295
73	429.20	421.90	56.6751	7.9947
74	431.65	424.31	9.7439	4.6760
75	432.21	424.86	0.7556	1.6766
76	436.66	429.23	17.2382	6.1436
77	449.17	441.54	5.2603	1.6897
78	452.99	445.29	1.2625	10.7559
79	471.98	463.96	2.5940	4.1964
80	481.05	472.88	29.0487	3.2541
81	489.04	480.73	150.6486	5.5594
82	497.28	488.83	27.9494	1.5840
83	500.44	491.93	3.9955	2.5488
84	517.71	508.90	5.7979	2.3228
85	527.41	518.45	11.0534	4.0832
86	551.35	541.98	33.5297	2.2675
87	555.05	545.61	58.8801	2.7460
88	559.34	549.83	47.4992	1.4144
89	572.92	563.18	135.0319	2.8092
90	580.16	570.30	26.1975	3.2462
91	584.16	574.23	52.2472	1.5443
92	594.26	584.15	20.2969	2.8049
93	605.84	595.54	35.7843	2.9585
94	607.60	597.27	37.3007	5.4950
95	614.94	604.49	0.9259	19.8086
96	620.49	609.94	26.7915	4.1766
97	627.71	617.04	41.8943	1.9176
98	636.30	625.48	35.6050	7.8742
99	641.73	630.82	9.9348	0.8100
100	654.61	643.48	134.6351	4.1966

101	663.41	652.13	6.7084	0.2482
102	684.09	672.46	20.1176	3.5591
103	702.57	690.63	10.6412	1.6134
104	710.70	698.62	138.8711	0.7345
105	725.27	712.94	0.7815	2.1697
106	754.15	741.33	10.3063	8.6047
107	774.24	761.08	21.9685	1.3592
108	774.49	761.33	65.8464	0.8208
109	777.45	764.23	28.1541	1.7384
110	778.31	765.08	77.2317	5.3883
111	848.23	833.81	460.7497	6.8175
112	854.64	840.11	440.7463	13.5801
113	861.75	847.10	91.1371	39.6162
114	898.37	883.10	3.2155	2.2089
115	914.15	898.61	1.9722	7.2129
116	918.58	902.97	36.4760	4.5278
117	919.12	903.49	8.2191	2.1894
118	942.46	926.44	74.3954	5.9009
119	963.41	947.03	80.1843	4.3489
120	969.95	953.46	93.5179	1.7793
121	982.02	965.32	150.6117	2.9612
122	990.21	973.37	43.4125	0.8132
123	994.14	977.24	233.9196	7.4888
124	998.69	981.71	144.9861	1.9403
125	999.17	982.18	25.2851	3.1442
126	1003.40	986.34	111.0462	0.7726
127	1013.33	996.10	21.9967	2.8288
128	1022.14	1004.76	226.4409	3.4045
129	1028.46	1010.98	101.7902	0.8550
130	1031.18	1013.65	5.7941	2.8781
131	1036.02	1018.41	514.6949	18.3000
132	1039.45	1021.78	198.8723	5.3982
133	1045.37	1027.59	260.4935	4.7933
134	1049.17	1031.33	295.3162	4.1704
135	1053.22	1035.31	768.4393	1.7176
136	1056.01	1038.06	351.4290	3.7567
137	1059.38	1041.37	110.5806	4.0986
138	1063.41	1045.33	45.6598	6.2778
139	1067.21	1049.07	344.8608	8.4970
140	1067.86	1049.71	104.8172	3.5870
141	1070.05	1051.86	202.6097	2.1222
142	1073.81	1055.55	36.1469	16.3712
143	1078.88	1060.53	67.1396	23.3786
144	1089.87	1071.34	113.8306	9.4471
145	1094.61	1076.00	12.9929	12.5550
146	1103.72	1084.96	100.5160	7.8484
147	1104.77	1085.99	108.7377	19.1010
148	1107.10	1088.28	249.7755	8.1262
149	1108.14	1089.30	94.6610	9.4991
150	1111.98	1093.08	2.2984	10.9648
151	1114.13	1095.19	74.5861	11.5900

152	1119.00	1099.97	25.5637	19.2599
153	1119.78	1100.75	9.8039	7.2067
154	1123.86	1104.76	82.1374	5.6400
155	1125.77	1106.63	62.7578	2.1115
156	1131.56	1112.33	9.2867	10.2082
157	1132.67	1113.42	47.7563	20.5405
158	1136.09	1116.77	15.0975	5.9523
159	1142.28	1122.86	41.6807	8.6678
160	1145.70	1126.22	52.4667	2.3401
161	1152.66	1133.07	64.6685	6.9907
162	1154.90	1135.27	93.4379	15.7043
163	1166.66	1146.83	103.8246	10.3808
164	1177.30	1157.28	11.2975	6.1852
165	1180.59	1160.52	128.5774	8.3521
166	1203.30	1182.84	20.2953	2.7381
167	1203.69	1183.22	1.1995	10.7055
168	1207.52	1186.99	68.8061	3.1949
169	1219.13	1198.40	54.5689	1.1419
170	1227.27	1206.40	47.5776	3.1262
171	1233.16	1212.20	41.8390	6.1890
172	1236.54	1215.52	20.8217	3.9115
173	1238.28	1217.23	7.7529	9.7957
174	1266.19	1244.67	13.0231	2.7816
175	1271.61	1250.00	35.3245	3.5793
176	1273.36	1251.71	61.5458	7.1805
177	1274.59	1252.92	11.9352	3.8759
178	1278.48	1256.75	13.7169	1.4883
179	1294.21	1272.21	18.9100	3.7605
180	1299.48	1277.39	30.8622	1.1555
181	1305.42	1283.23	101.9655	10.7792
182	1308.36	1286.12	138.7365	8.4257
183	1311.28	1288.99	476.4424	12.0300
184	1313.92	1291.58	21.2331	20.0515
185	1314.20	1291.85	42.5958	7.5114
186	1319.14	1296.71	3.5267	10.8517
187	1322.67	1300.19	23.7391	10.0639
188	1326.35	1303.80	17.5487	5.2067
189	1331.85	1309.21	98.2327	19.5860
190	1334.08	1311.40	23.1160	9.2391
191	1336.34	1313.62	33.7144	5.2543
192	1336.73	1314.00	18.5603	9.1043
193	1339.59	1316.82	24.2984	5.6730
194	1348.74	1325.81	30.9455	13.1978
195	1353.13	1330.13	6.5156	10.8706
196	1356.32	1333.26	45.8464	16.7488
197	1359.14	1336.04	70.2989	9.8711
198	1367.40	1344.15	25.2555	8.8640
199	1369.58	1346.30	58.5237	29.0706
200	1381.63	1358.14	32.1672	4.6843
201	1383.39	1359.87	29.6154	3.3675
202	1387.98	1364.38	0.5478	5.7189

203	1390.95	1367.31	49.3417	12.3489
204	1393.99	1370.29	21.8863	15.5070
205	1395.67	1371.95	7.7827	24.4436
206	1398.97	1375.19	32.8496	18.9672
207	1400.16	1376.36	22.1779	7.6073
208	1403.10	1379.24	1.8760	21.6358
209	1404.73	1380.85	24.1493	5.9631
210	1406.24	1382.33	7.3127	17.2610
211	1410.14	1386.16	5.5454	3.0297
212	1415.79	1391.72	13.5207	8.3427
213	1418.93	1394.81	67.5626	12.2126
214	1420.03	1395.89	53.1408	8.9993
215	1420.88	1396.72	56.1750	11.0101
216	1421.94	1397.77	40.4772	6.7407
217	1423.28	1399.09	57.7804	5.2543
218	1424.88	1400.65	38.4175	4.3771
219	1428.46	1404.18	27.0294	6.5961
220	1434.12	1409.74	13.3603	9.0635
221	1442.67	1418.15	8.9523	6.7552
222	1443.74	1419.19	10.9046	19.8400
223	1451.85	1427.16	41.5714	4.4814
224	1460.62	1435.79	32.6438	3.9714
225	1464.28	1439.39	101.5198	10.5937
226	1469.76	1444.77	52.0829	3.5632
227	1473.69	1448.64	34.5885	14.8457
228	1475.50	1450.42	5.2146	6.4389
229	1503.71	1478.14	2.7429	13.6931
230	1505.35	1479.76	2.7573	9.5871
231	1512.10	1486.39	0.9278	13.6212
232	1675.63	1647.15	544.8381	7.3187
233	1678.06	1649.54	1049.5379	4.3711
234	1678.77	1650.23	442.9034	5.3987
235	2949.95	2899.80	83.2128	200.6735
236	2959.84	2909.52	37.3289	142.7594
237	2965.82	2915.40	9.9044	53.8700
238	2972.62	2922.08	49.2971	242.3949
239	2974.67	2924.10	36.8696	420.7059
240	2975.72	2925.13	43.9176	236.1772
241	2980.24	2929.58	111.3724	352.7014
242	2985.64	2934.88	43.0175	353.4741
243	2993.30	2942.42	66.0006	76.1118
244	3001.65	2950.62	119.1958	23.3605
245	3013.61	2962.38	23.1280	136.5761
246	3018.99	2967.67	23.1803	161.4456
247	3019.89	2968.55	72.6138	331.2246
248	3028.08	2976.60	44.3231	64.2326
249	3031.03	2979.50	2.6184	261.1880
250	3042.20	2990.49	53.9505	287.8899
251	3045.17	2993.41	97.9439	20.8270
252	3050.56	2998.70	55.4167	167.4215
253	3062.93	3010.86	46.8022	167.5756

254	3076.98	3024.67	54.8504	201.9642
255	3084.33	3031.90	46.0557	123.8280
256	3088.31	3035.80	20.5042	69.9850
257	3093.41	3040.82	10.1626	205.5372
258	3099.07	3046.38	3.2335	313.2825
259	3101.54	3048.81	32.1984	118.4888
260	3110.41	3057.54	3.9790	20.7112
261	3129.45	3076.25	2.9016	50.5596
262	3152.21	3098.62	1.8689	107.2075
263	3555.12	3494.69	844.0131	318.8377
264	3638.53	3576.68	680.0258	305.7375
265	3718.85	3655.63	232.1847	116.6456
266	3769.11	3705.04	76.4725	168.7220
267	3780.54	3716.27	98.5799	141.3791
268	3783.12	3718.81	42.2440	111.3359
269	3789.72	3725.30	71.0236	83.7535
270	3792.15	3727.68	32.3052	151.7212
271	3802.64	3738.00	79.9559	224.0313
272	3806.84	3742.13	73.1566	119.3681
273	3811.31	3746.52	57.2469	127.8175

Table S8. Calculated vibrational modes frequencies and activities for the Model 2.

Mode number	Calculated mode frequency, cm^{-1}	Scaled mode frequency, cm^{-1}	IR activity, KM/Mole	Raman activity, A^4/AMU
1	9.23	9.08	0.0144	0.0870
2	10.47	10.29	0.1066	0.0400
3	16.55	16.27	0.3777	0.2251
4	21.58	21.21	0.6740	0.1209
5	26.27	25.83	1.2585	0.1063
6	30.27	29.76	0.2472	0.2570
7	33.90	33.33	1.9620	0.1243
8	38.42	37.77	1.9203	0.0887
9	42.87	42.14	0.5309	0.8083
10	52.41	51.52	1.3491	0.3727
11	54.27	53.35	6.7274	0.6885
12	57.43	56.45	0.0429	0.1903
13	66.59	65.46	1.6860	0.7987
14	70.07	68.88	2.5178	2.0168
15	71.33	70.11	1.8940	2.8427
16	76.56	75.26	8.3025	0.9005
17	82.13	80.74	1.5892	0.8803
18	90.83	89.28	6.3778	1.2992
19	93.31	91.72	3.2262	0.6692
20	101.72	99.99	2.1582	0.4131
21	106.84	105.02	2.3770	0.5970
22	112.92	111.00	0.4588	0.2606
23	119.24	117.22	2.8991	1.6396
24	123.33	121.23	4.8457	0.7336
25	133.64	131.36	0.7484	1.9415
26	145.17	142.70	2.9149	1.9462

27	149.83	147.28	0.9804	0.6165
28	153.36	150.75	1.5738	0.3314
29	154.93	152.30	3.9592	0.5523
30	157.56	154.88	11.3806	0.7137
31	171.95	169.03	2.2010	0.2894
32	190.45	187.21	0.9343	1.1777
33	193.64	190.35	4.7769	1.0899
34	203.36	199.90	3.0397	0.3825
35	210.32	206.75	3.6993	1.4083
36	216.77	213.08	6.3607	0.5357
37	223.22	219.42	10.4570	0.3497
38	242.80	238.67	1.5846	0.4206
39	249.23	245.00	14.5304	1.5327
40	252.98	248.68	73.7958	2.7190
41	262.07	257.62	11.4004	0.5479
42	267.86	263.31	10.7361	1.2003
43	279.73	274.97	69.7797	0.7119
44	283.22	278.41	39.8358	4.6418
45	285.53	280.68	74.5142	1.2709
46	292.12	287.16	13.8248	2.7189
47	295.36	290.34	16.1882	1.1270
48	297.92	292.86	69.2126	2.2465
49	301.86	296.73	122.2543	2.7866
50	309.57	304.30	88.0716	2.6640
51	314.54	309.20	118.7992	1.3654
52	317.74	312.34	22.8636	1.8918
53	319.34	313.91	32.1363	0.9745
54	322.43	316.95	194.1395	1.3835
55	325.38	319.84	72.6195	2.7775
56	335.18	329.48	5.8021	9.8496
57	336.43	330.71	10.7873	8.2948
58	338.40	332.65	16.4717	7.2417
59	349.52	343.58	45.5210	6.4162
60	351.41	345.44	56.6637	3.8119
61	362.47	356.31	152.9331	3.5164
62	364.17	357.98	68.8220	0.9123
63	369.88	363.59	48.9260	1.1746
64	385.45	378.89	41.3788	1.7024
65	391.07	384.42	42.6100	5.0620
66	399.99	393.19	15.4736	1.2424
67	414.35	407.31	115.1935	5.0320
68	414.68	407.63	44.2813	1.7058
69	428.87	421.58	23.1957	10.0196
70	432.35	425.00	25.4153	2.3642
71	433.88	426.50	53.4017	2.8696
72	439.41	431.94	29.8449	1.1041
73	444.91	437.35	8.0551	4.2192
74	450.75	443.09	0.8079	8.6305
75	466.99	459.06	8.1075	9.9495
76	474.19	466.13	7.5980	2.2744
77	480.40	472.23	78.1723	2.6214

78	484.98	476.74	121.6265	6.6625
79	494.76	486.35	22.1862	0.4658
80	508.29	499.65	4.4517	6.4744
81	518.62	509.80	18.0094	1.7977
82	551.82	542.44	19.2206	0.5270
83	557.69	548.21	83.4955	2.5641
84	559.10	549.59	95.8570	1.5513
85	572.97	563.23	76.0331	2.4014
86	580.06	570.19	20.2389	2.7782
87	586.22	576.26	40.5353	0.6735
88	595.77	585.64	0.4513	2.4340
89	606.26	595.95	31.1415	1.9179
90	608.36	598.02	9.1189	5.0823
91	618.14	607.63	18.5916	15.3493
92	626.83	616.18	54.8754	5.3214
93	638.88	628.02	22.5887	2.0182
94	647.41	636.41	116.9621	4.9955
95	648.99	637.96	20.5197	1.1548
96	662.12	650.86	18.8098	0.9854
97	692.27	680.51	26.4121	5.5619
98	702.91	690.96	7.7845	1.9064
99	707.83	695.80	55.0816	8.2632
100	708.64	696.59	85.7676	4.5767
101	732.41	719.96	7.4970	2.1848
102	767.41	754.36	34.8773	4.9740
103	775.08	761.90	20.6415	0.1214
104	794.92	781.41	17.4519	4.5228
105	853.28	838.78	547.8278	5.8509
106	856.17	841.62	106.0291	33.8892
107	899.63	884.33	3.2392	2.1275
108	911.55	896.05	13.1596	8.9299
109	916.01	900.44	38.9474	4.5760
110	918.70	903.08	3.3938	1.4800
111	934.94	919.04	50.6079	2.2084
112	968.59	952.12	13.1923	3.5179
113	979.45	962.80	97.6263	0.5629
114	982.81	966.10	72.8363	5.2196
115	989.51	972.68	89.5601	3.8954
116	995.16	978.24	223.0297	6.4200
117	998.96	981.98	151.2621	0.8484
118	1000.00	983.00	85.4513	5.2605
119	1004.03	986.96	86.2552	0.7851
120	1016.30	999.02	34.8145	3.9905
121	1023.85	1006.44	42.5334	1.8162
122	1027.06	1009.60	44.2167	10.0123
123	1029.97	1012.46	498.6393	12.7146
124	1032.21	1014.66	7.8657	4.1196
125	1039.30	1021.63	395.9590	3.0614
126	1046.62	1028.82	199.4062	2.7672
127	1049.53	1031.69	226.0511	11.3858
128	1051.97	1034.09	884.7809	2.9753

129	1056.29	1038.33	246.6543	5.1220
130	1059.44	1041.43	522.4626	3.6852
131	1060.29	1042.26	57.2715	3.1727
132	1063.90	1045.82	196.2306	4.4566
133	1067.84	1049.69	80.2968	5.5199
134	1073.34	1055.09	192.4747	2.8130
135	1074.43	1056.16	20.5489	17.0182
136	1078.63	1060.30	98.2042	21.8528
137	1088.05	1069.55	100.6496	5.4794
138	1094.25	1075.65	12.0059	12.0642
139	1104.57	1085.79	94.6378	10.4325
140	1105.36	1086.57	60.3216	18.7859
141	1108.54	1089.69	226.0207	6.4175
142	1109.31	1090.45	72.5667	13.6533
143	1114.33	1095.39	54.2044	13.0505
144	1116.27	1097.29	33.8136	16.1147
145	1117.05	1098.06	67.6416	2.8569
146	1120.38	1101.33	28.6087	9.1386
147	1127.02	1107.86	67.3846	2.6946
148	1130.64	1111.42	23.9817	8.6892
149	1134.16	1114.88	39.9385	10.4000
150	1136.10	1116.78	12.5645	3.9714
151	1139.04	1119.68	15.9894	19.5115
152	1144.57	1125.11	34.9166	2.6400
153	1151.02	1131.45	65.9698	6.4645
154	1152.64	1133.05	8.6005	7.8632
155	1160.27	1140.55	137.3294	11.0407
156	1176.42	1156.42	57.3207	6.9609
157	1201.17	1180.75	143.2955	4.1762
158	1204.91	1184.42	5.3386	9.2178
159	1205.85	1185.36	56.8875	3.7895
160	1208.58	1188.04	17.7737	4.9759
161	1218.88	1198.16	75.6493	1.8688
162	1228.49	1207.61	41.7671	4.1329
163	1230.09	1209.17	49.4916	9.2342
164	1237.89	1216.85	6.0176	8.3907
165	1249.87	1228.62	19.5272	1.5009
166	1266.40	1244.87	13.0108	3.1779
167	1270.29	1248.70	9.5289	3.2710
168	1272.55	1250.92	26.0483	3.4404
169	1275.24	1253.56	69.2548	6.9162
170	1283.59	1261.77	5.7456	8.0040
171	1295.38	1273.36	24.7857	5.9639
172	1301.99	1279.85	29.5521	1.0887
173	1305.69	1283.49	141.4202	14.6959
174	1306.12	1283.92	51.1589	5.4617
175	1314.68	1292.33	195.2430	9.7618
176	1315.82	1293.45	17.0853	3.5061
177	1316.37	1293.99	147.4381	25.1078
178	1318.27	1295.86	27.0863	9.8195
179	1323.30	1300.80	6.8145	14.4415

180	1328.61	1306.03	105.9337	7.5441
181	1335.01	1312.32	40.8336	5.9158
182	1338.67	1315.91	20.5075	10.6691
183	1341.34	1318.54	19.4008	6.5340
184	1350.30	1327.34	35.6790	14.9456
185	1351.58	1328.60	17.6571	19.4226
186	1354.33	1331.31	14.4057	9.1404
187	1357.92	1334.83	8.4346	6.7734
188	1363.43	1340.25	76.3449	22.5559
189	1370.82	1347.52	11.7067	8.3860
190	1378.08	1354.65	34.5344	4.7926
191	1384.65	1361.11	28.4481	4.4741
192	1387.85	1364.26	8.5663	12.7781
193	1390.45	1366.82	42.3830	20.1062
194	1393.61	1369.92	10.8513	3.7523
195	1394.29	1370.59	27.5458	15.6434
196	1395.82	1372.09	3.6905	15.5049
197	1399.04	1375.26	8.2908	4.8739
198	1403.74	1379.88	34.1279	14.3394
199	1405.98	1382.08	20.8661	9.7352
200	1408.54	1384.60	5.7356	3.3081
201	1410.21	1386.23	6.6836	17.8406
202	1412.79	1388.77	2.8143	18.0818
203	1419.14	1395.01	56.8263	5.8300
204	1422.04	1397.87	74.7610	6.2327
205	1422.17	1397.99	55.3206	9.0521
206	1423.02	1398.83	56.9286	7.9261
207	1424.95	1400.73	27.3693	5.6478
208	1429.47	1405.17	50.2125	6.9180
209	1436.60	1412.18	11.5872	8.1547
210	1440.22	1415.73	13.9131	17.2708
211	1445.85	1421.27	27.1266	9.1386
212	1446.09	1421.50	3.3377	5.8715
213	1461.04	1436.20	37.8553	4.8274
214	1464.84	1439.94	91.3988	9.6779
215	1468.00	1443.05	54.7119	3.2856
216	1472.60	1447.57	4.9859	6.8977
217	1474.85	1449.77	39.2323	14.5866
218	1498.62	1473.14	3.7569	13.5005
219	1505.03	1479.45	2.5684	10.0182
220	1513.44	1487.71	0.8742	13.4489
221	1678.02	1649.49	604.8224	3.7982
222	1682.21	1653.62	742.0383	6.6994
223	1820.68	1789.73	269.9252	44.1179
224	2949.59	2899.45	85.5397	202.3113
225	2962.90	2912.54	36.5584	128.5644
226	2968.24	2917.78	22.5213	153.1687
227	2975.71	2925.12	54.1773	223.6029
228	2977.26	2926.65	43.9685	447.7921
229	2977.82	2927.20	45.4103	225.2434
230	2980.07	2929.41	80.0171	237.8841

231	2985.72	2934.96	46.7136	337.3828
232	2992.88	2942.00	57.0061	85.4931
233	3002.40	2951.36	127.0368	23.8037
234	3003.46	2952.40	27.0745	131.0110
235	3010.14	2958.96	42.6823	180.9041
236	3020.96	2969.60	21.5861	162.6562
237	3024.85	2973.43	62.6529	272.8144
238	3028.97	2977.47	44.8927	67.2370
239	3030.94	2979.42	0.4338	306.7546
240	3040.02	2988.34	57.5168	295.5489
241	3043.60	2991.85	5.2042	210.5413
242	3045.38	2993.61	95.3755	9.9240
243	3049.52	2997.68	55.2668	161.0275
244	3063.62	3011.54	45.8404	162.5817
245	3077.12	3024.81	54.2053	203.1296
246	3091.86	3039.30	42.1091	123.9749
247	3092.40	3039.83	10.4341	209.1015
248	3099.92	3047.22	32.3842	121.6671
249	3114.54	3061.60	5.1713	62.3692
250	3152.92	3099.32	1.4993	108.6367
251	3554.96	3494.53	844.6780	317.9024
252	3638.08	3576.23	680.7053	306.8465
253	3718.62	3655.40	232.0727	117.3346
254	3769.45	3705.37	76.5145	167.4653
255	3779.47	3715.22	97.6888	142.0856
256	3783.79	3719.47	46.0657	116.5984
257	3790.72	3726.27	71.5733	89.9724
258	3793.62	3729.13	34.1841	147.9669
259	3804.37	3739.69	78.2534	222.5988
260	3808.03	3743.29	70.8693	116.7185
261	3814.58	3749.73	58.9606	128.9444

.

Table S9. Calculated vibrational modes frequencies and activities for the Model 3.

Mode number	Calculated mode frequency, cm^{-1}	Scaled mode frequency, cm^{-1}	IR activity, KM/Mole	Raman activity, A^4/AMU
1	8.23	8.09	0.1211	0.1139
2	11.52	11.32	0.0838	0.0410
3	13.35	13.12	0.2067	0.4008
4	17.73	17.43	0.1784	0.4262
5	23.75	23.34	0.3630	0.1798
6	26.78	26.32	0.6007	0.1387
7	29.40	28.90	1.2788	0.1301
8	35.96	35.35	0.1496	0.1311
9	40.97	40.27	1.1979	0.4498
10	48.78	47.95	0.1404	2.0687
11	50.86	49.99	1.4545	1.2119
12	53.49	52.58	3.6403	0.2400
13	61.45	60.40	0.4952	0.2910
14	65.54	64.43	3.8262	1.3807
15	71.44	70.23	4.9046	0.4620

16	77.11	75.80	1.6286	0.7690
17	84.48	83.04	6.1745	1.9243
18	88.80	87.29	4.0191	0.5194
19	92.63	91.05	5.2788	0.8020
20	95.18	93.56	1.5305	0.6090
21	103.37	101.61	3.5758	0.9801
22	115.08	113.13	1.6600	0.7179
23	120.20	118.16	2.5094	1.7762
24	122.85	120.76	2.7088	0.2236
25	137.03	134.70	1.1930	1.8113
26	150.28	147.73	0.1689	0.8045
27	153.74	151.12	10.2115	0.3610
28	156.85	154.18	2.2766	0.8290
29	157.73	155.05	7.1311	0.2503
30	167.79	164.94	5.4918	0.5021
31	177.36	174.35	0.2589	0.3624
32	182.80	179.70	0.6708	0.8109
33	192.31	189.04	1.9516	0.7161
34	202.77	199.32	2.4770	0.4961
35	210.96	207.37	1.1187	0.9387
36	216.38	212.70	20.9664	1.2505
37	220.71	216.96	5.9213	0.2207
38	235.82	231.81	3.1796	0.6205
39	243.86	239.72	7.1039	1.3015
40	249.20	244.97	111.4286	2.4096
41	259.89	255.47	8.1384	0.8073
42	267.63	263.08	19.1943	3.7444
43	276.35	271.65	177.6465	3.0680
44	282.08	277.29	2.0654	1.6538
45	286.76	281.89	45.8821	2.2417
46	286.98	282.10	27.8635	1.5745
47	287.73	282.84	77.5580	3.4591
48	291.08	286.14	10.5547	1.5517
49	300.89	295.78	48.0112	1.1407
50	302.33	297.19	69.8721	0.8939
51	310.80	305.52	94.6618	3.1261
52	314.22	308.88	50.1230	2.1257
53	320.32	314.87	22.7749	1.8330
54	324.32	318.81	248.5626	3.2835
55	325.41	319.88	18.8174	1.9717
56	332.60	326.95	56.8726	2.7448
57	335.83	330.12	36.7815	5.5452
58	340.11	334.33	211.9890	3.3968
59	345.63	339.76	6.4357	6.7287
60	349.31	343.38	59.2850	1.4334
61	350.17	344.21	36.0488	3.6065
62	366.24	360.01	30.8264	1.0460
63	372.55	366.22	19.8080	11.6146
64	387.12	380.54	39.2363	6.4508
65	393.18	386.50	9.2875	1.9120
66	400.85	394.04	14.3343	1.5666

67	405.02	398.13	203.3845	5.2032
68	408.59	401.65	10.4536	1.5445
69	423.83	416.62	9.4198	5.9985
70	429.40	422.10	43.1290	5.8440
71	431.73	424.39	5.2529	4.2514
72	438.77	431.31	21.1397	5.8556
73	444.06	436.51	2.3508	8.7726
74	453.35	445.64	13.3161	3.5238
75	467.22	459.28	4.2976	1.8256
76	477.15	469.03	7.4651	6.3760
77	484.59	476.35	21.0478	1.6146
78	490.50	482.16	153.6703	4.8923
79	499.00	490.52	10.5286	2.1034
80	503.80	495.23	10.1944	3.9723
81	517.91	509.10	7.5918	1.8405
82	550.67	541.31	23.5881	1.6532
83	555.58	546.14	10.1621	1.4001
84	557.01	547.54	96.3674	4.1082
85	571.09	561.38	132.9778	5.4564
86	577.46	567.64	31.5733	4.5501
87	590.81	580.77	11.3701	1.5938
88	593.89	583.80	39.3629	2.8729
89	606.05	595.75	19.5611	6.3326
90	612.69	602.28	73.0166	8.7725
91	613.63	603.20	12.4630	3.6931
92	633.20	622.43	50.0137	3.0052
93	634.77	623.98	4.8659	6.0211
94	642.25	631.33	43.4826	3.9493
95	648.56	637.54	28.2512	2.1456
96	665.61	654.29	17.3446	0.4300
97	676.89	665.38	64.6315	17.8553
98	699.10	687.21	17.7668	1.2005
99	704.54	692.57	43.2485	8.5907
100	709.35	697.29	137.4588	0.7566
101	727.24	714.87	4.6351	1.3435
102	764.13	751.14	26.5804	4.6485
103	774.81	761.64	27.2127	0.3013
104	795.66	782.13	60.2696	6.6123
105	848.96	834.53	271.1808	15.2227
106	855.76	841.21	357.7846	25.5509
107	898.64	883.37	3.2466	2.1569
108	907.93	892.49	6.5295	7.2116
109	915.24	899.68	30.9513	1.3530
110	919.83	904.19	4.8377	2.3107
111	934.78	918.89	6.3007	5.8261
112	968.38	951.91	12.7709	3.5242
113	982.36	965.66	173.5148	4.3152
114	983.78	967.06	131.9807	4.5540
115	990.49	973.65	41.8624	1.0014
116	995.31	978.39	210.9275	5.3109
117	999.24	982.25	108.1461	1.4852

118	1001.52	984.50	20.9285	2.7938
119	1003.78	986.71	116.1873	1.0752
120	1016.36	999.08	2.9022	2.6708
121	1025.92	1008.48	421.5867	4.9902
122	1029.29	1011.79	29.2264	1.2001
123	1033.47	1015.90	94.5816	4.5981
124	1036.84	1019.22	394.6398	11.7155
125	1039.88	1022.20	177.2527	4.6243
126	1048.43	1030.61	315.5858	4.2108
127	1050.56	1032.70	241.7740	2.0892
128	1057.49	1039.52	380.4860	6.8226
129	1059.24	1041.24	231.3851	5.4555
130	1064.18	1046.09	87.4746	5.5682
131	1065.63	1047.51	452.9281	2.2690
132	1067.97	1049.81	42.4490	10.3390
133	1068.69	1050.52	306.6711	2.6731
134	1070.57	1052.37	175.1358	8.5715
135	1073.95	1055.69	137.9130	16.9281
136	1085.02	1066.57	239.6030	4.9937
137	1094.62	1076.01	5.2635	7.8852
138	1098.07	1079.40	59.9129	11.6851
139	1103.79	1085.03	196.5957	7.5888
140	1105.11	1086.32	79.6777	20.3001
141	1107.95	1089.11	129.6218	12.6656
142	1109.22	1090.36	88.2979	6.9849
143	1114.24	1095.29	66.2447	11.1784
144	1118.18	1099.17	10.5752	10.5839
145	1119.93	1100.90	21.3529	15.7781
146	1123.17	1104.07	187.2081	1.3163
147	1124.76	1105.64	65.3951	23.9966
148	1126.09	1106.95	37.0467	1.5556
149	1130.99	1111.77	34.8253	6.0714
150	1136.56	1117.24	31.0924	3.9484
151	1139.21	1119.84	43.5271	8.8414
152	1146.60	1127.11	8.7398	9.9498
153	1148.26	1128.74	15.5422	6.5458
154	1152.18	1132.59	98.8565	16.0203
155	1165.00	1145.19	193.5478	7.9615
156	1178.18	1158.15	4.4490	6.4498
157	1179.50	1159.45	139.0459	6.1002
158	1202.11	1181.68	24.0580	3.5658
159	1203.72	1183.25	1.4677	10.3991
160	1206.83	1186.31	72.3122	3.0796
161	1219.15	1198.42	58.8028	0.8698
162	1226.95	1206.09	47.1650	3.3725
163	1232.21	1211.26	49.1204	7.9607
164	1237.48	1216.44	6.1546	8.3792
165	1238.78	1217.72	26.9172	3.1822
166	1263.06	1241.59	2.6680	5.9534
167	1269.25	1247.67	25.8526	7.2662
168	1271.77	1250.15	22.5161	4.5251

169	1273.74	1252.09	67.3628	7.2121
170	1274.82	1253.15	22.4602	2.6537
171	1292.99	1271.01	12.5086	4.2327
172	1299.83	1277.74	34.3470	1.1061
173	1305.11	1282.92	68.5730	8.9650
174	1307.06	1284.84	79.7562	6.5552
175	1310.39	1288.12	17.4676	16.4252
176	1311.55	1289.25	330.0915	23.6196
177	1314.62	1292.27	31.5221	6.7887
178	1319.71	1297.28	9.8455	15.8767
179	1323.20	1300.70	16.6427	7.9119
180	1323.68	1301.18	33.5210	15.2247
181	1335.67	1312.97	21.9996	5.7344
182	1335.95	1313.24	23.9598	8.7277
183	1341.14	1318.34	24.2779	5.1175
184	1348.37	1325.45	41.2785	13.5589
185	1350.10	1327.15	104.9335	15.6604
186	1352.82	1329.82	8.0392	9.7579
187	1357.89	1334.80	15.5813	12.0556
188	1359.14	1336.04	34.2705	20.5127
189	1367.51	1344.26	12.0767	9.3760
190	1374.22	1350.86	36.7143	7.3942
191	1379.67	1356.21	25.2218	2.6057
192	1383.13	1359.62	38.9905	4.4894
193	1387.50	1363.91	11.4978	9.2116
194	1389.30	1365.68	35.2165	9.3830
195	1390.49	1366.85	23.2214	9.6419
196	1392.83	1369.15	11.9766	15.3687
197	1397.11	1373.36	2.2626	22.0343
198	1399.02	1375.24	16.2727	17.3543
199	1402.29	1378.45	21.7695	3.3808
200	1405.89	1381.99	8.7456	22.4947
201	1408.23	1384.29	8.0063	3.5547
202	1414.88	1390.83	4.1136	12.3040
203	1419.13	1395.00	114.5744	12.7622
204	1420.30	1396.15	62.9642	11.2802
205	1421.24	1397.08	13.1281	6.0580
206	1423.37	1399.17	63.0995	5.3731
207	1425.30	1401.07	42.8376	4.0880
208	1428.26	1403.98	42.8705	6.5496
209	1434.38	1409.99	6.3626	12.6891
210	1435.00	1410.61	56.0838	15.5701
211	1444.54	1419.98	11.5061	9.4199
212	1444.92	1420.36	32.4382	0.7647
213	1460.56	1435.73	26.8241	4.0030
214	1464.17	1439.28	101.2940	11.0883
215	1469.36	1444.38	61.2200	4.2955
216	1472.90	1447.86	30.8959	15.7127
217	1475.32	1450.24	5.7926	6.6406
218	1503.77	1478.21	2.6020	13.9612
219	1505.15	1479.56	3.4703	12.2186

220	1512.59	1486.88	0.9173	13.5309
221	1678.42	1649.89	656.5990	3.7916
222	1687.49	1658.80	712.8721	6.0775
223	1804.64	1773.96	247.9647	38.4215
224	2951.10	2900.93	81.8601	197.5515
225	2958.24	2907.95	19.5250	80.4932
226	2966.52	2916.09	4.3319	7.2245
227	2966.72	2916.29	83.7417	248.6756
228	2970.96	2920.45	50.7731	255.6102
229	2973.34	2922.79	68.8572	236.9294
230	2974.99	2924.41	69.0441	642.2556
231	2985.82	2935.06	41.9690	347.9108
232	2993.57	2942.68	68.4903	70.8181
233	3001.78	2950.75	119.4264	23.1638
234	3009.57	2958.41	29.7420	142.6061
235	3018.59	2967.27	69.3526	292.4566
236	3023.74	2972.34	2.4051	295.4970
237	3027.69	2976.21	41.9529	58.7255
238	3031.19	2979.66	2.4498	276.4033
239	3035.25	2983.65	63.9060	304.2860
240	3037.07	2985.44	33.1595	18.0649
241	3045.70	2993.92	92.9271	23.2843
242	3050.80	2998.94	55.5782	163.7585
243	3063.27	3011.20	45.3073	173.1025
244	3066.37	3014.24	3.7898	230.4321
245	3076.90	3024.60	54.8693	202.0142
246	3082.98	3030.57	47.2131	124.5459
247	3091.78	3039.21	11.7729	208.9178
248	3098.08	3045.41	34.0407	120.8288
249	3103.59	3050.83	13.3078	54.4899
250	3150.72	3097.15	2.2010	107.5216
251	3556.27	3495.81	833.6392	317.1180
252	3639.34	3577.47	675.4776	305.3956
253	3719.15	3655.93	232.2569	116.8850
254	3769.05	3704.97	75.7495	166.8575
255	3781.81	3717.52	92.7312	138.4983
256	3784.32	3719.99	42.4547	111.9626
257	3790.17	3725.73	66.6998	97.6322
258	3794.23	3729.72	36.3994	135.4824
259	3802.38	3737.74	80.3325	226.4145
260	3805.56	3740.86	72.5318	119.5173
261	3812.22	3747.41	55.6987	125.9416

Table S10. Calculated vibrational modes frequencies and activities for the Model 4.

Mode number	Calculated mode frequency, cm^{-1}	Scaled mode frequency, cm^{-1}	IR activity, KM/Mole	Raman activity, A^4/AMU
1	7.19	7.07	0.0932	0.1987
2	9.79	9.62	0.1587	0.1609
3	16.44	16.16	0.2538	0.2301
4	23.42	23.02	0.1420	0.2667

5	25.93	25.49	0.3906	0.1433
6	28.22	27.74	0.6299	0.1403
7	32.73	32.18	2.1183	0.1517
8	36.64	36.02	0.1547	0.2504
9	46.40	45.61	2.4071	0.1950
10	52.36	51.47	0.1734	1.1799
11	56.72	55.75	0.8587	2.4953
12	61.12	60.08	2.6013	0.2492
13	67.00	65.87	8.2544	2.9378
14	68.06	66.90	1.5861	0.5377
15	72.06	70.83	0.8458	1.1335
16	78.31	76.98	1.2591	0.4572
17	87.52	86.03	2.7462	0.7654
18	91.73	90.17	1.6409	0.7040
19	93.85	92.25	2.8343	0.4803
20	99.31	97.62	3.2318	0.6775
21	105.73	103.93	0.8989	4.7902
22	107.14	105.32	1.7489	0.4958
23	115.04	113.09	4.5020	0.4706
24	123.29	121.20	0.2241	1.3210
25	126.34	124.20	7.0625	0.7435
26	138.87	136.51	1.4588	0.8736
27	151.41	148.84	0.3044	0.5068
28	152.18	149.59	7.5390	0.2564
29	156.26	153.61	5.1497	0.4251
30	162.13	159.38	0.3848	0.3518
31	178.37	175.34	3.8923	0.4004
32	194.83	191.52	0.5981	1.3519
33	200.49	197.08	14.8865	0.3577
34	202.73	199.29	8.0261	0.9879
35	207.43	203.90	7.8162	0.6994
36	221.09	217.33	8.5897	0.2964
37	234.48	230.49	5.3958	0.8242
38	241.24	237.14	144.4185	5.7775
39	244.86	240.70	3.4430	1.4313
40	256.35	252.00	42.3303	0.7265
41	262.94	258.47	71.2334	0.9738
42	273.52	268.87	98.6935	2.2503
43	275.45	270.77	29.9291	0.9913
44	277.70	272.98	12.7361	1.7775
45	281.40	276.62	61.5090	1.3400
46	284.55	279.71	21.6269	3.3981
47	292.00	287.04	14.1277	2.5412
48	295.80	290.77	108.6741	1.6160
49	298.57	293.50	1.4580	0.1835
50	304.95	299.76	33.6135	2.0772
51	309.79	304.53	173.9281	1.4875
52	316.39	311.01	112.3667	0.8387
53	320.89	315.44	22.1830	1.1090
54	328.69	323.10	126.6369	4.9015
55	329.71	324.10	52.8180	5.1917

56	335.36	329.66	142.5715	1.1583
57	336.31	330.59	17.0824	5.6366
58	345.82	339.95	4.2024	8.7086
59	351.11	345.14	21.8363	5.2697
60	358.85	352.75	72.9950	1.0997
61	371.33	365.02	2.1314	4.4740
62	379.39	372.94	23.2320	6.9975
63	394.74	388.03	16.0054	4.4294
64	397.95	391.18	12.5814	1.5732
65	402.35	395.51	9.7561	3.4965
66	404.96	398.07	14.3272	7.0128
67	411.67	404.67	178.6453	4.7347
68	430.23	422.92	51.8941	7.8987
69	432.12	424.78	16.2542	3.4057
70	433.78	426.40	11.9964	0.9612
71	437.11	429.68	7.9569	7.9619
72	447.35	439.74	3.3457	1.9953
73	452.56	444.86	3.0357	10.4290
74	476.29	468.19	5.4384	5.1978
75	482.71	474.50	151.5610	6.6602
76	498.27	489.80	50.3957	0.4746
77	503.53	494.97	44.6556	2.2927
78	518.95	510.13	5.2505	4.3982
79	543.40	534.16	11.4376	3.1523
80	553.91	544.49	40.7312	1.9831
81	556.07	546.61	142.4832	2.6153
82	563.35	553.77	15.9884	2.3208
83	568.83	559.16	10.5013	4.0311
84	571.55	561.84	56.2556	3.8810
85	586.45	576.48	59.2464	2.6116
86	590.25	580.22	58.0137	9.6813
87	604.11	593.84	41.6433	10.5763
88	606.97	596.66	18.0558	3.0457
89	617.39	606.89	12.7219	2.9071
90	624.83	614.21	36.1150	3.5641
91	637.40	626.56	35.3704	2.0769
92	645.94	634.96	1.2852	0.9948
93	652.99	641.89	85.7139	2.0562
94	664.52	653.22	9.1310	1.1228
95	683.24	671.62	25.7339	4.4462
96	703.78	691.82	27.7402	0.4359
97	705.67	693.67	144.9710	0.7262
98	728.59	716.20	25.8150	10.5201
99	743.32	730.69	17.3443	4.4555
100	770.35	757.25	33.9877	1.3141
101	774.91	761.74	30.0320	0.6943
102	817.70	803.80	139.2704	2.9741
103	844.36	830.01	368.3001	11.9055
104	853.19	838.69	199.3187	26.2638
105	899.74	884.45	3.3535	2.1575
106	914.32	898.78	31.0584	6.2012

107	918.19	902.58	48.5953	2.6329
108	919.54	903.91	13.0565	2.1820
109	949.45	933.31	98.8720	4.1436
110	968.17	951.71	25.8582	3.0636
111	982.61	965.90	131.4328	2.6596
112	984.69	967.95	56.9430	1.3458
113	986.73	969.95	530.0041	2.4312
114	995.95	979.02	275.0430	5.4639
115	998.76	981.78	155.4486	1.7259
116	999.69	982.70	105.1173	4.3519
117	1004.53	987.46	76.1769	0.9465
118	1020.02	1002.68	138.9734	5.3279
119	1027.82	1010.35	238.8807	2.5162
120	1031.19	1013.66	58.1628	8.2412
121	1033.93	1016.35	38.7865	1.8173
122	1039.31	1021.64	509.6147	2.5693
123	1048.72	1030.90	63.3017	4.2518
124	1048.96	1031.13	642.0597	7.3048
125	1056.23	1038.28	324.3460	3.8714
126	1061.67	1043.62	438.9491	1.8359
127	1063.10	1045.03	46.6536	10.0901
128	1066.90	1048.76	346.4040	3.5747
129	1068.12	1049.96	58.4521	7.2222
130	1070.96	1052.75	39.8617	1.7380
131	1073.89	1055.64	111.3210	15.2436
132	1089.84	1071.31	159.0395	6.1759
133	1095.21	1076.59	42.7749	19.4822
134	1099.92	1081.22	170.5949	12.4574
135	1103.65	1084.89	48.7343	7.2584
136	1105.99	1087.19	77.7508	28.4202
137	1108.04	1089.20	225.9947	10.7819
138	1112.55	1093.63	90.6985	6.8671
139	1114.37	1095.43	78.2594	13.9082
140	1117.13	1098.14	26.4162	10.3429
141	1120.04	1101.00	23.9643	11.0180
142	1127.04	1107.88	66.0275	2.4896
143	1128.69	1109.51	11.6422	3.6457
144	1132.44	1113.18	38.7948	11.8130
145	1135.75	1116.44	1.5240	4.8389
146	1139.27	1119.90	11.5203	7.1047
147	1143.36	1123.93	462.3382	1.5634
148	1148.02	1128.51	125.0869	10.7730
149	1157.88	1138.19	128.1237	11.6726
150	1161.50	1141.75	64.3298	15.7757
151	1171.87	1151.95	26.3552	2.3648
152	1178.73	1158.69	49.6053	8.3201
153	1201.14	1180.72	16.8812	2.2815
154	1203.72	1183.25	83.7411	3.4619
155	1205.06	1184.58	0.4712	10.7092
156	1209.50	1188.94	62.7640	2.7347
157	1220.45	1199.70	32.8483	0.8408

158	1227.74	1206.86	42.5118	3.0573
159	1232.24	1211.29	45.5028	9.8979
160	1237.65	1216.61	10.4289	6.2253
161	1247.14	1225.94	50.1726	1.4019
162	1271.23	1249.62	37.9967	2.8690
163	1275.33	1253.65	45.8402	5.4373
164	1275.43	1253.75	22.7630	5.7315
165	1275.64	1253.96	17.1495	1.5151
166	1294.64	1272.63	18.5497	5.7610
167	1300.53	1278.42	10.6612	5.9157
168	1301.51	1279.38	36.9847	1.1632
169	1310.98	1288.70	164.1035	8.9294
170	1313.81	1291.48	68.9283	5.6873
171	1314.56	1292.21	141.2678	4.9774
172	1319.32	1296.89	21.9207	17.5000
173	1322.56	1300.07	31.8743	11.3372
174	1324.44	1301.92	145.0065	15.2440
175	1337.79	1315.05	7.1316	6.2998
176	1339.01	1316.25	16.4251	3.1734
177	1340.86	1318.07	46.8998	7.3574
178	1341.82	1319.00	65.0979	9.6689
179	1347.59	1324.68	21.2631	24.4969
180	1353.67	1330.65	13.4338	6.1985
181	1360.21	1337.08	156.2954	7.2464
182	1362.01	1338.85	8.4234	7.8913
183	1366.27	1343.04	1.1635	9.8796
184	1370.47	1347.17	14.4270	12.9583
185	1382.28	1358.78	28.5550	6.7256
186	1385.65	1362.09	27.4928	5.8948
187	1392.27	1368.61	11.2110	23.8918
188	1393.99	1370.29	17.1301	12.6115
189	1395.93	1372.20	13.1011	14.7649
190	1397.75	1373.99	4.3687	15.5332
191	1401.00	1377.19	55.3853	20.1456
192	1402.71	1378.86	41.2160	6.0871
193	1406.48	1382.57	0.7721	3.3744
194	1408.99	1385.04	7.6485	20.1565
195	1414.88	1390.83	21.1094	12.7085
196	1420.10	1395.96	52.4083	8.4068
197	1421.31	1397.14	71.2281	5.9527
198	1422.32	1398.14	27.6211	4.1424
199	1422.76	1398.57	80.3323	5.0951
200	1427.92	1403.64	17.1123	5.3734
201	1429.39	1405.09	28.6895	10.6682
202	1430.63	1406.31	58.4569	6.8724
203	1437.01	1412.59	12.8120	7.9904
204	1441.97	1417.46	10.8955	14.7660
205	1445.72	1421.14	31.1813	11.9192
206	1446.67	1422.08	3.0339	8.9769
207	1460.75	1435.91	22.8313	3.4284
208	1463.93	1439.04	103.3460	10.8761

209	1470.14	1445.15	59.8724	2.9789
210	1474.87	1449.80	4.2837	6.0730
211	1502.58	1477.03	2.9453	14.1820
212	1507.41	1481.79	2.6910	9.9896
213	1513.49	1487.76	0.8320	13.2185
214	1684.38	1655.75	649.6806	6.3542
215	1690.58	1661.84	790.6533	5.3009
216	1799.04	1768.45	508.1227	39.2328
217	2954.27	2904.05	49.0499	169.9381
218	2954.86	2904.63	87.3736	193.2400
219	2968.90	2918.43	18.2885	126.5974
220	2971.28	2920.77	51.7905	230.1901
221	2976.57	2925.97	32.7321	130.8871
222	2977.61	2926.99	43.4878	471.8794
223	2979.09	2928.45	94.2579	296.1060
224	2986.10	2935.34	48.1824	319.0728
225	2992.03	2941.17	52.1551	88.2574
226	3002.62	2951.58	130.5323	24.1808
227	3019.57	2968.24	56.4893	230.2295
228	3024.90	2973.48	14.2724	420.8903
229	3031.40	2979.87	40.7082	65.6262
230	3031.48	2979.94	4.6279	101.4969
231	3038.75	2987.09	56.2512	292.0651
232	3045.76	2993.98	108.7005	24.5436
233	3049.70	2997.86	56.4636	159.5102
234	3066.29	3014.16	38.8345	168.6345
235	3076.96	3024.66	54.4384	202.8614
236	3077.02	3024.71	12.4902	136.1737
237	3085.57	3033.12	45.1572	122.1621
238	3099.78	3047.09	31.0538	118.0694
239	3111.80	3058.90	0.4189	262.6279
240	3123.88	3070.77	8.2261	22.1209
241	3137.94	3084.59	5.9414	45.7678
242	3558.58	3498.08	829.1328	316.5160
243	3636.31	3574.50	684.8930	301.4658
244	3718.99	3655.77	231.9500	117.4269
245	3769.52	3705.44	76.1438	166.8284
246	3784.16	3719.83	44.8223	114.6447
247	3790.19	3725.76	74.9697	69.2822
248	3792.02	3727.55	29.1908	164.6430
249	3792.30	3727.83	49.5593	122.7494
250	3803.60	3738.94	81.0062	220.5966
251	3807.28	3742.56	72.2169	119.9544
252	3812.33	3747.52	56.0362	127.0588

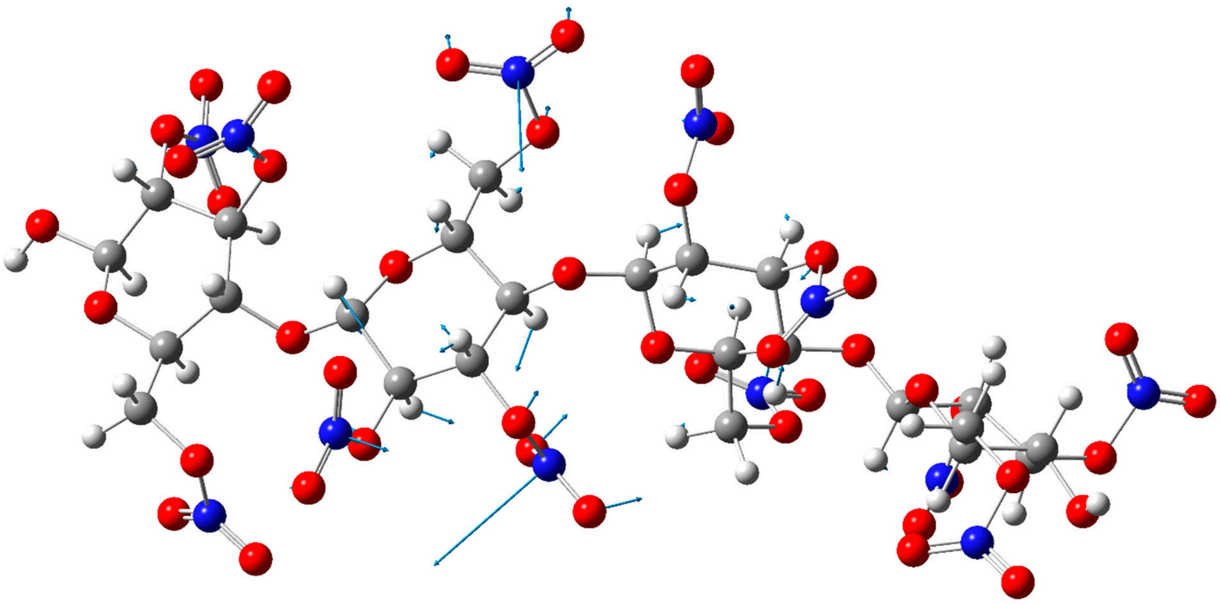

Figure S1. Atomic displacements in mode №149 in NC Model (for notations in second from the left side pyranose ring see Figure 1b).

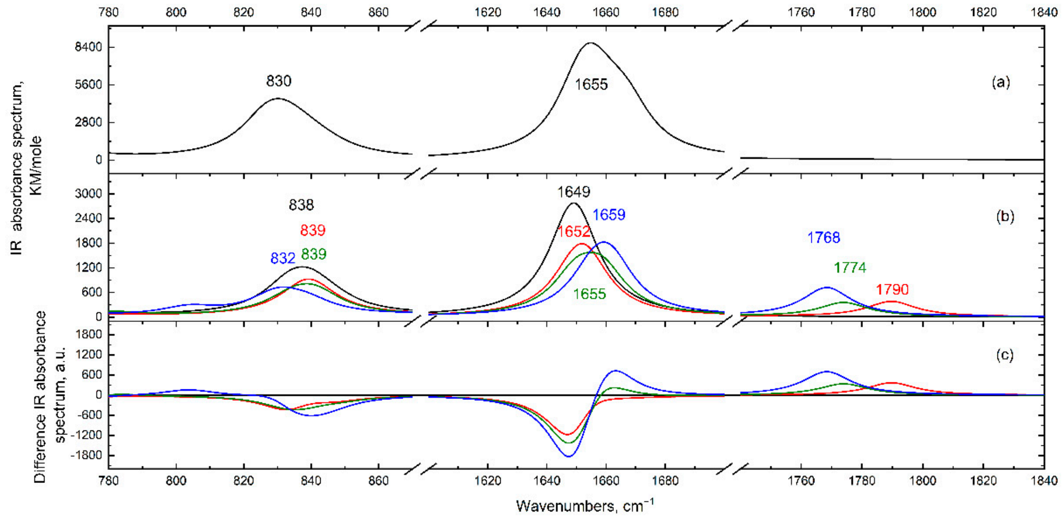

Figure S2. Calculated scaled infrared (IR) absorption spectra for: (a) the NC Model; (b) Model structures 1 (black), 2 (red), 3 (green), and 4 (blue); (c) difference scaled IR absorption spectra, obtained by sequentially subtracting the scaled IR absorption spectrum of Model 1 from the scaled spectra of Model 2 (red), Model 3 (green), and Model 4 (blue) in the selected regions. The position of peak maximum for each Model is highlighted with the same color.

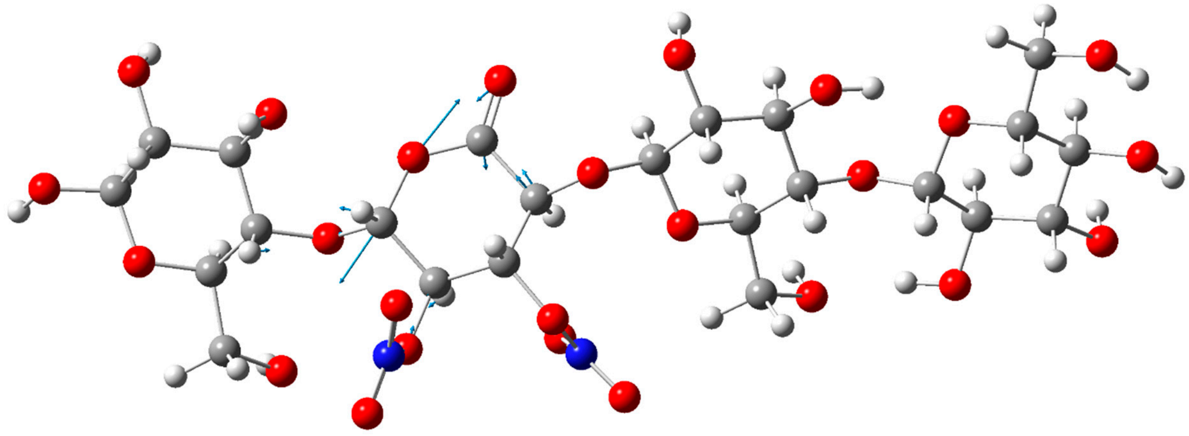

Figure S3. Atomic displacements in mode №113 in Model 4 (for notations in second from the left side pyranose ring see Figure 1b).

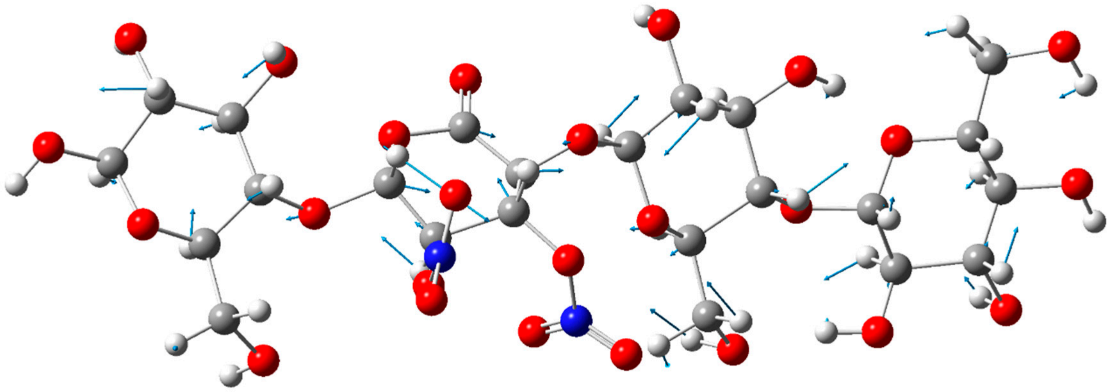

Figure S4. Atomic displacements in mode №147 in Model 4 (for notations in second from the left side pyranose ring see Figure 1b).

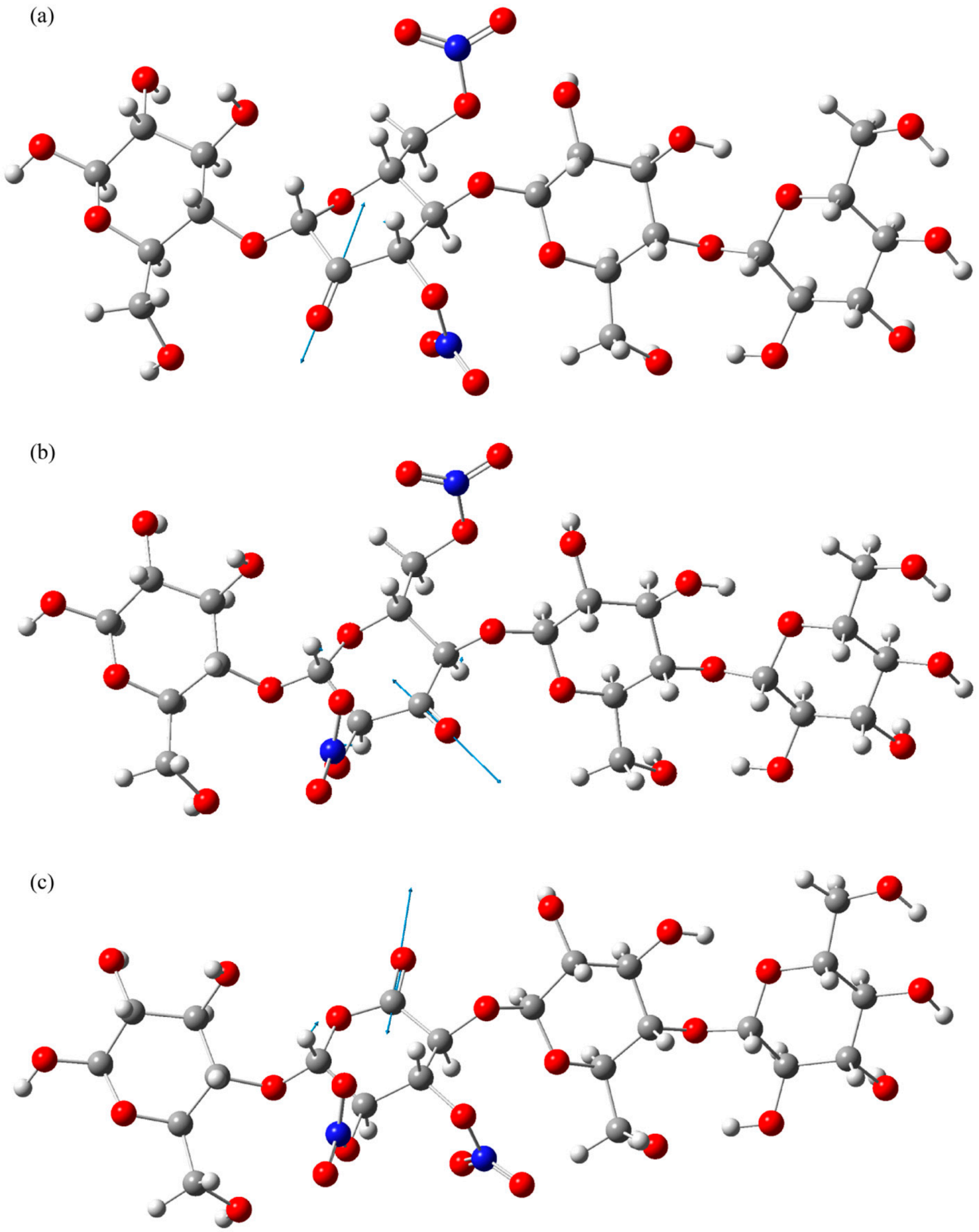

Figure S5. Atomic displacements in modes with predominant ν(C=O) atomic displacements in Model 2 (a), Model 3 (c) and Model 4 (d).

Vibrational properties test

Number of pyranose rings

It was verified the convergence of the predicted vibrational properties using Model structures with varying numbers of pyranose units. For this purpose, the spectra were preliminarily normalized by the peak area at 830 cm^{-1} (IR absorbance spectra) and 844 cm^{-1} (Raman spectra); the frequencies are scaled. The C=O stretching band in the IR absorbance spectra exhibited the highest sensitivity to the number of pyranose rings, with changes in absorption intensity but no shift in the peak maximum. Although our calculations somewhat overestimated the frequency of this band-likely due to underestimation of hydrogen-bonding effects from the medium - this overestimation is systematic, as indicated by the consistent peak positions across structures with 2 to 5 rings. Thus, reliable trend analysis remains feasible, including relative frequency shifts (in cm^{-1}) between Model structures.

In IR absorbance spectrum the band contour differences near 1020 cm^{-1} are associated with variations in the C-O-C bond parameters at the pyranose ring boundaries (bond lengths, flat angles, and dihedral angles) as the chain length increases (Figure S6). These structural changes are also visually evident in the differing dihedral angles presented in Figure S7, which govern the relative orientation of the rings.

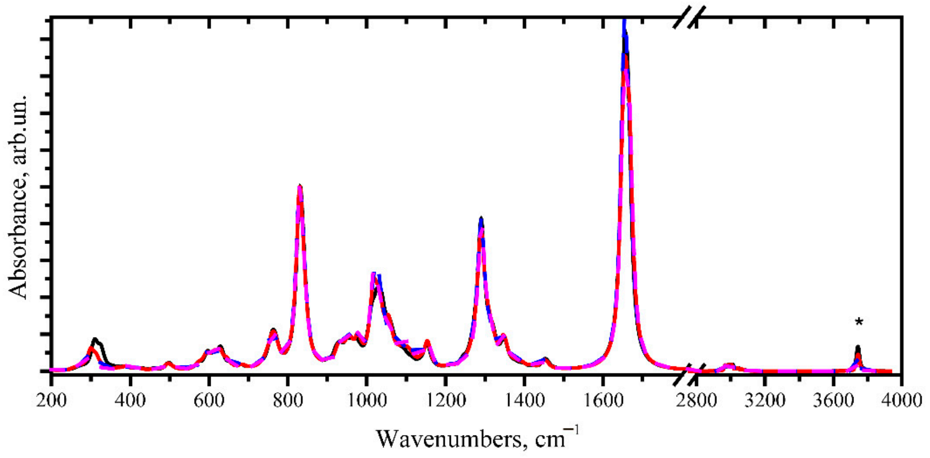

Figure S6. Calculated IR absorbance spectra with variable number of pyranose rings in the Model structure: 2(black), 3(red), 4(blue) and 5 (pink) normalized by 830cm^{-1} peak area.

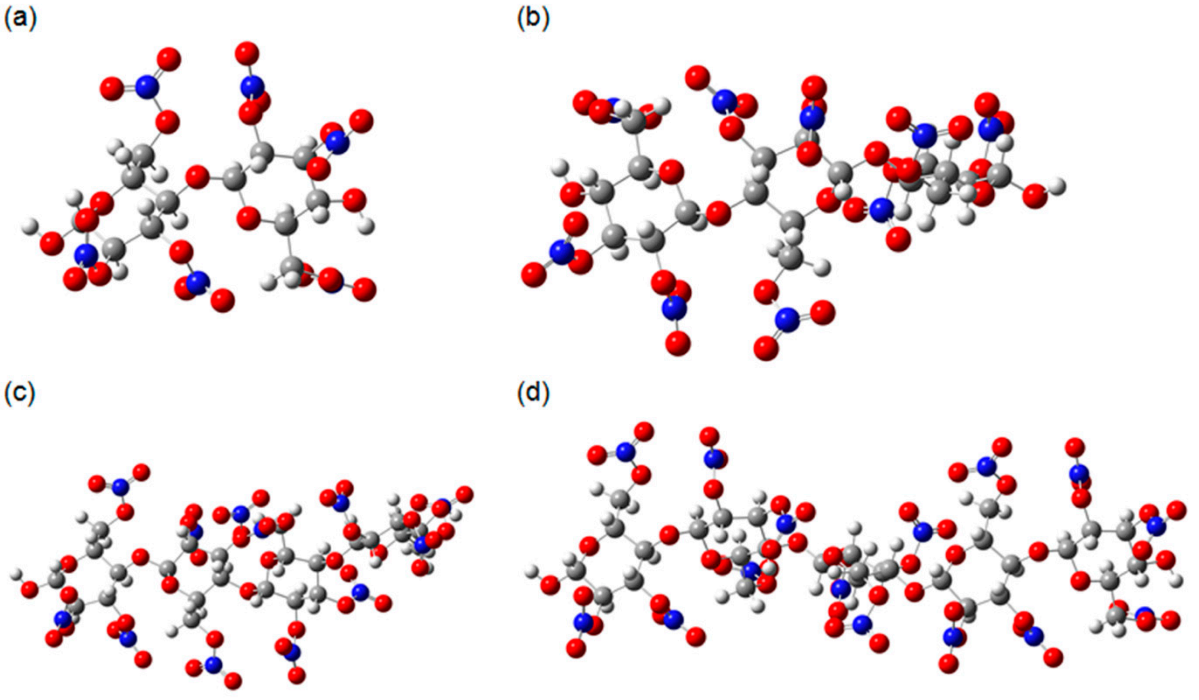

Figure S7. Structure of the Models with 2(a), 3(b), 4(c) and 5(d). Red, blue, light grey and dark grey colors corresponds to the oxygen, nitrogen, hydrogen and carbon.

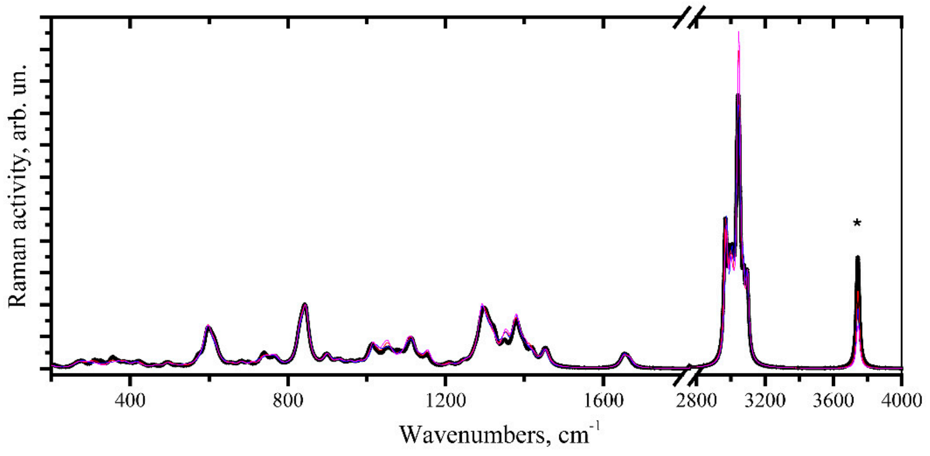

Figure S8. Calculated Raman activity with variable number of pyranose rings in the Model structure: 2(black), 3(red), 4(blue) and 5 (pink) normalized by 844 cm^{-1} peak area.

In the Raman activity spectra (Figure S8) for Models with an increasing number of pyranose rings (from 2 to 5), the peaks attributed to NO_2 groups appear in close proximity to one another. However, notable differences were observed for the peaks at 1354, 1049, and 1148 cm^{-1}. The band at 1354 cm^{-1} arises from deformation vibrations involving hydrogen atoms, specifically of the type δ(XCH) (where X=C or O), accompanied by relatively minor changes in the carbon–hydrogen stretching contours.

The peak at 1049 cm^{-1} originates from ν(COC) stretching vibrations within the pyranose ring, whereas the peak at 1148 cm^{-1} contains contributions from ν(COC) stretching vibrations at the inter-ring boundaries. This latter observation is complementary to the trends noted in the IR absorption spectra.

Local environment influence

To assess the influence of the local environment, we additionally modeled the interacting portions of the chains (Figure S9). The interaction effect was deliberately maximized by selecting relatively small structures, since in larger systems the probability of intermolecular contacts diminishes due to the chain rigidity and the steric constraints that impede hydrogen–oxygen interactions.

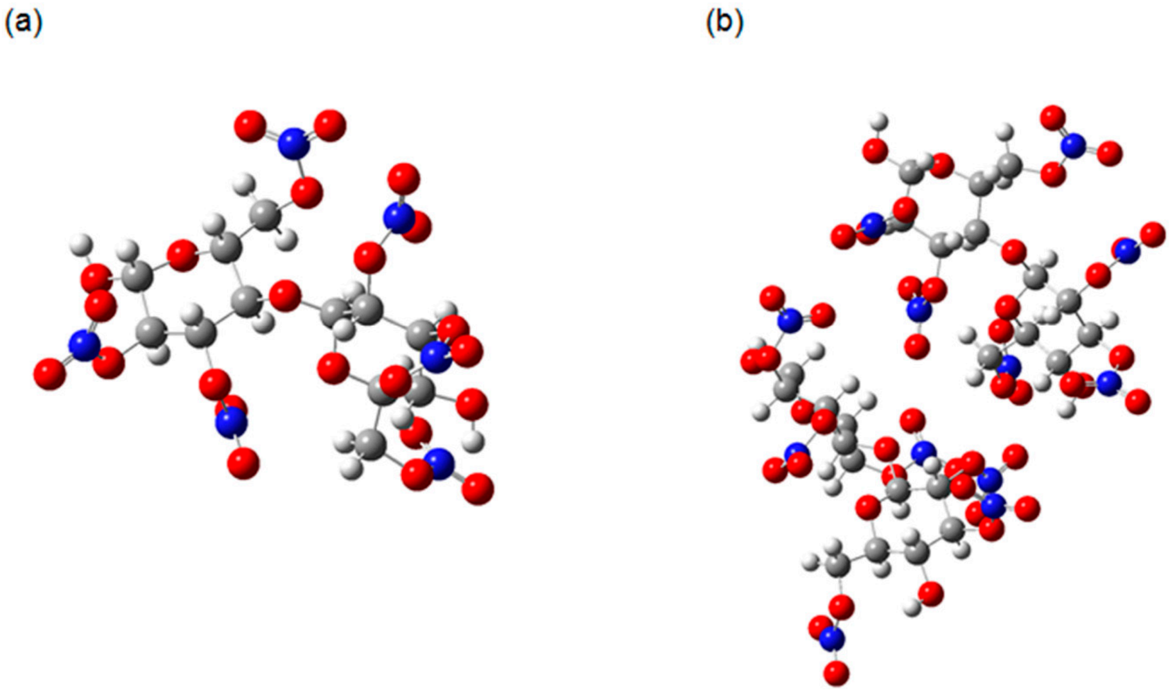

Figure S9. The optimized geometries of the 2 units structure (a) and 2+2=4 units structure (b).

As shown in Figures S10 and S11, the interaction exerts a relatively minor effect on the vibrational peak contours and frequencies overall within the current approach. We attribute this behavior to two main factors: pronounced intra-chain interactions and the maximum degree of substitution by nitrate (NO_3) groups.

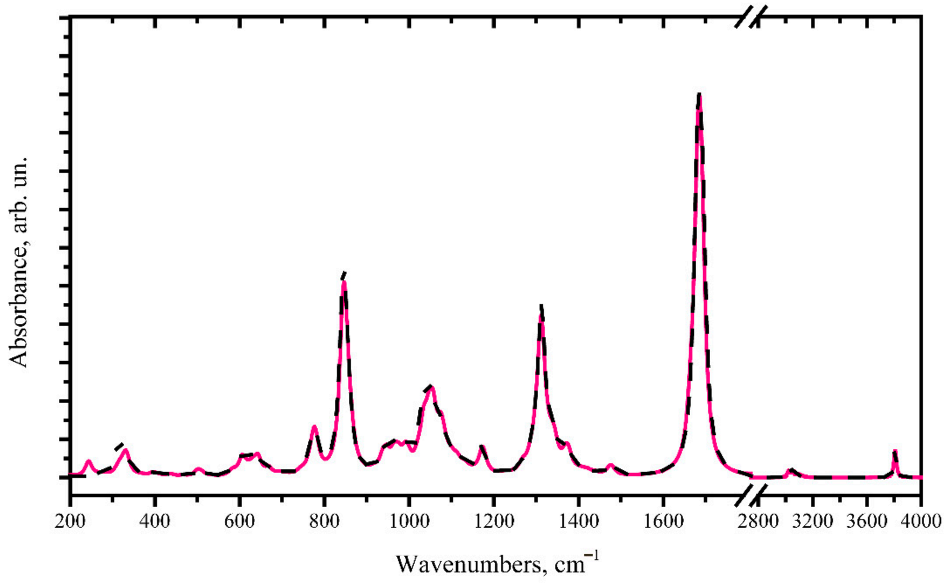

Figure S10. a – Optimized structure of 2 pyranose ring units; b -; c - calculated IR absorbance spectra with 2 pyranose ring units (black solids) and 2+2=4 pyranose ring units (pink dashed).

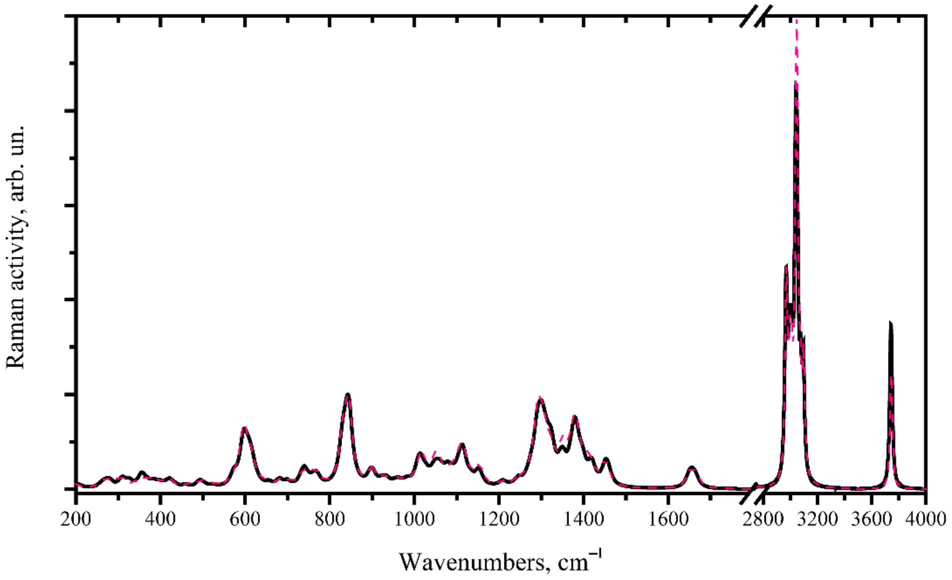

Figure S11. a – Optimized structure of 2 pyranose ring units; b – calculated scaled IR absorbance spectra with 2 pyranose ring units (black solids) and 2+2=4 pyranose ring units (pink dashed).

Comparison of the calculated spectra for the Models with 2 and 2+2 pyranose rings shows that accounting for hydrogen-bonding interactions has a relatively minor effect on the spectral contours. The most notable difference was observed in the Raman activity spectrum near 1343 cm^{-1}, where vibrational modes associated with deformation vibrations of the type δ(XCH) (X = C or O) are manifested. In this case, one of the hydrogen atoms is in contact with an oxygen atom of a nitrate group belonging to the other 2-pyranose-ring unit (Figure S12a). Additionally, a sharp increase in Raman activity was noted for the vibrational mode with a scaled frequency of 3055 cm^{-1}, in which a hydro-

gen atom is in contact with an oxygen atom of a nitrate group belonging to the other 2-pyranose-ring unit (Figure S12b).

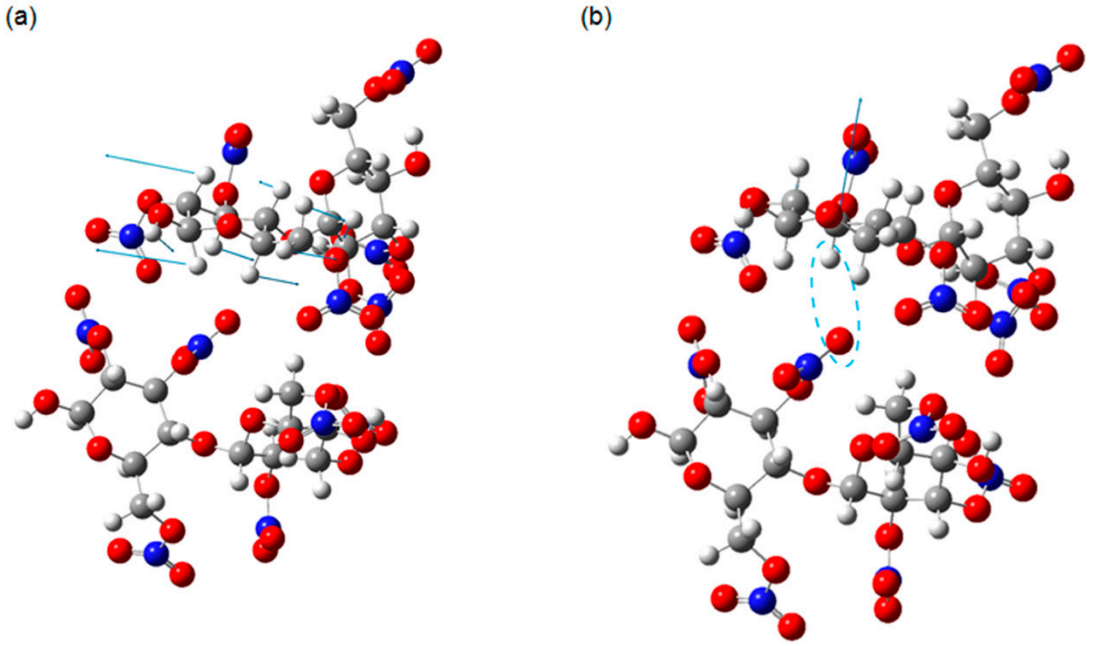

Figure S12. Hydrogen bending vibrational mode with scaled frequency 1343 cm^{-1} (a) and hydrogen-carbon stretching vibrational mode with scaled frequency 3055 cm^{-1} (b).

Thus, accounting for interchain interactions leads to an increase in the relative activity (intensity), as predicted by the calculations, for vibrational modes involving atomic displacements of hydrogen atoms that are in contact with oxygen atoms of another chain. Given the relatively small number of such contacts in the modelling structures, these interchain interactions are presumably expected to exert only a weak influence on the vibrational spectrum. Additionally, a slight effect may also be observed on the absorption of vibrational modes in which carbon atoms are adjacent to such hydrogen atoms. In the present case, this manifests as a minor change in the Raman activity in the region around 1054 cm^{-1}, which corresponds to stretching vibrations of the C-O-C bonds within the pyranose ring. However, in the real structures due to the lower then 3 substitution coefficient and possible defects the interchain interactions need additional studies.

Table S11. Optimized geometry for the Model 5.

C	0.76204000	0.53315400	-0.22016200
C	6.00391600	0.21428800	0.36884100
H	0.89286500	1.06778000	0.73109600
C	-0.23717100	1.26675300	-1.11738000
C	5.25165600	-1.09418900	0.06211100
H	-0.22567300	0.77286000	-2.09215000
H	5.09035300	-1.54916200	1.04054100
C	-1.64488300	1.18592300	-0.53162000
C	3.90738900	-0.94425000	-0.64858900
H	-1.67894000	1.80890100	0.37684700
H	4.06449000	-0.80009500	-1.71706000
C	-1.99922000	-0.24354800	-0.10383100
C	3.14421700	0.27575600	-0.13284200
H	-2.12688700	-0.86363200	-0.99728900
H	2.90254400	0.17675300	0.92376900

C	-0.89880100	-0.84868000	0.77341900
H	-0.81073900	-0.26234800	1.69941500
C	-1.10112200	-2.32190000	1.14145800
H	-1.40346200	-2.89289600	0.26310700
H	-0.14426400	-2.71597900	1.49069600
O	0.17414200	2.60772600	-1.34773600
O	3.15210900	-2.17819200	-0.68769600
O	1.97520800	0.45504000	-0.91341500
O	0.33156600	-0.79393800	0.04420700
O	5.30734300	1.36175000	0.36548400
O	-2.11324100	-2.53868600	2.12256200
H	-0.12098400	3.15471300	-0.60851300
O	-2.54552700	1.68363100	-1.50303500
O	-3.21145400	-0.23513000	0.65757300
H	-1.85511300	-2.10257300	2.94358800
H	-3.44680800	1.59134400	-1.14737200
C	-4.40455300	-0.51332400	0.00294100
H	-4.23062000	-1.14019600	-0.88163100
O	-4.98167600	0.73347500	-0.40788700
C	-5.31659000	-1.24707500	0.99146800
C	-6.22308500	0.57210700	-1.10815900
C	-6.70893900	-1.39982900	0.38937500
O	-4.79720500	-2.52768200	1.27516600
H	-5.37455600	-0.62645400	1.89803400
H	-6.08401000	-0.08114800	-1.97940900
C	-6.65680100	1.95387600	-1.59351000
C	-7.22462600	-0.06526300	-0.14437900
H	-6.65245500	-2.10726000	-0.44302900
O	-7.66246800	-1.96922000	1.28346600
H	-3.89095600	-2.42660500	1.62718000
O	-7.77370200	1.88086400	-2.46608200
H	-5.83936600	2.40471100	-2.15890300
H	-6.86045000	2.59285700	-0.72445400
O	-8.46120200	-0.25455100	-0.82895100
H	-7.36607800	0.62463300	0.69947900
H	-7.66184200	-1.45266200	2.10027600
H	-8.43562100	1.32715800	-2.02528700
N	2.64724500	-2.69840500	0.52845700
O	2.90860900	-2.12278800	1.55784000
O	2.00334300	-3.69312200	0.35738800
H	-9.03417800	-0.75750900	-0.23351900
O	6.11474800	-2.05432500	-0.55871400
N	6.72157300	-1.62064500	-1.77820600
O	6.43992700	-0.51767300	-2.18217900
O	7.44919600	-2.44604400	-2.24092700
O	7.15215700	0.19973400	0.70801800
C	3.44117100	2.81138000	0.06775300
H	2.56614700	3.01713400	-0.54647100
H	4.17191800	3.61189700	-0.02968900
O	3.05080300	2.66882400	1.45416100
N	2.44521400	3.82121000	1.99664000
O	2.33102800	4.78532600	1.28132800
O	2.11595400	3.67375100	3.14216700

C	4.05503800	1.49420200	-0.35075200
H	4.28136800	1.58621600	-1.41624000

Table S12. Calculated vibrational modes frequencies and activities for the Model 5.

Mode number	Calculated mode frequency, cm^{-1}	Scaled mode frequency, cm^{-1}	IR activity, KM/Mole	Raman activity, A^4/AMU
1	8.18	8.04	0.4854	0.6084
2	11.73	11.53	1.2661	0.3706
3	21.48	21.11	1.2531	0.3772
4	22.64	22.25	0.2612	0.4703
5	27.36	26.90	0.4450	0.4576
6	30.37	29.85	1.7546	0.1103
7	38.21	37.56	4.3317	0.3196
8	47.00	46.20	0.1021	3.0007
9	50.30	49.44	2.0453	1.0055
10	59.29	58.28	2.1686	0.7154
11	64.61	63.51	0.3125	1.2673
12	70.25	69.05	2.3078	0.1453
13	76.36	75.06	0.6232	4.2123
14	83.97	82.54	1.8652	3.3545
15	95.39	93.77	0.7428	0.4985
16	96.36	94.73	1.6837	0.9929
17	106.60	104.79	2.7924	0.4597
18	109.01	107.15	1.8889	0.6957
19	115.19	113.23	0.4965	4.1593
20	124.04	121.93	4.4145	0.7287
21	132.62	130.37	2.3366	2.1999
22	140.56	138.18	2.4028	1.8432
23	151.73	149.15	1.6533	0.6387
24	153.53	150.92	6.7845	0.5243
25	159.58	156.87	7.6575	0.7756
26	169.65	166.77	3.8063	0.3603
27	177.17	174.16	3.4184	1.0925
28	187.83	184.64	4.0377	1.4922
29	202.39	198.95	2.7548	0.8101
30	208.60	205.05	4.4613	0.9073
31	220.76	217.01	11.0779	0.2355
32	227.52	223.65	7.6357	2.4669
33	247.54	243.33	0.3511	1.0111
34	257.88	253.49	11.9563	0.8522
35	264.56	260.07	39.4347	1.6344
36	276.32	271.63	2.2176	0.9968
37	284.97	280.13	58.8025	1.4732
38	291.23	286.27	18.4264	3.2920
39	295.73	290.70	13.6056	0.7516
40	305.87	300.67	2.1297	2.2467
41	314.45	309.10	144.6202	2.1168
42	315.92	310.55	117.3088	0.9133
43	320.96	315.50	43.8011	1.0212
44	328.57	322.98	42.7852	0.2599

45	333.88	328.20	79.1363	4.8924
46	340.72	334.93	37.0052	3.5044
47	342.47	336.65	4.9147	6.9713
48	351.52	345.54	17.3918	4.1557
49	363.42	357.24	2.4776	9.5504
50	380.18	373.71	10.9432	4.4902
51	397.62	390.86	128.7720	1.6465
52	400.00	393.20	98.0833	3.8333
53	417.68	410.58	15.9974	0.5897
54	430.17	422.85	17.6076	6.7911
55	432.67	425.31	13.5604	2.2782
56	435.27	427.87	7.0009	1.7969
57	447.47	439.86	6.9137	7.7885
58	461.38	453.54	2.4769	3.1470
59	479.07	470.93	1.9008	2.8493
60	485.79	477.53	188.1349	4.2372
61	503.85	495.28	13.4541	9.2287
62	517.67	508.87	8.9036	3.2545
63	547.43	538.12	18.3773	4.4964
64	555.07	545.63	128.1239	3.2638
65	559.54	550.03	8.0013	14.6047
66	567.67	558.02	88.0771	3.3084
67	569.80	560.11	24.8886	2.8182
68	583.02	573.10	43.5889	2.1159
69	600.17	589.97	9.3399	17.9240
70	606.19	595.89	9.8428	6.8686
71	611.43	601.04	16.0748	3.8014
72	620.54	609.99	60.1224	1.9925
73	632.91	622.15	69.6183	4.1685
74	638.73	627.87	34.6423	4.1842
75	640.58	629.69	30.0593	1.5335
76	657.18	646.01	35.7059	0.3875
77	692.09	680.32	18.5229	4.4748
78	708.89	696.84	137.1687	0.7024
79	716.96	704.77	15.7141	3.6845
80	740.71	728.12	16.3611	18.5809
81	756.56	743.70	48.8868	5.3131
82	769.11	756.04	25.3459	1.3477
83	770.72	757.62	56.3495	2.3342
84	778.02	764.80	23.8137	0.1767
85	795.99	782.46	63.4989	4.2214
86	839.05	824.79	474.0210	8.6669
87	854.66	840.13	187.9790	15.2637
88	868.84	854.07	450.9798	48.6471
89	899.01	883.72	3.2090	2.3442
90	917.57	901.97	60.7744	5.5065
91	919.99	904.35	5.2656	1.5746
92	939.09	923.12	67.4501	1.9773
93	965.30	948.89	24.5451	8.4889
94	968.53	952.06	11.7354	1.6154
95	985.85	969.09	136.3471	5.1929

96	996.21	979.27	165.6033	2.7913
97	1001.50	984.47	57.5547	0.9630
98	1003.84	986.78	103.6817	2.5793
99	1017.24	999.95	33.5593	1.7363
100	1028.01	1010.53	331.3773	1.9369
101	1035.72	1018.12	112.9346	1.7389
102	1039.97	1022.29	408.5875	3.4429
103	1044.26	1026.51	75.4148	1.9605
104	1049.73	1031.89	322.4676	3.7539
105	1056.93	1038.97	224.1076	2.1203
106	1059.22	1041.22	621.8126	1.2335
107	1064.76	1046.66	64.2203	19.0986
108	1068.35	1050.19	50.5581	7.5451
109	1073.20	1054.96	87.1073	15.7892
110	1086.98	1068.50	247.2424	1.9687
111	1090.04	1071.51	84.3660	8.1962
112	1094.21	1075.61	10.3847	15.2033
113	1106.38	1087.57	145.5929	12.1495
114	1107.66	1088.83	65.4236	20.3298
115	1113.82	1094.88	110.0627	12.2341
116	1115.39	1096.43	45.5207	9.4464
117	1126.58	1107.42	27.8853	3.3184
118	1127.52	1108.35	44.8223	3.1651
119	1129.20	1110.00	7.3291	14.5675
120	1135.94	1116.63	11.2700	7.0141
121	1140.41	1121.02	19.6580	12.7934
122	1146.66	1127.16	49.3104	4.3509
123	1160.10	1140.37	107.2334	10.0794
124	1169.35	1149.47	87.1015	4.3773
125	1177.67	1157.65	33.1527	7.7284
126	1203.51	1183.05	44.0358	5.6920
127	1204.07	1183.60	32.8943	7.9920
128	1212.47	1191.85	480.7674	1.9142
129	1227.58	1206.71	41.5139	2.3184
130	1237.34	1216.31	6.5592	6.7296
131	1250.92	1229.66	125.5436	1.8447
132	1272.02	1250.39	30.0320	3.3469
133	1275.02	1253.34	70.7996	7.0843
134	1280.39	1258.62	4.8591	3.6768
135	1287.00	1265.12	34.4168	6.0247
136	1297.35	1275.30	16.9081	8.0416
137	1301.69	1279.56	50.7400	2.5501
138	1302.27	1280.13	8.9160	2.4812
139	1305.49	1283.30	283.8748	23.9971
140	1310.68	1288.40	325.1872	8.6861
141	1316.87	1294.48	20.0551	5.4253
142	1323.36	1300.86	74.9183	34.1503
143	1330.02	1307.41	56.9128	9.8857
144	1334.90	1312.21	62.4203	10.2664
145	1338.60	1315.85	30.8123	0.8853
146	1341.18	1318.38	28.5338	8.0627

147	1346.92	1324.02	15.2457	24.7385
148	1354.23	1331.21	30.2807	2.9107
149	1354.42	1331.40	63.8302	9.2512
150	1371.44	1348.12	10.1273	7.8857
151	1378.96	1355.51	84.2667	8.2376
152	1383.20	1359.69	50.7509	17.7705
153	1390.53	1366.89	3.3863	7.9874
154	1392.01	1368.34	13.6705	8.8167
155	1399.55	1375.76	36.8917	4.2643
156	1403.32	1379.46	4.7213	35.5028
157	1406.46	1382.55	17.5974	9.8440
158	1407.55	1383.62	26.8182	16.8912
159	1411.24	1387.25	64.0837	4.4030
160	1413.89	1389.85	7.5612	8.1699
161	1417.88	1393.78	55.3875	4.0270
162	1424.90	1400.68	45.9698	7.7456
163	1427.15	1402.89	25.7988	5.9232
164	1430.33	1406.02	24.6038	6.0636
165	1435.90	1411.49	12.2874	7.5568
166	1448.68	1424.05	2.5943	6.3623
167	1458.13	1433.35	7.7219	17.4442
168	1461.64	1436.79	46.6143	2.9518
169	1465.50	1440.58	85.5937	8.5739
170	1472.61	1447.57	50.7468	2.9523
171	1500.18	1474.68	14.9932	14.7521
172	1505.44	1479.85	3.0687	9.5450
173	1513.39	1487.66	0.8272	13.4003
174	1679.01	1650.47	637.5250	4.1658
175	1682.46	1653.86	690.9725	6.0482
176	1698.79	1669.91	741.1064	6.2996
177	1813.29	1782.47	574.4928	33.6323
178	2945.23	2895.16	50.7478	192.0747
179	2968.34	2917.88	16.7458	114.4656
180	2977.46	2926.84	29.8655	355.0690
181	2978.09	2927.46	8.9399	47.1474
182	2979.30	2928.65	117.4437	453.4011
183	2985.55	2934.80	49.6230	365.4367
184	2994.17	2943.27	66.0536	77.8899
185	3003.12	2952.07	117.8794	24.2318
186	3031.51	2979.97	38.0833	74.3502
187	3049.05	2997.22	43.2883	296.0562
188	3051.09	2999.22	57.4019	161.9759
189	3057.88	3005.89	11.5308	157.3809
190	3063.97	3011.88	40.1595	170.2786
191	3076.93	3024.62	55.2699	200.5445
192	3086.07	3033.60	15.0748	314.1465
193	3092.94	3040.36	12.7728	267.6863
194	3103.37	3050.62	31.6983	130.7412
195	3105.12	3052.33	5.7597	67.4638
196	3122.80	3069.72	4.2924	13.4572
197	3146.81	3093.31	0.7618	100.4502

198	3555.35	3494.91	841.2891	316.2579
199	3629.39	3567.69	713.6396	307.6460
200	3718.48	3655.26	233.2841	117.3693
201	3769.27	3705.20	76.4977	169.5703
202	3782.96	3718.65	44.0657	113.1376
203	3790.80	3726.36	55.8463	112.9841
204	3809.38	3744.62	72.2390	117.7058

Table S13. Optimized geometry for the Model 6.

C	-0.89989500	1.26378700	-0.30064400
C	-6.16388500	1.09407800	-0.64831000
H	-0.94826600	1.49609200	-1.37425400
C	0.17984600	2.09922200	0.38695500
C	-5.48283600	-0.06662000	0.07391200
H	0.11267500	1.90354400	1.45997500
H	-5.32485000	-0.85698400	-0.65950800
C	1.56075300	1.67277100	-0.11530900
C	-4.16113100	0.35632600	0.70997800
H	1.65785700	1.98570400	-1.16683200
H	-4.35452000	1.06899600	1.51458400
C	1.73172500	0.14834000	-0.08049100
C	-3.26098700	1.02305000	-0.33187700
H	1.79770500	-0.17656900	0.96317700
H	-2.98451700	0.29013000	-1.09401300
C	0.55578200	-0.56496300	-0.75407900
C	-3.99004800	2.14763000	-1.07104700
H	0.53844800	-0.30543200	-1.82204900
C	0.55838200	-2.09082600	-0.61380000
H	0.80044200	-2.37454900	0.41067000
H	-0.44942700	-2.44915600	-0.83884000
O	-0.04991600	3.48741700	0.23038100
O	-3.50550200	-0.68530100	1.45633800
O	-7.34505000	1.20691800	-0.72965000
O	-2.12689600	1.54311000	0.31663100
O	-0.65092500	-0.12251200	-0.12621800
O	-5.35978300	2.02661400	-1.26334700
O	1.51582300	-2.74605600	-1.44227900
H	0.12996600	3.73019600	-0.68674800
O	2.53115300	2.32283500	0.68406200
O	2.92186300	-0.22874200	-0.78114200
H	1.29861300	-2.58076600	-2.36776500
H	3.40829000	2.02932400	0.38197700
C	4.08783800	-0.42002700	-0.05060700
H	3.86067400	-0.69548200	0.98777900
O	4.81976400	0.81305700	-0.06437100
C	4.87891000	-1.54559500	-0.72570300
C	6.05670200	0.74303100	0.65858900
C	6.25654800	-1.65934900	-0.08358100
O	4.20004400	-2.77353900	-0.57799300
H	4.98938600	-1.27202700	-1.78541900
H	5.87252900	0.44092600	1.69779100

C	6.66647400	2.14351200	0.65478700
C	6.94358600	-0.29689400	-0.02719200
H	6.13536800	-2.02956700	0.93868000
O	7.10997000	-2.61188000	-0.71374800
H	3.30772900	-2.68639500	-0.96687900
O	7.79490700	2.23054400	1.51108700
H	5.92710800	2.85409900	1.02826900
H	6.91550700	2.42478300	-0.37667600
O	8.17193100	-0.39514500	0.69018600
H	7.13539400	0.04837000	-1.05309000
H	7.14533900	-2.41433900	-1.65905700
H	8.37234900	1.48525400	1.28722700
N	-3.02114300	-1.82614700	0.75166100
O	-3.27833000	-1.92873000	-0.42332700
O	-2.40745700	-2.56070900	1.46594500
H	8.65865200	-1.13778700	0.30607400
O	-6.33105100	-0.52798000	1.11777400
O	-3.44586500	3.10135500	-1.52493600
N	-7.26936600	-1.55616600	0.72522400
O	-7.22000900	-1.95980200	-0.40355700
O	-7.97910200	-1.86482800	1.63407000

Table S14. Calculated vibrational modes frequencies and activities for the Model 6.

Mode number	Calculated mode frequency, cm^{-1}	Scaled mode frequency, cm^{-1}	IR activity, KM/Mole	Raman activity, A^4/AMU
1	11.92	11.71	0.2868	0.2872
2	20.22	19.88	0.2283	0.5948
3	22.37	21.99	0.4005	0.3335
4	29.68	29.18	0.9135	0.1606
5	33.69	33.12	0.1538	0.6882
6	39.87	39.19	0.0170	1.0455
7	46.59	45.80	1.4907	0.8061
8	52.54	51.65	1.8046	1.8638
9	61.17	60.13	1.9756	0.1756
10	69.85	68.66	2.0895	0.1189
11	74.02	72.76	0.5292	3.5400
12	77.30	75.98	4.1525	2.9980
13	89.99	88.46	0.7254	0.5318
14	95.51	93.88	1.3949	0.1223
15	104.68	102.90	3.4118	0.3192
16	113.15	111.23	0.8295	3.6233
17	121.48	119.41	6.4475	0.9557
18	125.09	122.96	6.4203	0.9419
19	129.25	127.06	2.8229	1.3695
20	146.66	144.16	2.4691	0.7685
21	153.39	150.78	2.0850	0.5471
22	154.60	151.97	12.1298	0.3437
23	170.76	167.86	3.1647	0.6050
24	195.41	192.09	1.7626	1.2169
25	197.00	193.65	12.4548	1.3610
26	205.01	201.53	3.5535	0.0776

27	219.84	216.10	10.3999	0.2498
28	236.65	232.63	105.9131	2.3930
29	240.78	236.68	10.0981	4.7772
30	247.94	243.73	4.2419	0.5816
31	258.59	254.19	28.2716	1.2640
32	276.75	272.04	3.9654	1.5541
33	291.19	286.24	17.4365	2.9036
34	297.06	292.01	9.8207	0.6514
35	305.70	300.50	7.4514	1.3948
36	312.11	306.80	79.0621	2.7885
37	312.92	307.60	76.1117	1.1450
38	315.50	310.14	52.8376	0.6701
39	328.92	323.33	145.4879	3.0516
40	329.22	323.63	67.5155	2.6857
41	338.84	333.08	10.7834	4.9643
42	346.61	340.71	23.6220	7.0101
43	350.14	344.18	3.9365	6.5423
44	377.51	371.10	0.7716	5.2288
45	385.19	378.64	12.9852	4.5560
46	395.55	388.83	217.2989	4.2748
47	398.34	391.57	5.7911	1.7003
48	408.24	401.30	32.6657	3.3639
49	429.76	422.45	21.7183	6.3328
50	430.95	423.62	6.4487	3.6591
51	442.58	435.05	9.7557	5.5813
52	444.79	437.23	1.7628	7.8995
53	464.66	456.77	1.8951	3.5284
54	480.92	472.75	3.0043	7.5677
55	483.92	475.69	193.8696	4.6886
56	510.12	501.44	14.9240	3.8530
57	515.46	506.70	5.3778	3.6827
58	551.39	542.02	20.3398	0.0766
59	559.08	549.58	56.1641	1.4423
60	559.74	550.23	86.4577	1.2015
61	568.48	558.81	68.8129	22.0592
62	571.49	561.78	72.7045	3.5678
63	587.13	577.15	32.2180	1.8637
64	606.69	596.38	11.8591	4.1896
65	623.68	613.08	50.4637	3.1055
66	632.61	621.85	41.4148	3.5013
67	639.76	628.88	46.7757	1.4711
68	654.21	643.09	65.2543	3.3055
69	657.81	646.62	4.5775	4.2018
70	697.33	685.48	17.0856	4.6545
71	703.96	691.99	9.3271	4.3816
72	705.42	693.43	147.3891	1.0587
73	720.57	708.32	55.5479	3.2344
74	743.49	730.85	12.3172	11.6660
75	762.89	749.93	21.4720	0.7587
76	768.30	755.23	37.0768	1.5195
77	794.45	780.95	23.3522	1.0678

78	826.99	812.94	450.2049	20.0891
79	845.52	831.15	402.7899	22.5412
80	899.56	884.27	2.9924	2.1912
81	919.34	903.71	4.6344	1.4238
82	925.04	909.31	101.8928	8.9784
83	963.40	947.03	46.8214	2.7613
84	969.14	952.67	4.5623	2.5551
85	994.16	977.26	301.9170	5.1809
86	1000.60	983.59	145.5759	1.0718
87	1000.66	983.65	92.4567	4.3696
88	1004.11	987.04	15.0657	2.4941
89	1017.27	999.98	43.0773	2.7059
90	1029.76	1012.25	333.2644	1.8013
91	1038.99	1021.33	403.2224	2.7945
92	1039.79	1022.11	912.9572	3.4932
93	1048.25	1030.43	568.4293	1.1250
94	1055.84	1037.89	200.5865	1.6463
95	1061.54	1043.50	22.9719	12.1777
96	1066.96	1048.82	190.2704	1.9854
97	1068.83	1050.66	150.4532	10.8426
98	1070.76	1052.56	385.1253	3.3191
99	1074.50	1056.23	46.2201	16.1674
100	1092.61	1074.04	21.3152	4.9737
101	1100.32	1081.61	70.7534	12.8409
102	1105.64	1086.85	79.5545	23.5901
103	1108.22	1089.38	222.5496	12.3534
104	1114.41	1095.47	62.5216	11.3630
105	1117.02	1098.03	43.4740	8.3602
106	1127.23	1108.07	62.2947	3.2501
107	1128.52	1109.33	20.0491	10.3780
108	1135.44	1116.14	2.2258	5.4261
109	1141.82	1122.41	54.3712	2.6292
110	1147.62	1128.11	17.0742	7.3046
111	1157.87	1138.18	29.2290	23.0937
112	1165.31	1145.50	157.1255	5.3072
113	1177.31	1157.30	35.8245	7.5101
114	1203.88	1183.41	2.0935	10.1693
115	1204.57	1184.10	54.0638	3.0590
116	1210.22	1189.64	217.1967	1.2503
117	1225.45	1204.62	44.3396	2.6851
118	1233.88	1212.90	2.1445	4.8796
119	1237.84	1216.79	5.1570	5.7351
120	1272.28	1250.65	32.0368	3.2460
121	1275.82	1254.13	18.0424	3.4883
122	1276.18	1254.48	68.0022	6.6138
123	1295.58	1273.56	14.3628	5.7908
124	1300.57	1278.46	35.1971	0.8215
125	1302.90	1280.75	29.1070	10.6278
126	1315.63	1293.26	6.8603	3.2233
127	1318.52	1296.11	191.6819	19.6236
128	1320.85	1298.40	78.9179	14.7723

129	1325.59	1303.06	292.5321	18.6868
130	1334.47	1311.78	7.2688	4.3674
131	1335.76	1313.05	21.9777	1.8570
132	1339.13	1316.36	27.3201	7.1048
133	1346.93	1324.03	9.6476	27.3047
134	1354.08	1331.06	16.6179	5.7457
135	1367.31	1344.07	33.9665	7.0941
136	1370.91	1347.60	28.1783	13.3792
137	1387.70	1364.11	41.1842	11.5793
138	1389.20	1365.59	52.8690	7.7563
139	1390.72	1367.08	4.3547	4.8117
140	1395.71	1371.98	27.9242	11.6743
141	1400.46	1376.65	0.3940	31.6935
142	1406.31	1382.40	49.1832	7.0187
143	1407.66	1383.73	7.6338	18.4396
144	1420.27	1396.12	46.2018	7.8624
145	1421.50	1397.33	67.9007	6.3722
146	1427.74	1403.47	31.0646	10.1788
147	1428.67	1404.38	33.8293	5.5727
148	1435.98	1411.57	11.4794	8.3378
149	1443.85	1419.30	1.3683	3.7065
150	1445.64	1421.07	18.9464	20.5799
151	1461.03	1436.20	28.0033	4.3136
152	1464.32	1439.42	88.8280	10.1475
153	1471.82	1446.80	69.6482	2.6426
154	1507.16	1481.54	2.7371	9.7662
155	1513.18	1487.46	0.8982	13.6112
156	1693.14	1664.36	662.1314	4.9430
157	1716.38	1687.20	700.5872	4.9468
158	1825.71	1794.67	684.9030	18.4509
159	1881.38	1849.39	230.0178	69.6123
160	2950.73	2900.57	46.3162	165.8589
161	2970.17	2919.68	11.8570	77.1571
162	2975.64	2925.05	34.7960	405.7959
163	2976.57	2925.97	70.8256	206.0459
164	2981.11	2930.43	70.7795	291.7611
165	2985.35	2934.60	51.5804	369.7230
166	2992.90	2942.02	54.2883	80.8209
167	3002.78	2951.74	123.2818	27.8685
168	3030.27	2978.76	44.5329	71.7407
169	3041.09	2989.39	52.7862	287.0380
170	3050.21	2998.36	55.1536	158.4427
171	3066.33	3014.20	39.1551	163.4991
172	3068.69	3016.53	14.6130	159.4460
173	3077.07	3024.76	54.8913	202.0033
174	3097.07	3044.42	5.9823	197.7220
175	3101.94	3049.21	30.7779	115.3428
176	3109.63	3056.76	20.9342	12.0063
177	3559.16	3498.66	827.7619	314.8786
178	3636.31	3574.49	686.4796	301.2925
179	3718.43	3655.21	232.8532	117.4409

180	3768.82	3704.75	77.1235	169.1740
181	3782.48	3718.18	42.9721	112.0422
182	3790.95	3726.51	49.5968	120.4278
183	3807.80	3743.06	74.9312	120.3837

The example of gluconolactone and anhydride degradation Models (the case of Model 5 and 6).

Articles [19, 30] demonstrate the mechanism of cellulose nitrate degradation via chain scission. This results in the formation of gluconalactone and anhydride degradation products. Therefore, we additionally calculated the vibrational spectra for an example of the gluconalactone structure and an example of the anhydride structure. These cases are referred to as Model 5 and Model 6, respectively. Due to chain scission and shortening, it was decided to use shorter Models consisting of three pyranose rings. Their optimized structure, calculated using the same theoretical approach as in Section 3.1, is presented in Figure S13.

(a)

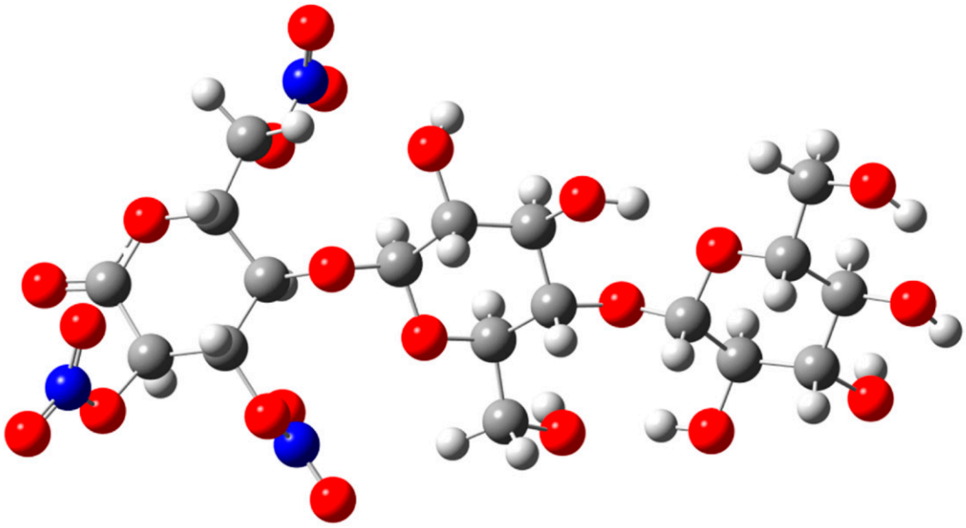

(b)

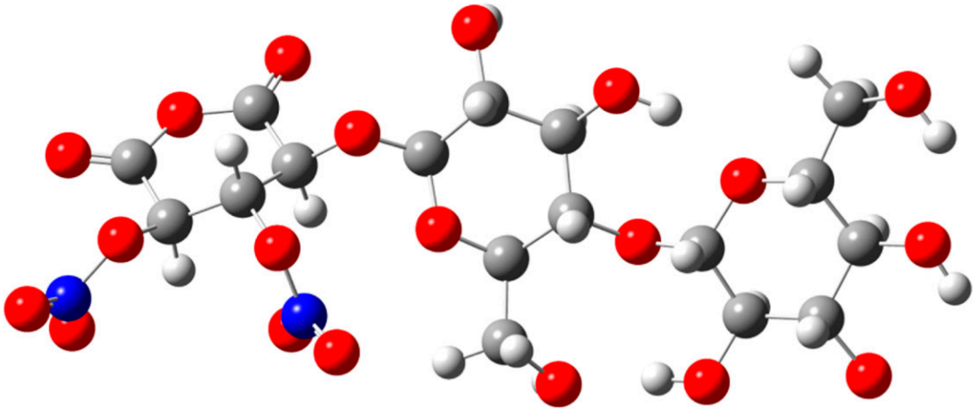

Figure S13. Optimized geometries of the Model 5(a) and Model 6(b).

The calculated IR absorption spectra are demonstrated in Figure S14 for Model 5 and 6. In the region of C-H stretching vibrations, the situation is qualitatively similar to those in Models 2-4.

As in Models 2-4, the appearance of the C=O bond leads to additional absorption peaks above 1700 cm^{-1} in Models 5 and 6. For example, in the gluconolactone case (the corresponding Model 5), the presence of a single absorption peak was noted, which corresponds to the formation of a single C=O bond. Its scaled frequency is 1782 cm^{-1} (mode 177). On the other hand, in the anhydride case (Model 6), there are two vibrational modes, which correspond to oxygen atomic displacements in double carbon-oxygen bonds.

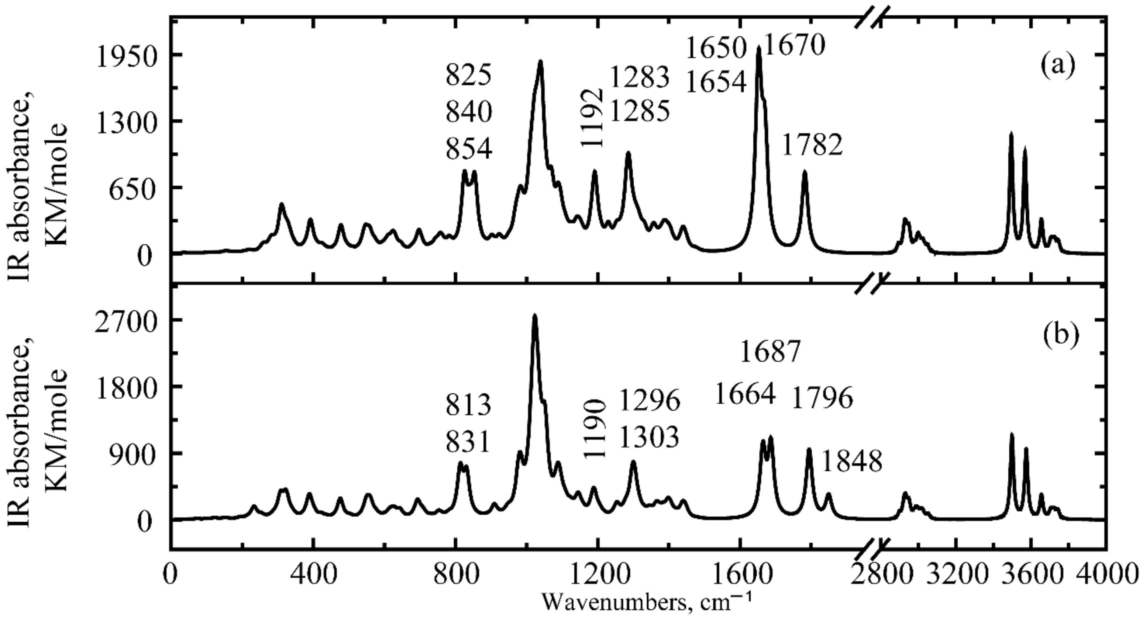

Figure S14. Calculated scaled infrared (IR) absorption spectra for the Model 5(a) and Model 6(b). The theoretical approach is B3LYP/6-311G(2d,p) with water parameters PCM solvation Model. The scaling factor is 0.983.

One of them is in-phase (symmetric) stretching displacements(v(C=O)). It's frequency is about 1849 cm^{-1} (mode 159). Another one (mode 158) has lower frequency about 1795 cm^{-1} and it is antisymmetric stretching displacements. Its predicted absorption is significantly higher.

In the [19, 30] the spectral region 1735–1740 cm^{-1} was determined for gluconolactones and 1760 cm^{-1} for the anhydrides. It cannot be ruled out that the influence of the environment on the model structures in this work may be partially insufficient. This, in turn, may lead to an overestimation of the stretching vibration frequency. However, it should be noted that with long irradiation times of the sample (225 h), the formation of a shoulder in the region of 1800 cm^{-1} was noted in the experimental spectra in [30]. In general, it should be noted that the estimates for the frequencies of v(C=O) vibrations obtained in the calculation are in the correct ratio. The v(C=O) frequencies in the anhydride case are higher than the vibration frequencies in the gluconolactone case. At the same time, the v(C=O) mode in the gluconolactone case and the antisymmetric vibrational mode in the anhydride case have a comparable frequency to v(C=O) in Model 2. On the other hand, Models 3 and 4 have a lower frequency of the corresponding modes.

Differences with ketone Model structures 2-4 are also related to the shape of the antisymmetric stretching vibration peak of the NO_2 group (v_{as}(NO2)). For the gluconolac-

tone and anhydride cases, a significant increase in the frequency of the mode (No. 176 and 157) to 1670 and 1687 cm^{-1}, respectively, is observed. The antisymmetric vibrations in this mode are localized in the nitrate group closest to the oxygen-carbon double bond (Figure S15). Experimentally, this can be manifested by the appearance of an additional shoulder initially and then by a general increase in the peak width toward higher wavenumbers. This allows it to be used as a basis for identifying the chain scission process.

The remaining two modes (174-175) in the gluconolactone case have frequencies of 1650 and 1654 cm^{-1}, lower than those of mode 156 (1664 cm^{-1}) in the anhydride case (Figure S15). They fall within the region where similar vibrational modes from ketone structures (Models 2-4) appear.

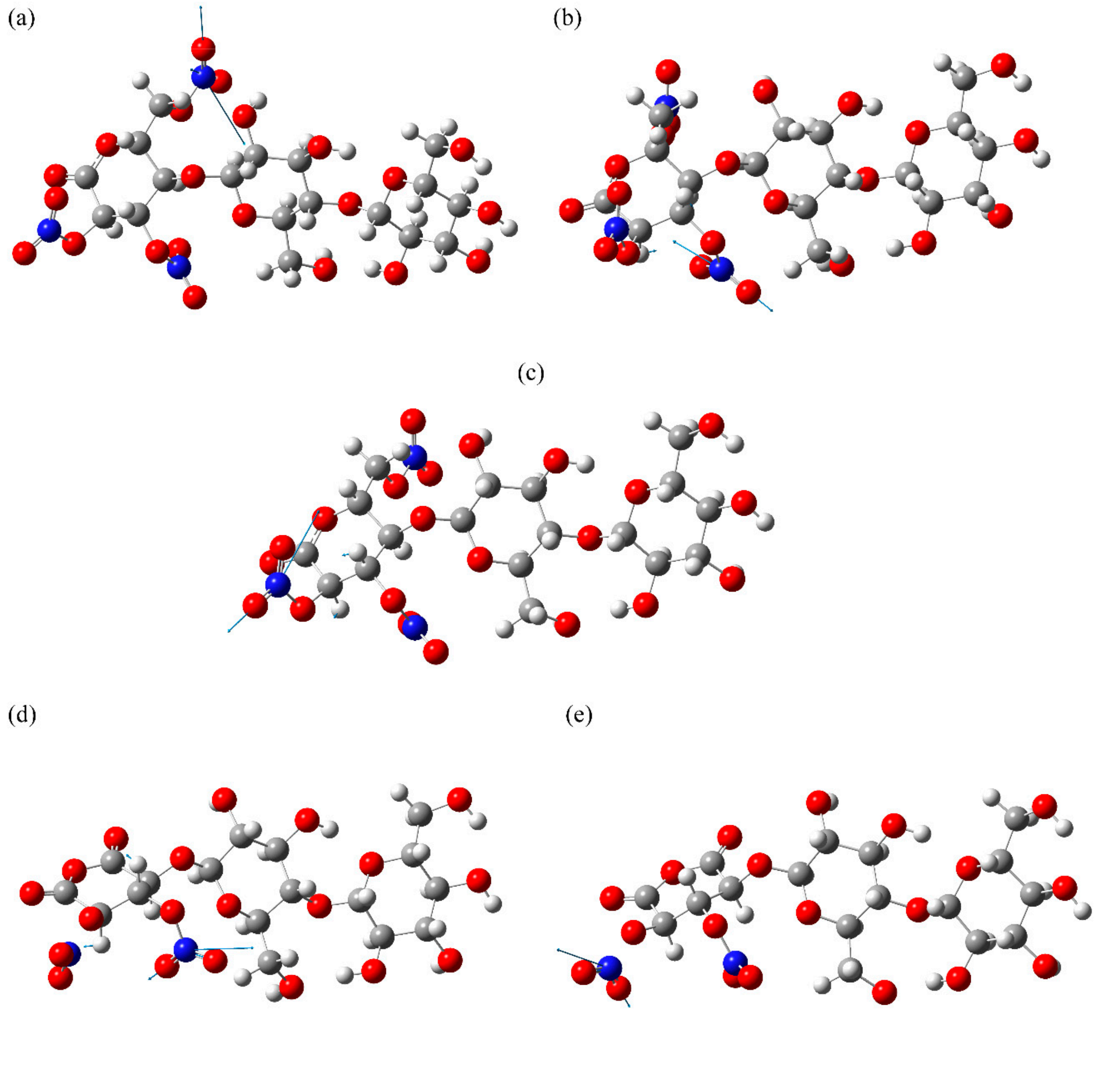

Figure S15. Antisymmetric atomic displacements in modes 174 (a), 175 (b) and 176 (c) for Model 5 as well as antisymmetric atomic displacements in modes 156 (d) and 157 (e) for Model 6.

Modes 139 and 140, which include not only hydrogen bending vibrations but also the contribution of symmetric bending vibrations of the NO_2 group, have frequencies of 1283 and 1285 cm^{-1} for Model 5. For the anhydride case (Model 6), the frequencies of the corresponding modes 127 and 129 are higher and equal 1296 and 1303 cm^{-1}.

Also, in Models 5 and 6, significant splitting is observed for modes combining the stretching vibration of NO and the bending vibration of the NO_2 group. For Model 5 (the gluconolactone case), these modes are 86, 87, and 88 (Figure S16). The corresponding scaled frequencies are 825, 840, and 854 cm^{-1}. In turn, for Model 6 (the anhydride case), the presence of two modes, 78 and 79, with a split of 18 cm^{-1}, is predicted. By analogy, in Model 6, the lower frequency of 813 cm^{-1} is that of mode 78 with the largest atomic displacements in the nitrate group closer to the double bond, while the higher frequency of 831 cm^{-1} is that of the mode with atomic displacements in the other nitrate group, see Figure S16.

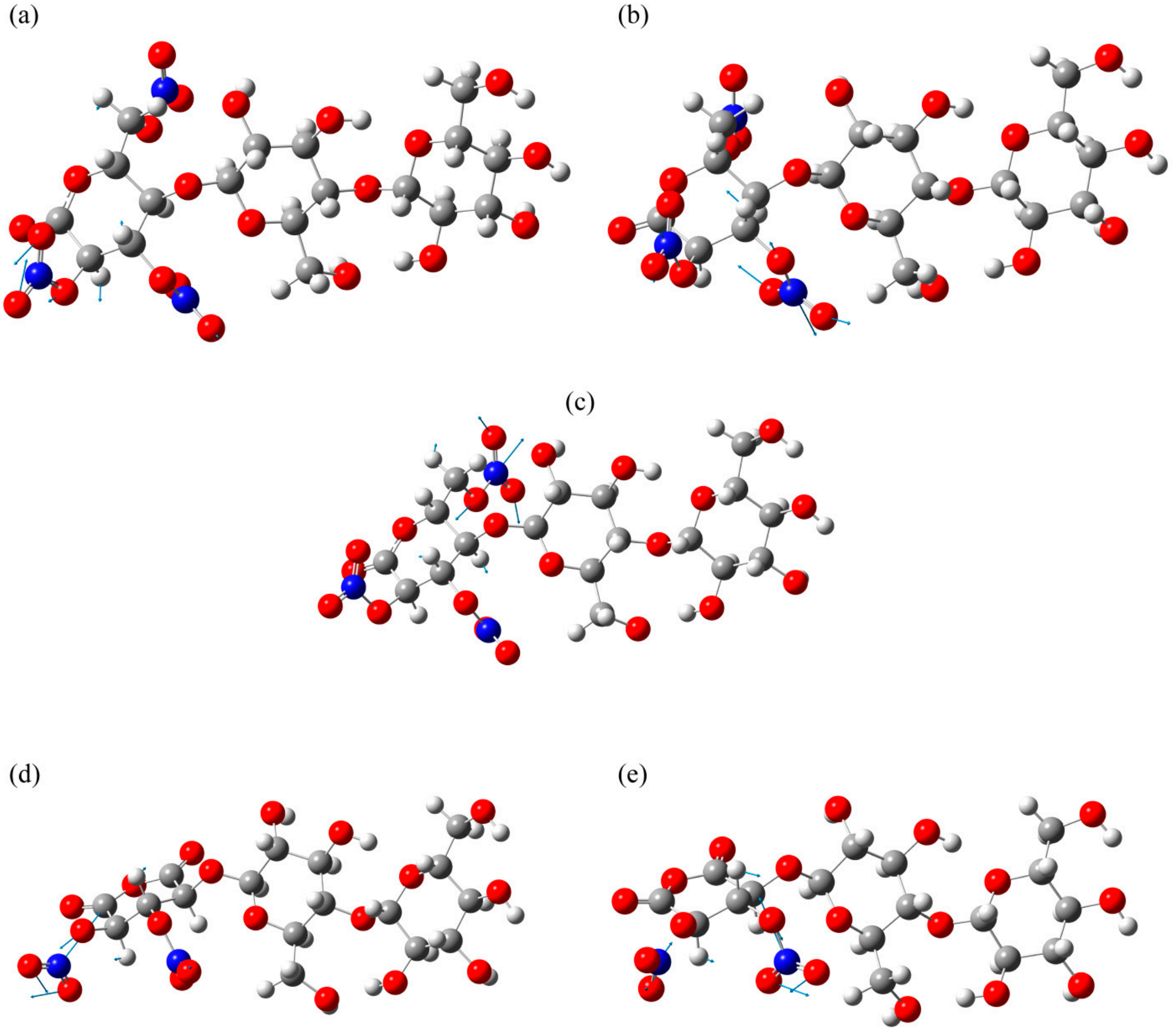

Figure S16. Amplitudes of atomic displacements in modes 86(a), 87(b) and 88(c) for Model 5 and in modes 78(d) and 79(e) for Model 6.

Similar to Models 1–4, due to the relatively small number of nitrate groups, the IR absorption spectrum receives a significant contribution from the pyranose rings. This contribution manifests itself primarily as stretching vibrations in single C–O bonds within the ring and at the boundary in the range of 970–1100 cm^{-1}.

Models 5 and 6 predict the presence of modes 128 and 116 in the IR absorption spectrum, with scaled frequencies of 1192 and 1190 cm^{-1}, respectively. These modes correspond, among other things, to stretching vibrations in the C–O bond within the pyra-

nose ring, where carbon has a double bond with oxygen (see Figure S17). These modes can be considered characteristic. In [30], it can be noted in the spectra that at an advanced stage of degradation, there is a shift from the peak maximum at 1160 cm–1 toward higher wavenumbers of 1175 cm^{-1}, with a shoulder from the side of the greatest values.

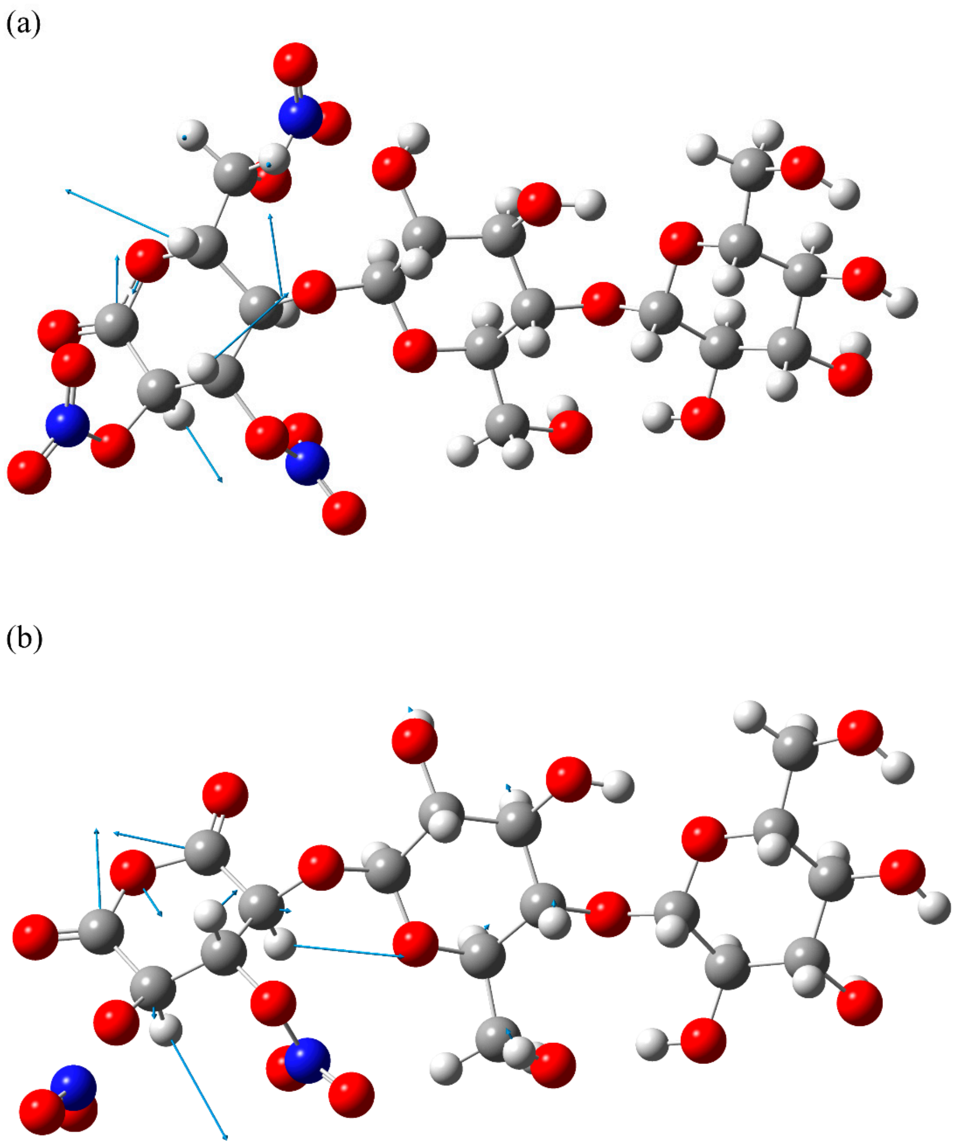

Figure S17. Atomic displacements in mode 128(a) of Model 5 and mode 116(b) of Model 6.

The calculated Raman spectra for Models 5 and 6 are shown in Figure S18. The calculation was performed in a similar way to that for Models 1–4 and the NC Model. The greatest difference from Models 1–4 for Model 5 was in the region of the peaks at 550 and 590 cm^{-1}, as well as 728 cm^{-1}. The peaks at 550 and 590 cm^{-1} are associated with modes 65 and 69 in Model 5 (Figure S19). Mode 65 in Model 5 is associated with atomic displacements of the CH_2NO_3 moiety as well as bending vibrations in the corresponding pyranose rings, including δ(OCC) and δ(CCC). In the case of mode 69 in Model 5, it is dealt with delocalized atomic displacements in CH_2NO_3 and one of the other NO_3 groups, as well as torsional vibrations in pyranose rings (tors(CCOC) and tors(CCCC)).

The peak at 728 cm^{-1} is associated with mode 80 (Figure S19), in which atomic displacements are localized in the boundary ring. These can be divided into stretching vibrations ν(NO) and deformation vibrations δ(NO2) in CH_2NO_3, as well as deformation vibrations in the pyranose ring and at its boundary.

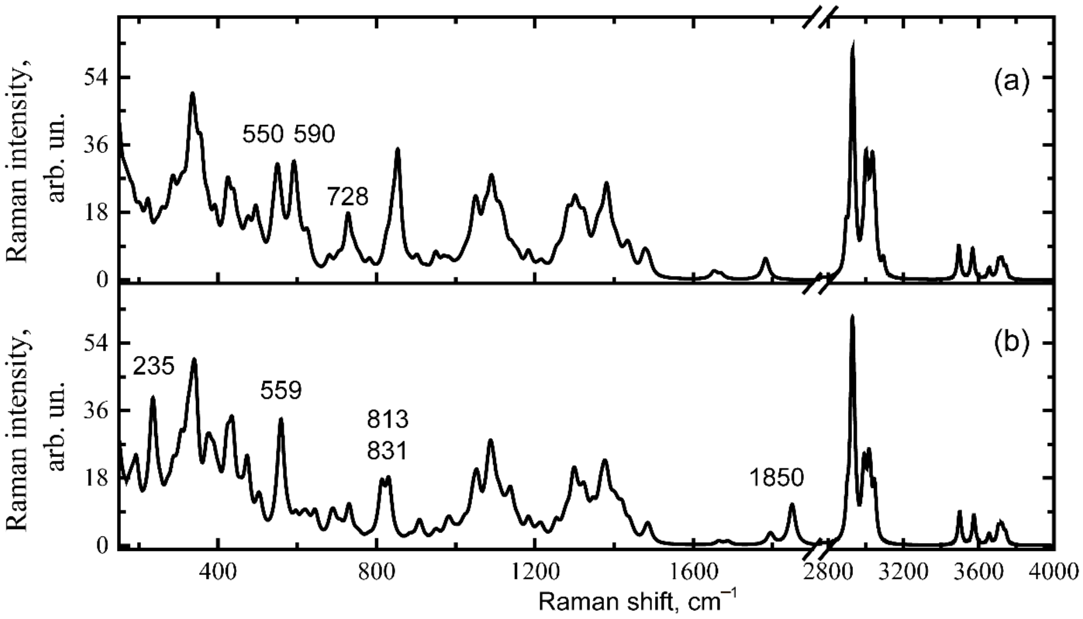

Figure S18. Calculated scaled Raman spectra for Model structures 5 (a) and 6 (b). The theoretical approach is B3LYP/6-311G(2d,p) with water parameters PCM solvation model. The scaling factor is 0.983.

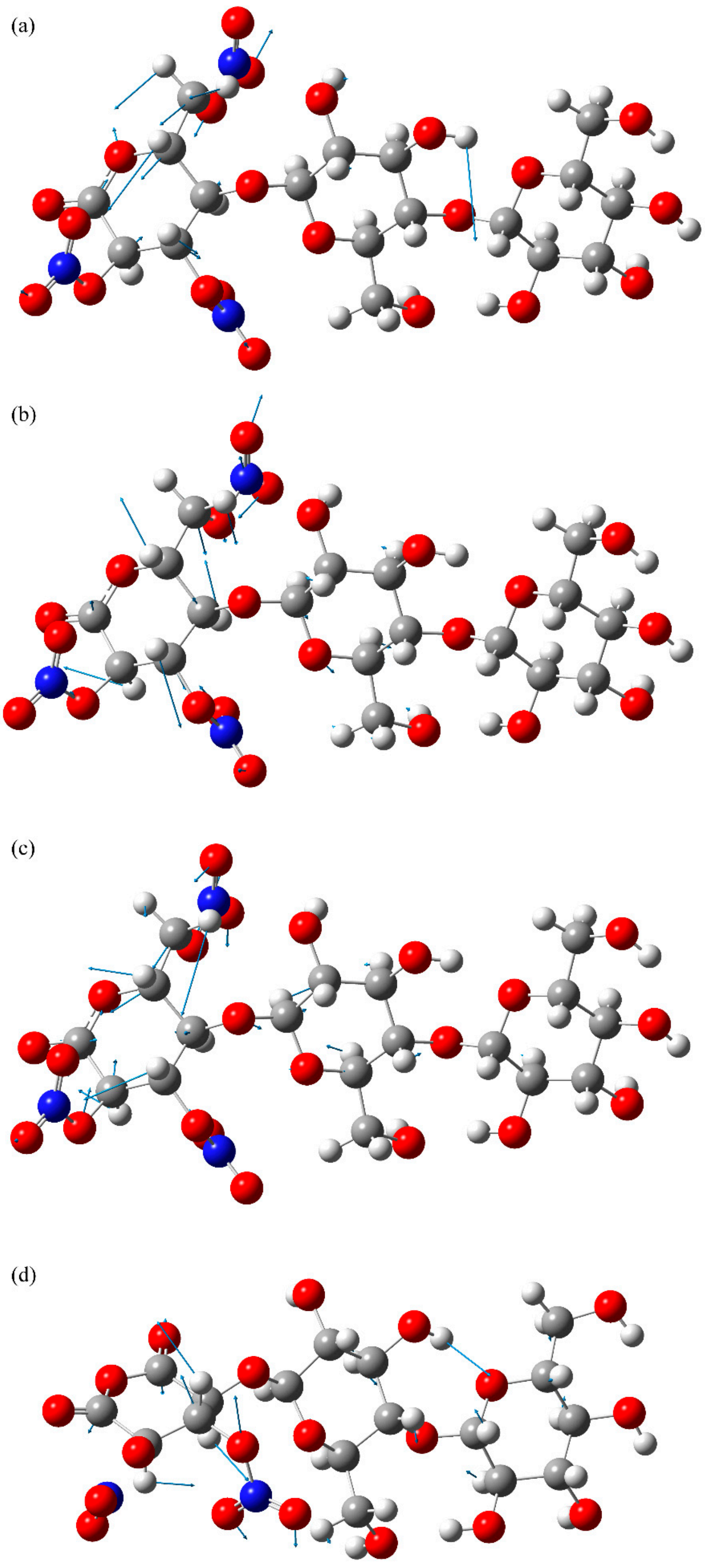

Figure S19. Atomic displacements in vibrational modes 65(a), 69(b), 80(c) for Model 5 as well atomic displacements in vibrational mode 61(d) for Model 6.

Similarly, in the nearby range, Model 6 exhibits a peak at 559 cm^{-1}, which differs sharply from the peaks for Models 1-4 in this range. This peak is also associated with out-of-plane vibrations in the pyranose ring, as well as ν(NO) vibrations of the water atoms of the nitrate groups attached to this ring (see atomic displacements in mode 61 of Model 6). In the lower-frequency region, Model 6 is predicted to exhibit a peak at 234 cm^{-1}, formed by vibrational modes 11 and 12 associated with delocalized torsional and deformational atomic displacements.

Furthermore, the Raman spectra for Model 6 exhibit modes also present in the IR spectrum, namely, peaks at 813 and 831 cm^{-1}, as well as 1850 cm^{-1}.

Influence of solvent parameters

Figure S20 presents a comparison of two calculated IR absorption spectra, scaled with the same factor, obtained for the Model 1 with four fully substituted pyranose rings, i.e., the limiting case of maximum substitution (γ=3). It was found that for a less polar solvent—exemplified in Figure 1b by methanol (ε=32.613) as compared with water (ε=78.3553) [35]—the main effect is observed for the strongly polar ν(N=O) stretching vibrations. This effect is relatively weak: the band maximum in the methanol solution is only 2 cm^{-1} higher than that in the aqueous solution. Thus, a decrease in solvent polarity has a relatively minor influence even on the most polar nitrate groups at full substitution (γ=3). Nevertheless, it should be noted that the shift toward lower frequencies with increasing solvent polarity does bring the calculated values closer to the experimentally observed frequency.

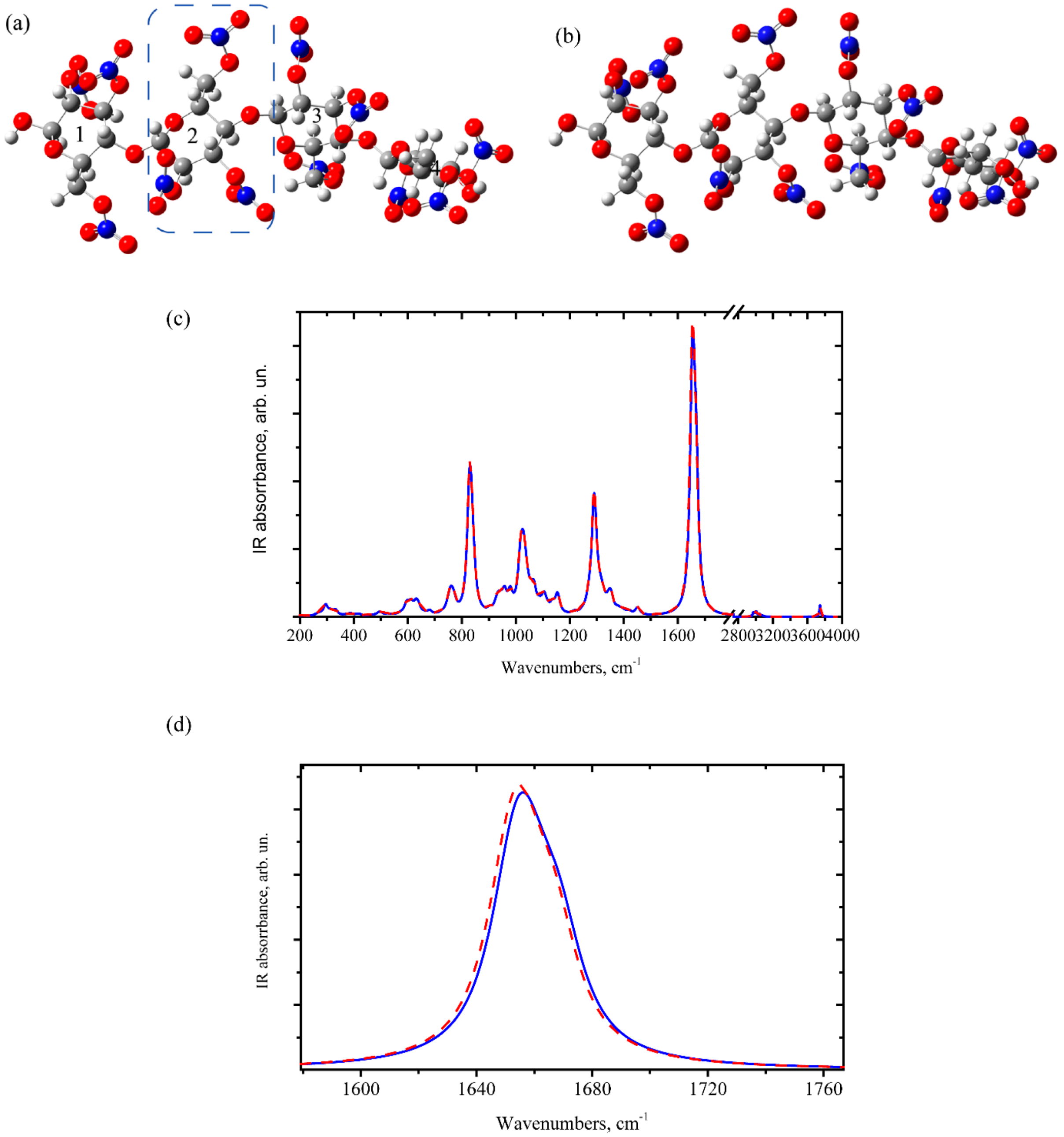

Figure S20. a – structure image of the optimized geometries of the NC Model in water, b - structure image of the optimized geometries of the NC Model in methanol solvent taken from the same view as in case (a), c - the scaled calculated IR absorbance spectra of NC Model in water (red dashed line) and methanol (blue solid line) solutions, d – the enlarged part of the (c) spectra.

However, as mentioned earlier, due to the incomplete degree of substitution of OH groups by NO_3 groups in the pyranose rings, which can occur in practice, we additionally investigated the effects of solvent type with different polarities on the Model structures. It was found that relatively substantial differences are obtained between calculations performed with water and methanol as solvents. In addition, for comparison, we present the results of calculations performed in a solvent with a very low dielectric constant—o-cresol (ε = 6.76)—corresponding to the case of a low-polarity solvent. The results are presented in Figure S21.

For the absorption spectra in aqueous and methanol solutions, relatively minor differences are observed. For most polar bands, the position of the maximum shifts with-

in 2 cm^{-1}. The largest shifts are noted for the stretching vibrations of the carbon–oxygen and nitrogen–oxygen double bonds (Figure S21).

In addition, we performed calculations of the IR absorption spectra for the low-polarity solvent o-Cresol (ε=6.76). In comparison with water, more substantial changes are observed. Specifically, shifts to higher frequencies are noted for the polar vibrational modes involving the carbon–oxygen and nitrogen–oxygen double bonds, with shifts in the range of +6 to +9 cm^{-1}. Comparison with the experimental frequencies reported in the work of [30] shows that for the initial stages of degradation, the position of the $\nu_{as}(NO_2)$ peak is closer to the values obtained from calculations in aqueous solution, in which the frequency of the corresponding peak is maximally redshifted to values of approximately 1652–1655–1660 cm^{-1} upon transition from Model structures 2 to 4.

This suggests that the calculations performed in aqueous solution with the application of a scaling factor are closer to the experimental data and are therefore preferable.

For comparison, the vibrational frequencies obtained from the calculated spectrum in o-Cresol lie in the ranges of 1661–1663–1666 cm^{-1} upon transition from Model structures 2 to 4. A consistent blue shift toward higher wavenumbers is also observed for the C=O stretching vibration when moving from the aqueous solution (1790–1774–1768 cm^{-1}) to the o-cresol solution (1795–1780–1773 cm^{-1}) for structures 2–4, respectively.

For vibrations in other spectral regions, the changes predominantly involved a decrease in absorption intensity without any significant shift in the band maxima. Specifically, a decrease in absorption is observed in the region of polar stretching vibrations of oxygen–carbon single bonds upon transitioning from the aqueous solution to o-cresol. This decrease is associated with the stretching vibrations within the ring (the band around 1060 cm^{-1}) as well as with the stretching vibrations at the ring boundaries (the C–O boundary bonds, the band around 1030 cm^{-1}). It should also be noted that the choice of solvent affects the region of torsional vibrations involving OH groups, as well as deformation vibrations in which one of the atoms is oxygen. This leads to reduced absorption in the region below 600 cm^{-1}, which is especially noticeable in the 300–400 cm^{-1} range, where bending vibrational modes in CCO, OCO, and COC fragments are localized, among others.

In the high-frequency region, the dielectric constant of the solvent influences the stretching vibrations of C–H and O–H bonds, resulting in a decrease in absorption intensity as the solvent polarity decreases.

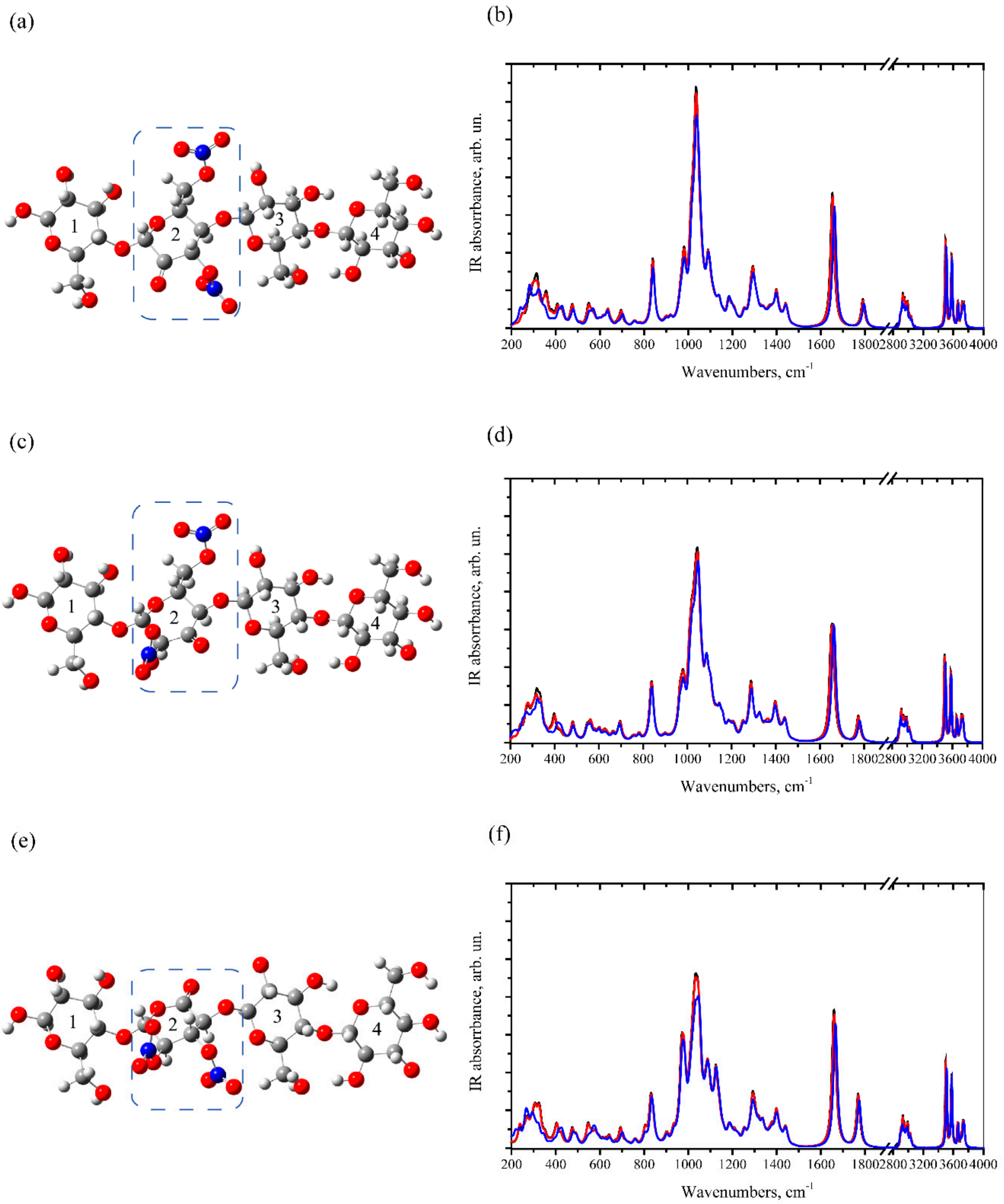

Figure S21. Example of optimized structural Model 2(a) and corresponding IR absorbance scaled spectra (b), structural Model 3 (c) and corresponding IR absorbance scaled spectra (d), structural Model 4(a) and corresponding IR absorbance scaled spectra (f) in water (black), methanol (red) and o-Cresol (blue) solvents.

References

19. Thérias S., Bussière P.-O., Gardette M., Gardette J.-L. et al. Degradation of celluloid in art works: a study of the mechanisms Paris, 20-21 November, 2012, 68-73.

30. Neves, Artur, et al. "New insights into the degradation mechanism of cellulose nitrate in cinematographic films by Raman microscopy." Journal of Raman Spectroscopy 50.2 (2019): 202-212.

35. Frisch, M.J.; Trucks, G.W.; Schlegel, H.B.; Scuseria, G.E.; Robb, M.A.; Cheeseman, J.R.; Scalmani, G.; Barone, V.; Mennucci, B.; Petersson, G.A.; et al. Gaussian 09, Revision C.01. Available online: https://gaussian.com/glossary/g09/ (accessed on 31 August 2025).
